# Avenue to novel *o*-carboranyl boron compounds – reactivity study of *o*-carborane-fused aminoborirane towards organic azides†

**DOI:** 10.1039/d4sc00489b

**Published:** 2024-02-22

**Authors:** Junyi Wang, Libo Xiang, Xiaocui Liu, Alexander Matler, Zhenyang Lin, Qing Ye

**Affiliations:** a Department of Chemistry, Southern University of Science and Technology 518055 Shenzhen P. R. China; b Department of Chemistry, The Hong Kong University of Science and Technology Clear Water Bay Kowloon Hong Kong chzlin@ust.hk; c Institute of Inorganic Chemistry, Julius-Maximilians-Universität Würzburg Am Hubland 97074 Würzburg Germany qing.ye@uni-wuerzburg.de; d Institute for Sustainable Chemistry& Catalysis with Boron, Julius-Maximilians-Universität Würzburg Am Hubland 97074 Würzburg Germany

## Abstract

Herein we report the reactivity study of *o*-carborane-fused bis(trimethylsilyl)aminoborirane towards three different types of organic azides, *i.e.*, aryl, alkyl, and silyl azides. The reaction with ArN_3_ (Ar = 2,6-iPr_2_C_6_H_4_, 2,6-C_6_H_3_Cl_2_, 2,4,6-C_6_H_2_Br_3_, C_6_F_5_) resulted in the cycloaddition of ArN_3_ to the borirane BN unit accompanied by silyl migration. Conversely, in the reaction with BnN_3_, only the BnN_3_ : borirane 1 : 2 ring expansion product was obtained. Finally, the reaction with Me_3_SiN_3_ resulted in a formal nitrene insertion product under thermal conditions. All of the newly obtained *o*-carborane-fused BN-containing heterocycles were fully characterized, and the mechanism of these substituent-dependent reactions was studied using DFT calculations.

## Abstract 2

A range of novel *o*-carboranyl substituted boron compounds have been synthesized through reactions between an *o*-carborane-fused aminoborirane and various organic azides. The reaction selectivity and mechanisms were studied using DFT calculations.

## Graphical abstract

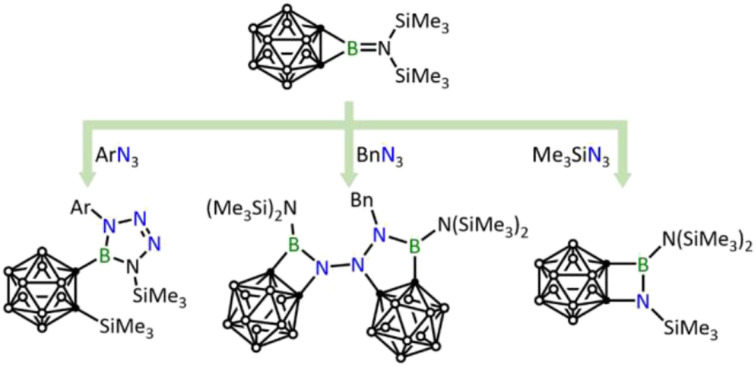

## Introduction

*Ortho*-dicarbadodecaboranyl-substituted boron compounds have attracted great attention in recent years. The unique ball-shaped structure of *o*-carborane, along with extensively delocalized skeletal electrons through multicenter bonding (referred to as 3D aromaticity), and its higher polarity compared to *m*-, *p*-carborane, make it an intriguing substituent. Notably, the strong inductive electron-withdrawing effect at the carbon positions has rendered it a useful substituent in constructing Lewis superacidic boranes.^1–5^ Moreover, *o*-carboranyl-substituted borenium has proven successful in the activation and conversion of methane.^6,7^*o*-Carboranyl-substituted iminoboranes exhibit distinct chemical properties compared to conventional iminoboranes.^8^ Furthermore, the *o*-carboranyl substituent demonstrates its capability in stabilizing reactive boron species such as oxoboranes.^9^ Additionally, owing to the unique hyperconjugation between carborane cluster and outer-cage groups,^10–12^ the 3D carborane cages hold substantial promise in replacing conventional 2D aromatic aryl groups for the construction of optoelectronic materials.^3,13–16^ However, despite these intriguing aspects, research on carboranyl boranes is still relatively lagging compared to traditional organoboranes.

On the other hand, borirenes^17–24^ and boriranes^8,9,24–35^ have gained attention due to their unique electronic and structural features.^36^ Typically, these small boracycles can undergo ring-opening reactions under irradiation^22^ or thermal conditions,^26–29^ or when reacting with polar bonds.^22,23,30,31^ However, it should be noted that their application in synthesis remains somewhat limited. Recently, a strategy to boost ring strain energy through the annelation of borirenes and boriranes has opened up possibilities for a range of new reactions. Most importantly, it has become evident that they can be applied as “C_2_B” synthons for organic and organometallic synthesis.^36^ It is noteworthy that, despite extensive research, their reactivity towards organic azides has not yet been reported.

**Fig. 1:**
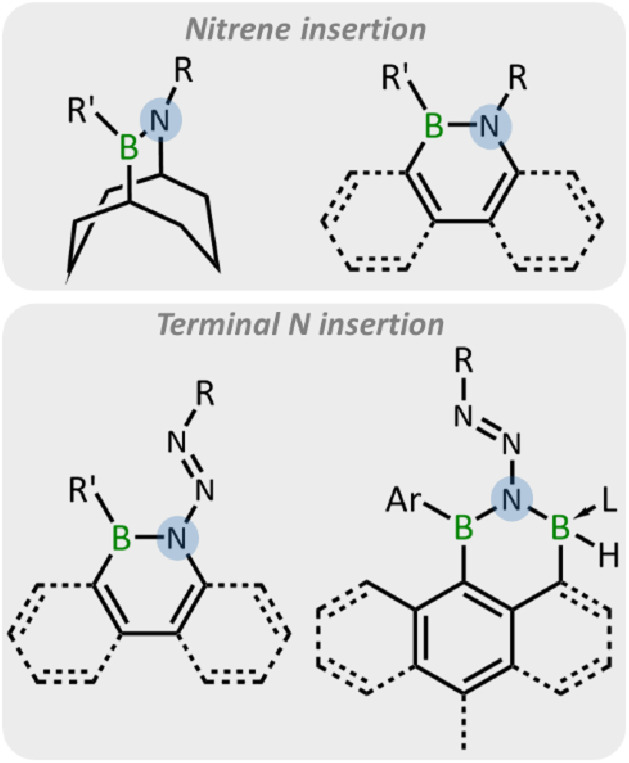
The reaction pattern of boracycles with azides.

Organic azides, since the first report in 1864,^37^ have attracted wide attention due to their energy-rich and variable reactivities.^38,39^ The nitrogen-rich N_3_ group exhibits diverse reactivity with electron-deficient boron compounds, including forming Lewis acid–base adducts,^40^ 1,3-dipolar cycloadditions with B=B/N bond,^41–45^ nitrene insertion into the B–B/C bond with concomitant loss of N_2_,^42,46–56^ and terminal nitrogen insertion into B–B/C bonds.^55–58^ Thus, organic azides are promising candidates for the construction of BN-containing compounds through their reactions with electron-deficient boron compounds.^59^ Among these reactions, the ring expansion reactions of boracycles with organic azides driven by aromaticity or release of ring strain have emerged as an effective strategy (Fig. 1).^49–58^ In 1987, the synthesis of 9,10-azaborabicycle was achieved *via* the reaction of 9-BBN with azides.^49,50^ More recently, the ring expansion of borole derivatives has been reported by the Braunschweig, Martin, and He groups, leading to the emergence of a wide range of BN-doped polycyclic aromatic hydrocarbons (PAHs).^51–58^

Based on the considerations mentioned above, we set out to utilize the *o*-carborane-fused aminoborirane 1 as a “C_2_B” synthon, aiming to achieve highly efficient synthesis of an array of carboranyl-substituted boron compounds through its reaction with organic azides. Herein, we report on three different reactivity patterns towards aryl, alkyl, and silyl azides. Indeed, this approach enables the isolation and full characterization of a series of novel *o*-carboranyl boron compounds.

## Results and discussion

**Scheme 1:**
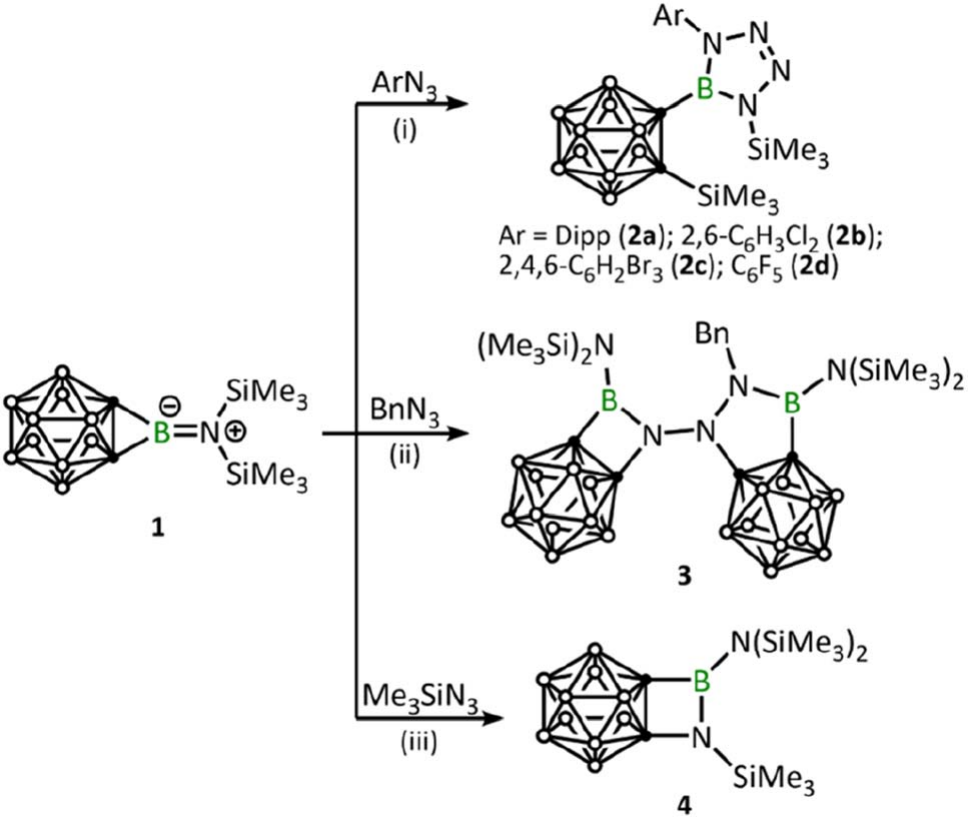
Reaction of 1 with ArN_3_, BnN_3_ and Me_3_SiN_3_. (i) 1.0 eq. ArN_3_, benzene, 60 °C to 100 °C, 4 h to 48 h; (ii) 0.5 eq. BnN_3_, toluene, room temperature, 0.5 h; (iii) 1.1 eq. Me_3_SiN_3_, toluene, 110 °C, 1 mbar, 12 h.

**Fig. 2:**
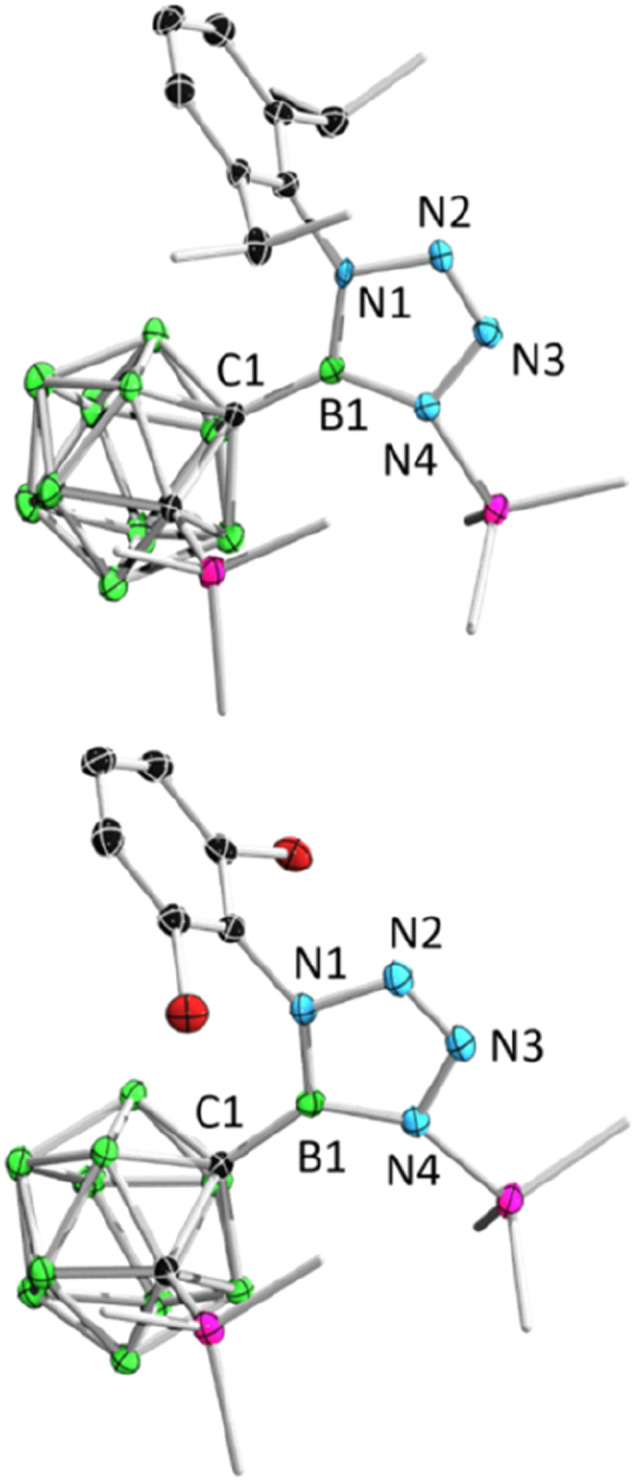
Solid-state structures of 2a (upper) and 2b (bottom). Hydrogen atoms have been removed for clarity.

After adding 1.0 equivalent of ArN_3_ (Ar = 2,6-iPr_2_C_6_H_4_, 2,6-C_6_H_3_Cl_2_, 2,4,6-C_6_H_2_Br_3_, C_6_F_5_) to borirane 1 in benzene at room temperature and waiting for half an hour (Scheme 1(i)), almost no reaction was observed as indicated by the ^11^B NMR spectrum. However, after heating (DippN_3_, 60 °C for 4 h; pentafluorophenyl azide, 100 °C for 24 hours; 2,6-dichloropenyl azide and 2,4,6-tribromophenyl azide, 100 °C for 48 hours), borirane 1 was completely consumed and new boron-containing species 2 (2a, *δ*_B_ 26.9; 2b–2d, *δ*_B_ 27.1) were observed in the ^11^B NMR spectrum. The ^1^H NMR spectra of 2 showed two doublets and two singlets with integrations of 3 : 3 : 9 : 9, indicating a loss of molecular symmetry. Colorless crystals were obtained with an isolated yield of 77–80% after work-up by slow evaporation of a saturated pentane solution of 2a and 2b at −35 °C in the glovebox refrigerator over a period of 24 hours. X-ray diffraction analysis of 2 revealed that the BC_2_ unit in 1 had undergone ring-opening accompanied by migration of the silyl group, as well as cycloaddition of ArN_3_ to the BN unit (Fig. 2). The central boron atom was found to be trigonal planar with a surrounded angle sum of 360° (2a, 359.9°; 2b, 360.0°). The geometry of the BN_4_ five-membered ring is comparable to that of reported tetrazaboroles,^43–45^ with a planar geometry and a sum of interior angles of 540° (2a, 540.1°; 2b, 539.9°). The N1–N2/N3–N4 distances were measured at 1.391(5)/1.409(5) Å (2a) and 1.3962(15)/1.4049(15) Å (2b), which fall within the range of typical N–N single bond distances and are significantly longer than the N2–N3 (2a, 1.271(5) Å; 2b, 1.2635(16) Å) double bonds. Besides, the IR spectra of compounds 2b–2d display an N=N stretching vibration in the range of 1465–1475 cm^−1^ (Fig. S32–S34†).

**Fig. 3:**
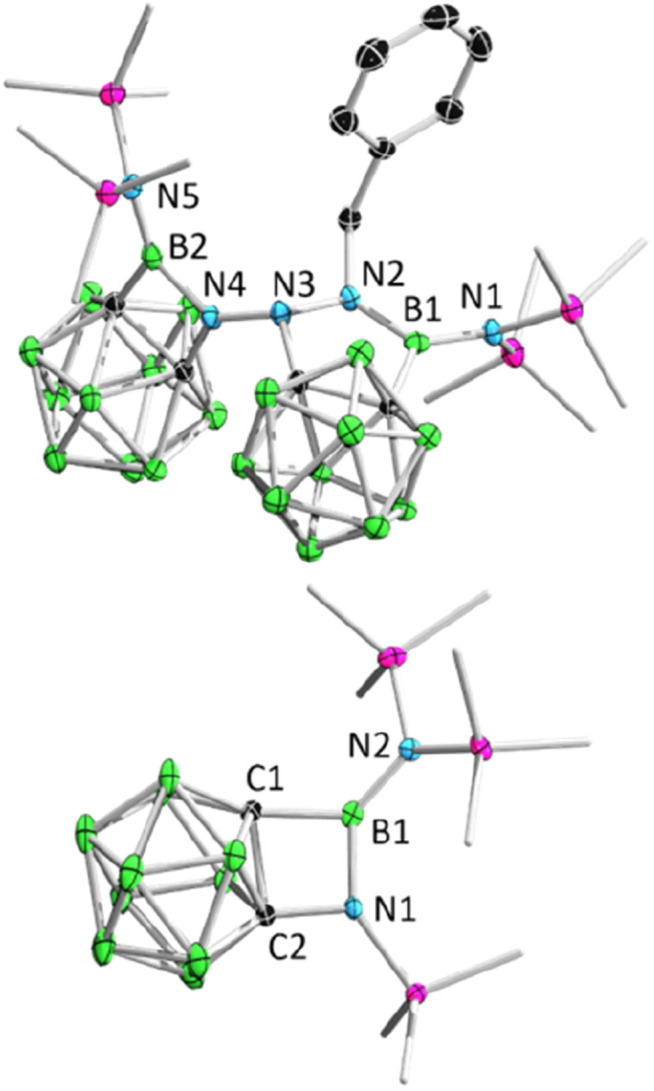
Solid-state structure of 3 (upper) and 4 (bottom). Hydrogen atoms have been removed for clarity.

Remarkably, BnN_3_ underwent a rapid ring expansion reaction with 2.0 equivalents of borirane 1 in toluene at room temperature (Scheme 1(ii)), as evidenced by a broad resonance at 34.7 ppm in the ^11^B NMR spectrum and the integration of one benzylic group and four SiMe_3_ groups in the ^1^H NMR spectrum. Colorless crystals of the newly formed product 3 were obtained in 66% yield by storing the reaction mixture in toluene at −35 °C for 24 hours. X-ray diffraction analysis revealed that the product 3, which was obtained in a 1 : 2 ratio of BnN_3_ : 1, features a BNC_2_ four-membered ring and a BN_2_C_2_ five-membered ring (Fig. 3). Attempts to isolate the 1 : 1 reaction product by slowly adding the toluene solution of 1 to the toluene solution of BnN_3_ at −20 °C were unsuccessful, as the NMR spectra still unambiguously indicated 3 as the major product.

Addition of 1.1 equivalents of Me_3_SiN_3_ to borirane 1 in C_6_D_6_ at room temperature did not result in any observable reaction. However, after heating the mixture at 80 °C for 48 hours, a set of new signals were observed in the ^11^B-NMR spectrum featuring a three-coordinate boron at 35.0 ppm and two Me_3_Si peaks on ^1^H-NMR in a 2 : 1 integration ratio at 0.13 and 0.01 ppm, respectively. The reaction rate was found to improve under reduced pressure (see ESI† for details), suggesting the release of N_2_ (Scheme 1(iii)). After an easy work-up, the product was obtained as colorless crystals in a 73% isolated yield by slow evaporation of a saturated pentane solution at −35 °C. X-ray diffraction analysis confirmed the liberation of one equivalent N_2_, indicating the formation of 4 featuring an *o*-carborane-fused planar BNCC four-membered ring with a sum of interior angles of 359.67° (Fig. 3). The endocyclic B1–N1 bond (1.456(2) Å) is slightly longer than the exocyclic B1–N2 bond (1.402(2) Å), which is attributable to the strain of the four membered ring.

Furthermore, reactions between 1 and some commonly used azide source reagents such as 2-azido-1,3-dimethylimidazolinium hexafluorophosphate and tosyl azide (also known as *p*-toluenesulfonyl azide) were examined. While no reaction with the former was observed, reaction with the latter turned out to be unselective, yielding a complex mixture.

**Fig. 4:**
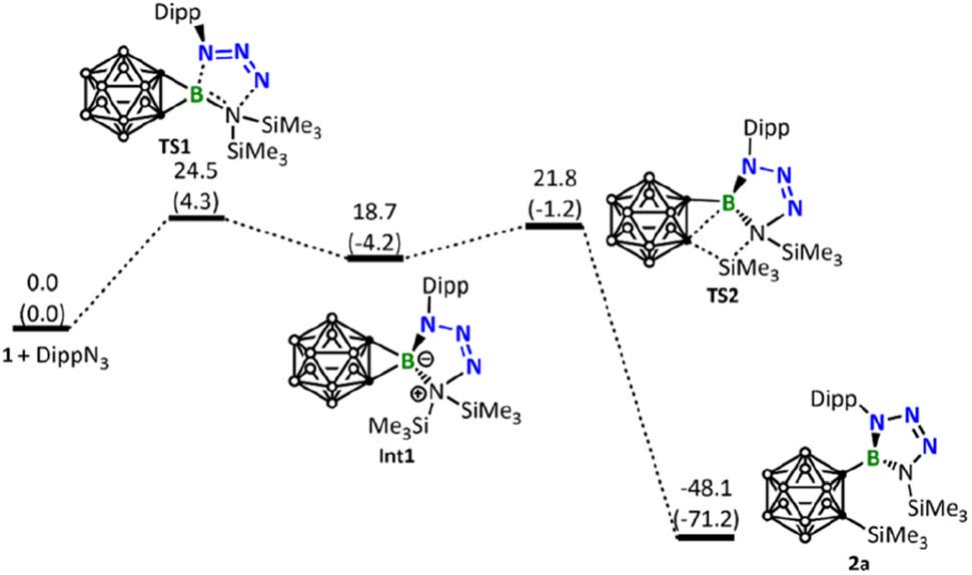
Energy profile calculated for the reaction of 1 with DippN_3_ leading to the formation of the experimentally observed product 2a. Relative free energies and electronic energies (in parentheses) are given in kcal mol^−1^.

DFT calculations were conducted to gain more insights into the reaction mechanism between borirane 1 and the different azides discussed above. Scheme 1 indicates that the reaction of 1 with ArN_3_ (Ar = Dipp, 2,6-C_6_H_3_Cl_2_, 2,4,6-C_6_H_2_Br_3_, or C_6_F_5_) proceeds *via* a [3 + 2] cycloaddition of the azide to the B=N moiety, followed by a silyl migration process and cleavage of one of the BC σ bonds. These steps lead to the formation of tetrazaborole 2. Our DFT calculations on the reaction of DippN_3_ corroborate well with this mechanism, indicating a step-wise process: cycloaddition followed by silyl migration and BC bond cleavage, with an overall energy barrier of 24.5 kcal mol^−1^ in the cycloaddition step (Fig. 4).

**Fig. 5:**
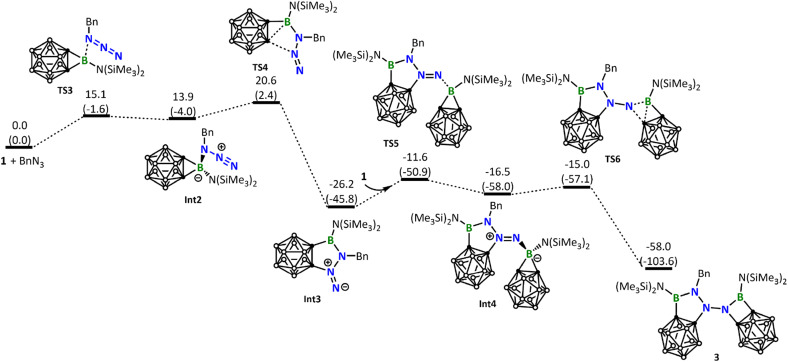
Energy profile calculated for the reaction of 1 with BnN_3_ leading to the formation of the experimentally observed product 3. Relative free energies and electronic energies (in parentheses) are given in kcal mol^−1^.

Interestingly, the reaction of 1 with BnN_3_ clearly follows a pathway distinctly different from the reaction with DippN_3_. DFT calculations show that the Lewis acid–base 1-BnN_3_ adduct (Int2) was firstly formed. Due to the electron releasing property of the benzylic group, coordination of BnN_3_ to the boron center of 1 is significantly stronger than that of DippN_3_, and therefore the B–C σ bonds in Int2 are significantly weakened (see the comparison of the B–C and B–N distances among RN_3_-1 adducts in Fig. S39†). As a result of the B–C σ bond weakening, in the next step from Int2 to Int3, we see a B–C σ bond cleavage accompanied by a C–N bond formation with the central N atom of the azide unit (Fig. 5). We can conveniently assume that the C–N bond formation is a nucleophilic attack of C on N. The nucleophilic attack occurs on the central N instead of the terminal N of azide, clearly due to a reason related to the geometric requirement. Fig. 5 shows that the formation of the intermediate Int3 requires a barrier of 20.6 kcal mol^−1^. Fig. 5 also shows that Int3 is highly reactive toward another molecule of borirane 1, coordination followed by ring expansion leading to the formation of the experimentally observed product 3, with a barrier of 14.6 kcal mol^−1^ in the coordination step. It is noteworthy that the barrier for Int3 reacting with the second molecule of 1 (14.6 kcal mol^−1^) is even lower than that for BnN_3_ reacting with the first molecule of 1 (20.6 kcal mol^−1^). This result indicates that the reaction of Int3 with 1 is much faster than the reaction of BnN_3_ with 1, explaining why the attempt to obtain the 1 : BnN_3_ 1 : 1 product results in failure.

Up to this point, readers may wonder why DippN_3_ (Fig. 4) did not follow the same reaction path leading to C–B bond cleavage/C–N bond formation as BnN_3_ did (Fig. 5). To address this issue, we calculated the same pathway for DippN_3_ leading to Int3-Dipp (Fig. S35†) and found the corresponding C–B bond cleavage/C–N bond formation transition state TS4-Dipp lying at 28.9 kcal mol^−1^ above the reactants (1 + DippN_3_), which is much less favorable than the favorable [3 + 2] cycloaddition (with a barrier of 24.5 kcal mol^−1^) presented in Fig. 4. Compared with BnN_3_, where Bn is highly electron-releasing, DippN_3_, where Dipp is bulkier and has strong conjugation capability, is expected to have much electron-poorer azide unit and show much weaker coordination ability to the 3-coordinated boron center of borirane 1, as evidenced by the much longer coordination bond (see Fig. S39† comparing the structures of Int2 and Int2-Dipp). Clearly, the much weaker coordination ability of DippN_3_ contributes to the high lying TS4-Dipp. Building upon this idea, it's clear that the considerably more electron-deficient azides bearing 2,6-dichlorophenyl, 2,4,6-tribromophenyl, and pentafluorophenyl substituents followed a similar reaction pattern with DippN_3_. Moreover, they demanded even more rigorous reaction conditions than DippN_3_.

**Fig. 6:**
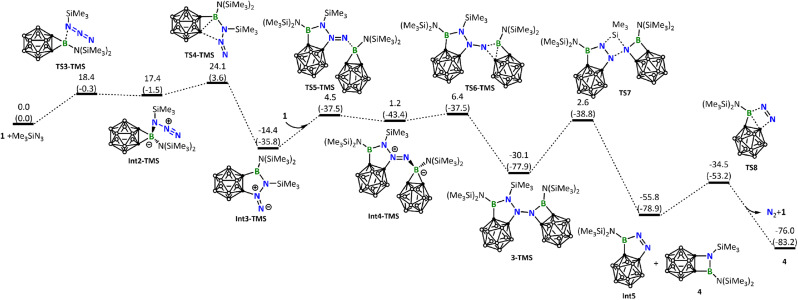
Energy profile calculated for the reaction of 1 with Me_3_SiN_3_ leading to the formation of the experimentally observed product 4. Relative free energies and electronic energies (in parentheses) are given in kcal mol^−1^.

Next, we calculated the reaction of 1 with Me_3_SiN_3_. Like BnN_3_, Me_3_SiN_3_ also possesses an electron-rich azide unit and, as expected, follows a similar reaction pathway leading to the five-membered ring intermediate Int3-TMS (Fig. 6). The barrier (24.1 kcal mol^−1^, Fig. 6) leading to Int3-TMS is higher than the corresponding one (20.6 kcal mol^−1^, Fig. 5) calculated for BnN_3_, a result of steric effect due to the much bulkier TMS when compared with Bn. Again, similar to what we have seen for the reaction of BnN_3_, Int3-TMS is also very reactive toward another molecule of 1, resulting in the formation of 3-TMS.

Unlike the experimentally observed product 3 from the reaction of BnN_3_, here, the corresponding species 3-TMS undergoes further structural rearrangement *via* silyl migration, leading to an N–N bond cleavage to give Int5 and the final product 4. An extrusion of N_2_ from Int5 regenerates borirane 1. The silyl migration step is rate-determining in this reaction, with a barrier of 32.7 kcal mol^−1^, consistent with the experimental observation that harsh reaction conditions are necessary. For readers' information, we also calculated other possible reaction pathways for the reaction of 1 with Me_3_SiN_3_, all of which have higher energy barriers (see Fig. S37†).

Based on this information, we are curious whether compound 3 can follow a reaction pathway similar to that of 3-TMS and be further transformed into an analogue of 4. At 110 °C, compound 3 indeed undergoes further conversion within a few hours, affording a new boron-containing species with an ^11^B resonance at 31.8 ppm, closely resembling that of 4 (*δ*_B_ 34.9). However, the regeneration of 1 was not observed. Due to the oily nature of the product mixture, attempts to isolate the product through crystallization were unsuccessful. The differences in the further transformation of 3 compared to 3-TMS may be attributed to the relatively higher energy barrier for the migration of the Bn group in 3, and the presence of reactive H on the benzyl group that enables other possible reaction pathways.

## Conclusions

In summary, we investigated the reactivity of carborane-fused aminoborirane towards three different types of organic azides. An array of novel carboranyl-substituted boron compounds have been successfully synthesized and fully characterized. Furthermore, thanks to the detailed computational studies, we have suggested reaction mechanisms distinct from those proposed in previous reactions between boracycles and organic azides: the aryl substituent has strong conjugation capability, which makes the azide group electron-poor. As a result, the coordination between the azide group and borirane is weak, and thus the endocyclic B–C bond of borirane is not sufficiently weakened. Indeed, this leads to [3 + 2] cycloaddition of the azido group with the exocyclic B=N bond, followed by the simultaneous ring-opening and silyl migration, affording the first carboranyl 4,5-dihydrotetraazaboroles. Conversely, the benzyl and silyl azides possess both a relatively electron-rich azide group. Consequently, the endocyclic BC bond of borirane is effectively weakened upon the nucleophilic attack of the α-N of the azido group, which facilitates the insertion of N(α)N(β) – a novel reactivity pattern between the boracycle and azide. Correspondingly, the γ-N is converted to a reactive nitrene species, allowing it to readily insert into the second equivalent borirane, leading to the NN-linked diazaborole-azaborete compounds that have not been reported previously. While the diazaborole-azaborete product derived from the reaction with benzyl azide can be isolated and fully characterized, the reaction with silyl azide ultimately leads a carborane-fused azaborete, which is attributed to the ease of silyl migration and cleavage of the bridging NN-bond. After the NN-cleavage and departure of the silyl group, the other half of the molecule, *i.e.* a free carborane-fused diazaborole, merely needs to overcome an energy barrier of 21.3 kcal mol^−1^ to liberate N_2_ with borirane being regenerated. Combined, the reactions involving azides and highly strained carborane-fused borirane exhibt a clear distinctiveness, and the findings presented herein clearly demonstrate that the high strain introduces a new and intriguing dimension to the reaction chemistry, and thus deserving ongoing efforts.

## Data availability

All experimental and computational data are available in ESI.†

## Author contributions

J. W., L. X. and X. L. carried out the experiments. Z. L. supervised the computational studies. J. W. performed the DFT calculations. A. M. performed single crystal X-ray diffraction analysis. Q. Y. conceived and supervised the project. All authors discussed the results and contributed to the final manuscript.

## Conflicts of interest

There are no conflicts to declare.

## Supplementary Material

SC-015-D4SC00489B-s001

SC-015-D4SC00489B-s002
