# Supplementary material for: Avenue to novel o-carboranyl boron compounds – reactivity study of o-carborane-fused aminoborirane towards organic azides
Source: Chem Sci. 2024 Feb 22;15(13):4839–45. doi: 10.1039/d4sc00489b (PMC10966985; doi:10.1039/d4sc00489b)
Supplement: SC-015-D4SC00489B-s001 [file SC-015-D4SC00489B-s001.pdf]

Electronic Supplementary Material (ESI) for Chemical Science.
This journal is © The Royal Society of Chemistry 2024

Electronic Supplementary Information

Avenue to Novel *o*-Carboranyl Boron Compounds – Reactivity Study of o-Carborane-Fused Aminoborirane towards Organic Azides

Table of Contents

Experimental Procedures 2
Crystal structures...22
Computational details 25
Cartesian coordinates..28
References 49

Experimental Procedures

General considerations. All manipulations were conducted either under an atmosphere of dry argon or in *vacuo* using standard Schlenk line or glovebox techniques. Solvents were purified by distillation from Na/K under dry argon. C_6D_6 was degassed by three freeze-pump-thaw cycles and stored over molecular sieves. Borirane **1** were prepared according to published procedures.[1] Note: Starting material **1** is sensitive to volatile ether solvents such as THF and Et_2O.[2] The presence of catalytic amounts of ether in the solvent or atmosphere during the synthesis of **2**-**4** may result in the formation of by-products.

NMR spectra were acquired on a Bruker *Avance 400* (^{1}H: 400.1 MHz, ^{11}B: 128.4 MHz, ^{13}C: 100.6 MHz) NMR spectrometer at 298 K without mentioned. ^{1}H, ^{13}C{^{1}H} and ^{1}H{^{11}B} spectra were referenced to external TMS. ^{11}B and ^{11}B{^{1}H} NMR spectra were referenced to external $BF_3{\cdot}OEt_2$. High resolution mass spectrometry (HRMS) was performed with a Thermo Fisher Scientific Q-Exactive MS System. Elemental analysis (C, H, N) was performed on a vario micro cube CHNS analyzer.

Synthetic protocols for 2

Synthesis of 2a

DippN$_3$ (65 mg, 0.32 mmol, 1.0 eq.) was added to **1** (100 mg, 0.32 mmol) in 2 mL benzene at room temperature. After heating to 60 °C for 4 hours, the reaction system was cooled to room temperature and all volatiles were removed under vacuum to obtain a colorless solid. Analytically pure product (132 mg, 0.26 mmol, 80%) was isolated as colorless block crystals by slowly evaporating a saturated pentane solution of **2a** at −35°C in the refrigerator of glovebox for 24 hours.

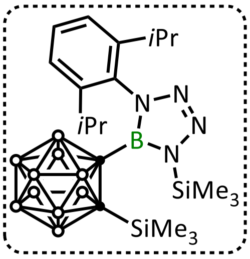

^{1}H NMR (C_6D_6): δ = 7.25 (*t*, *J* = 7.8 Hz, 1H, H of Ph), 7.11 (*dd*, *J* = 7.8, 1.4 Hz, 1H, H of Ph), 7.03 (*dd*, *J* = 7.7, 1.4 Hz, 1H, H of Ph), 3.81 to 2.26 (*m*, 10H, B*H*), 3.00 (*hept*, *J* = 6.8 Hz, 1H, C*H*(CH_3)$_2$), 2.04 (*hept*, *J* = 6.8 Hz, 1H, C*H*(CH_3)$_2$), 1.23 (*d*, *J* = 6.9 Hz, 3H, CH(C*H*$_3$)$_2$), 1.19 (*d*, *J* = 6.9 Hz, 3H, CH(C*H*$_3$)$_2$), 1.18 (*d*, *J* = 6.8 Hz, 3H, CH(C*H*$_3$)$_2$), 1.01 (*d*, *J* = 6.8 Hz, 3H,CH(C*H*$_3$)$_2$), 0.49 (*s*, 9H, Si*Me*$_3$), –0.04 (*s*, 9H, Si*Me*$_3$); **^{1}H{^{11}B} NMR** (C_6D_6): δ = 7.23 (*t*, *J* = 7.8 Hz, 1H, H of Ph), 7.11 (*d*, *J* = 7.7, 1H, H of Ph), 7.04 (*d*, *J* = 7.7, 1H, H of Ph), 3.15 (*s*, 1H, B*H*), 3.08 (*s*, 2H, B*H*), 3.01 (*hept*, *J* = 6.9 Hz, 1H, C*H*(CH_3)$_2$), 2.86 (*s*, 1H, B*H*), 2.83 (*s*, 1H, B*H*), 2.56 (*s*, 1H, B*H*), 2.46 (*s*, 1H, B*H*), 2.37 (*s*, 1H, B*H*), 2.27 (*s*, 1H, B*H*), 2.15 (*s*, 1H, B*H*), 2.04 (*hept*, *J* = 6.8 Hz, 1H, C*H*(CH_3)$_2$), 1.23 (*d*, *J* = 6.9 Hz, 3H, CH(C*H*$_3$)$_2$), 1.19 (*d*, *J* = 6.9 Hz, 3H, CH(C*H*$_3$)$_2$), 1.18 (*d*, *J* = 6.9 Hz, 3H, CH(C*H*$_3$)$_2$), 1.01 (*d*, *J* = 6.9 Hz, 3H,CH(C*H*$_3$)$_2$), 0.50 (*s*, 9H, Si*Me*$_3$), –0.03 (*s*, 9H, Si*Me*$_3$); **^{11}B NMR** (C_6D_6): δ = 26.9 (*s*, Cb*B*N$_4$), 1.8 (*d*, *J* = 140.9 Hz, *B*$_{Cb}$), –3.8 (*d*, *J* = 106.1 Hz, *B*$_{Cb}$), –9.2 to –12.1 (*m*, *B*$_{Cb}$); **^{11}B{^{1}H} NMR** (C_6D_6): δ = 27.0 (*s*, Cb*B*N$_4$), 1.8 (*s*, *B*$_{Cb}$), –3.9 (*s*, *B*$_{Cb}$), –8.7 (*s*, *B*$_{Cb}$), –11.8 (*s*, *B*$_{Cb}$); **^{13}C{^{1}H} NMR** (C_6D_6): δ = 148.1 (C of Ph), 145.4 (C of Ph), 135.0 (C of Ph), 130.4 (C of Ph), 124.3 (C of Ph), 123.6 (C of Ph), 76.5 (*C*SiMe$_3$), 29.7 (*C*H(CH_3)$_2$), 29.6 (*C*H(CH_3)$_2$), 26.2 (CH(*C*H$_3$)$_2$), 25.6 (CH(*C*H$_3$)$_2$), 21.4 (CH(*C*H$_3$)$_2$), 2.7 (Si*Me*$_3$), 0.4 (Si*Me*$_3$); **HRMS (m/z)**: [M+H]$^+$ calcd. for $C_{20}H_{46}N_4B_{11}Si_2$, 519.42791; found 519.42828; **Elemental analysis**: calcd. for $C_{20}H_{45}B_{11}N_4Si_2$, C, 46.49; H, 8.78; N, 10.84; found C, 46.59; H, 8.84; N, 10.86.

Synthesis of 2b

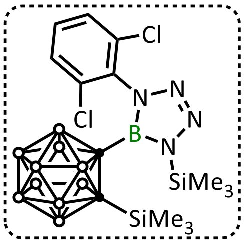

2,6-Dichlorphenylazide (56 mg, 0.30 mmol, 1.0 eq.) was added to **1** (94 mg, 0.30 mmol) in 2 mL benzene at room temperature. After heating to 100 °C for 2 days, the reaction system was cooled to room temperature and all volatiles were removed under vacuum to obtain a yellow solid. Analytically pure product (115 mg, 0.23 mmol, 77%) was isolated as yellow block crystals by slowly evaporating a saturated pentane solution of **2b** at −35°C in the refrigerator of glovebox for 24 hours.

^{1}H NMR (C_6D_6): δ = 6.82 (*dd*, *J* = 8.2, 1.3 Hz, 1H, H of Ph), 6.73 (*dd*, *J* = 8.2, 1.3 Hz, 1H, H of Ph), 6.37 (*t*, *J* = 8.1 Hz, 1H, H of Ph), 3.15 to 2.37 (*m*, 10H, B*H*), 0.48 (*s*, 9H, Si*Me$_3$*), 0.00 (*s*, 9H, Si*Me$_3$*); **^{1}H{^{11}B} NMR** (C_6D_6): δ = 6.82 (d*d*, *J* = 8.2, 1.3 Hz, 1H, H of Ph), 6.73 (*dd*, *J* = 8.2, 1.3 Hz, 1H, H of Ph), 6.37 (*t*, *J* = 8.1 Hz, 1H, H of Ph), 3.15 (*s*, 3H, B*H*), 2.91 (*s*, 1H, B*H*), 2.78 (*s*, 1H, B*H*), 2.67 (*s*, 1H, B*H*), 2.48 (*s*, 2H, B*H*), 2.37 (*s*, 2H, B*H*), 0.48 (*s*, 9H, Si*Me$_3$*), 0.00 (*s*, 9H, Si*Me$_3$*); **^{11}B NMR** (C_6D_6): δ = 27.1 (*s*, Cb*B*N$_4$), 1.8 (*d*, *J* = 146.9 Hz, *B*$_{Cb}$), –3.9 (*d*, *J* = 136.4 Hz, *B*$_{Cb}$), –8.9 to –12.1 (*m*, *B*$_{Cb}$); **^{11}B{^{1}H} NMR** (C_6D_6): δ = 27.2 (*s*, Cb*B*N$_4$), 1.9 (*s*, *B*$_{Cb}$), –3.9 (*s*, *B*$_{Cb}$), –8.6 (*s*, *B*$_{Cb}$), –11.7 (*s*, *B*$_{Cb}$); **^{13}C{^{1}H} NMR** (C_6D_6): δ = 135.0 (C of Ph), 132.5 (C of Ph), 132.3 (C of Ph), 128.6 (C of Ph), 126.7 (C of Ph), 126.6 (C of Ph), 74.5 (*C*SiMe$_3$), 0.2 (Si*Me$_3$*), –2.0 (Si*Me$_3$*); **HRMS (LIFDI)**: calcd. for $C_{14}H_{31}B_{11}Cl_2N_4Si_2$: *m/z* = 500.2561; found: *m/z* = 500.2555.

Synthesis of 2c

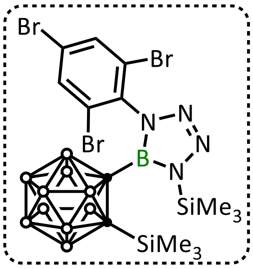

2,4,6-trisbromophenylazid (110 mg, 0.30 mmol, 1.0 eq.) was added to **1** (94 mg, 0.30 mmol) in 2 mL benzene at room temperature. After heating to 100 °C for 2 days, the reaction system was cooled to room temperature and all volatiles were removed under vacuum to obtain **2c** as a beige oil (127 mg, 0.19 mmol, 63%).

^{1}H NMR (C_6D_6): δ = 7.24 (*d*, *J* = 2.1 Hz, 1H, H of Ph), 7.14 (*d*, *J* = 2.1 Hz, 1H, H of Ph), 3.15 to 2.34 (*m*, 10H, B*H*), 0.49 (*s*, 9H, Si*Me$_3$*), 0.00 (*s*, 9H, Si*Me$_3$*); **^{1}H{^{11}B} NMR** (C_6D_6): δ = 7.25 (*d*, *J* = 2.0 Hz, 1H, H of Ph), 7.15 (*d*, *J* = 2.0 Hz, 1H, H of Ph), 3.15 (*s*, 3H, B*H*), 2.88 (*s*, 1H, B*H*), 2.77 (*s*, 1H, B*H*), 2.61 (*s*, 1H, B*H*), 2.47 (*s*, 1H, B*H*), 2.38 (*s*, 1H, B*H*), 2.34 (*s*, 2H, B*H*), 0.49 (*s*, 9H, Si*Me$_3$*), 0.09 (*s*, 9H, Si*Me$_3$*); **^{11}B NMR** (C_6D_6): δ = 27.1 (*s*, Cb*B*N$_4$), 1.9 (*d*, *J* = 139.3 Hz, *B*$_{Cb}$), –3.8 (*d*, *J* = 117.8 Hz, *B*$_{Cb}$), –8.6 to –11.4 (*m*, *B*$_{Cb}$); **^{11}B{^{1}H} NMR** (C_6D_6): δ = 26.7 (*s*, Cb*B*N$_4$), 1.9 (*s*, *B*$_{Cb}$), –3.7 (*s*, *B*$_{Cb}$), –8.7 (*s*, *B*$_{Cb}$), –11.7 (*s*, *B*$_{Cb}$); **^{13}C{^{1}H} NMR** (C_6D_6): δ = 134.2 (*C* of Ph), 133.2 (*C* of Ph), 133.0 (*C* of Ph), 126.5 (*C* of Ph), 122.4 (*C* of Ph), 122.1 (*C* of Ph), 74.6 (*C*SiMe$_3$), 0.2 (Si*Me$_3$*), –1.7 (Si*Me$_3$*); **HRMS (LIFDI)**: calcd. for $C_{14}H_{30}B_{11}Br_3N_4Si_2$: *m/z* = 669.0661; found: *m/z* = 669.0606.

Synthesis of 2d

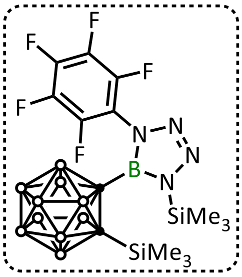

Pentafluorophenylazide (68 mg, 0.3 mmol, 1.0 eq.) was added to **1** (94 mg, 0.3 mmol) in 2 mL benzene at room temperature. After heating to 100 °C for 24 hours, the reaction system was cooled to room temperature and all volatiles were removed under vacuum to obtain a yellowish brown oil of **2d** (83 mg, 0.16 mmol, 54%).

^{1}H NMR (C_6D_6): δ = 3.14 to 2.07 (*m*, 10H, B*H*), 0.38 (*s*, 9H, Si*Me$_3$*), –0.14 (*s*, 9H, Si*Me$_3$*); **^{1}H{^{11}B} NMR** (C_6D_6): δ = 3.15 (*s*, 1H, B*H*), 3.11 (*s*, 2H, B*H*), 2.96 (*s*, 2H, B*H*), 2.64 (*s*, 1H,

B*H*), 2.58 (*s*, 1H, B*H*), 2.44 (*s*, 1H, B*H*), 2.35 (*s*, 1H, B*H*), 2.30 (*s*, 1H, B*H*), 2.07 (*s*, 1H, B*H*), 0.39 (*s*, 9H, Si*Me₃*), –0.14 (*s*, 9H, Si*Me₃*); **^{11}B NMR** (C_6D_6): δ = 27.1 (*s*, Cb*B*N_4), 2.0 (*d*, *J* = 140.9 Hz, B_{Cb}), –3.9 (*d*, *J* = 106.1 Hz, B_{Cb}), –8.52 to –11.6 (*m*, B_{Cb}); **^{11}B{^{1}H} NMR** (C_6D_6): δ = 27.1 (*s*, Cb*B*N_4), 2.0 (*s*, B_{Cb}), –2.1 (*s*, B_{Cb}), –4.0 (*s*, B_{Cb}), –9.1 (*s*, B_{Cb}), –12.2 (*s*, B_{Cb}); **^{13}C{^{1}H} NMR** (C_6D_6): δ = 129.3 (*C* of Ph), 128.6 (*C* of Ph), 127.5 (*C* of Ph), 125.7 (*C* of Ph), 76.1 (*C*SiMe₃), 2.2 (Si*Me₃*), –0.6 (Si*Me₃*); **^{19}F NMR** (C_6D_6): δ = –143.0, –143.7, –149.6, –160.1, –161.5; **HRMS (LIFDI)**: calcd. for $C_{14}H_{28}B_{11}F_5N_4Si_2$: *m/z* = 522.2869; found: *m/z* = 522.2863.

Synthesis of 3

BnN_3 (21 mg, 0.16 mmol, 0.5 eq.) in 1 mL toluene was added into **1** (100 mg, 0.32 mmol) in 1 mL toluene at room temperature. After mixing thoroughly, the reaction system was stored at –35 °C for 24 h. Analytically pure product (80 mg, 0.11 mmol, 66 %) was isolated as colourless crystals.

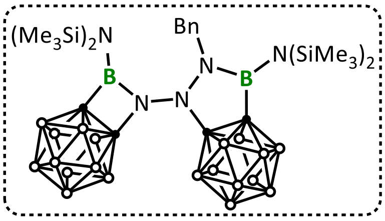

^{1}H NMR (toluene-d8): δ = 7.25 to 7.23 (*m*, 2H, H of Ph), 7.06 to 7.01 (*m*, 3H, H of Ph), 4.26 (*dd*, *J* = 36.5, 16.3 Hz, 2H, C*H₂*), 3.64 to 2.21 (*m*, 20H, B*H*), 0.26 (*s*, 9H, Si*Me₃*), 0.13 (*s*, 18H, Si*Me₃*), –0.15 (*s*, 9H, Si*Me₃*); **^{1}H{^{11}B} NMR** (toluene-d8): δ = 7.25 to 7.23 (*m*, 2H, H of Ph), 7.06 to 6.99 (*m*, 3H, H of Ph), 4.27 (*dd*, *J* = 36.3, 16.3 Hz, 2H, C*H₂*), 3.55 (*s*, 1H, B*H*), 3.45 (*s*, 1H, B*H*), 3.21 (*s*, 1H, B*H*), 3.15 (*s*, 1H, B*H*), 3.04 (*s*, 1H, B*H*), 2.92 (*s*, 2H, B*H*), 2.82 to 2.67 (*m*, 8H, B*H*), 2.60 (*s*, 1H, B*H*), 2.50 (*s*, 1H, B*H*), 2.39 (*s*, 2H, B*H*), 2.26 (*s*, 1H, B*H*), 0.26 (*s*, 9H, Si*Me₃*), 0.13 (*s*, 18H, Si*Me₃*), –0.15 (*s*, 9H, Si*Me₃*); **^{11}B NMR** (toluene-d8): δ = 34.7 (*br*, Cb*B*N), 2.9 to –11.4 (*m*, B_{Cb}); **^{11}B{^{1}H} NMR** (toluene-d8): δ = 34.7 (*br*, Cb*B*N), 2.5 (*s*, B_{Cb}), –2.6(*s*, B_{Cb}), –4.4 (*s*, B_{Cb}), –8.8 (*s*, B_{Cb}), –11.3 (*s*, B_{Cb}); **^{13}C{^{1}H} NMR** (toluene-d8): δ = 138.0 (C of Ph), 137.5 (C of Ph), 128.7 (C of Ph), 128.4 (C of Ph), 91.1 (C_{Cb}N), 90.7 (C_{Cb}N), 50.2 (CH_2), 4.6 (Si*Me₃*), 4.2 (Si*Me₃*), 3.7 (Si*Me₃*); **HRMS (m/z)**: $[M+H]^+$ calcd. for $C_{23}H_{64}N_5B_{22}Si_4$, 764.62805; found 764.63464; **Elemental analysis**: calcd. for $C_{23}H_{63}B_{22}N_5Si_4$, C, 36.35; H, 8.36; N, 9.22; found C,36.50; H,8.43; N, 9.07.

Synthesis of 4

Me_3SiN_3 (40 mg, 0.35 mmol, 1.1 eq) was added to **1** (100 mg, 0.32 mmol) in 2 mL toluene at room temperature. The reaction system was heated to 110 °C under reduced pressure. After 12 hours, the reaction system was cooled to room temperature and all volatiles were removed under vacuum to obtain a colorless solid. Analytically pure product (94 mg, 0.24 mmol, 73%) was isolated as colorless block crystals by slowly evaporating a saturated pentane solution of **4** at −35°C in the refrigerator of glovebox for 48 hours.

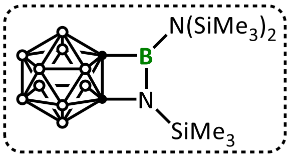

^{1}H NMR (C_6D_6): δ = 3.74 to 2.15 (*m*, 10H, B*H*), 0.13 (*s*, 18H, Si*Me₃*), 0.01 (*s*, 9H, Si*Me₃*); **^{1}H{^{11}B} NMR** (C_6D_6): δ = 3.27 (*s*, 2H, B*H*), 3.16 (*s*, 1H, B*H*), 2.85 (*s*, 2H, B*H*), 2.73 (*m*, 3H, B*H*), 2.61 (*s*, 2H, B*H*), 0.13 (*s*, 18H, Si*Me₃*), 0.02 (*s*, 9H, Si*Me₃*); **^{11}B NMR** (C_6D_6): δ = 35.0 (*s*, Cb*B*N), 1.94 (*d*, *J* = 154.5 Hz, B_{Cb}), 0.0 (*d*, *J* = 175.5 Hz, B_{Cb}), –8.3 (*m*, B_{Cb}), –11.2 (*d*, *J* = 160.7 Hz, B_{Cb}), –13.9 (*d*, *J* = 162.4 Hz, B_{Cb}); **^{11}B{^{1}H} NMR** (C_6D_6): δ = 34.9 (*s*, Cb*B*N), 2.0 (*s*, B_{Cb}), 0.0 (*s*, B_{Cb}), –8.3 (*s*, B_{Cb}), –11.2 (*s*, B_{Cb}), –13.8 (*s*, B_{Cb}); **^{13}C{^{1}H} NMR** (C_6D_6): δ = 89.0 (C_{Cb}N), 3.4 (Si*Me₃*), 0.01 (Si*Me₃*); **HRMS (m/z)**: $[M+H]^+$ calcd. for $C_{11}H_{38}B_{11}N_2Si_3$, 403.33664; found 403.33646; **Elemental analysis**: calcd. for $C_{11}H_{37}B_{11}N_2Si_3$, C, 32.98; H, 9.31; N, 6.99; found C, 32.74; H,9.22; N, 7.08.

NMR and IR spectra

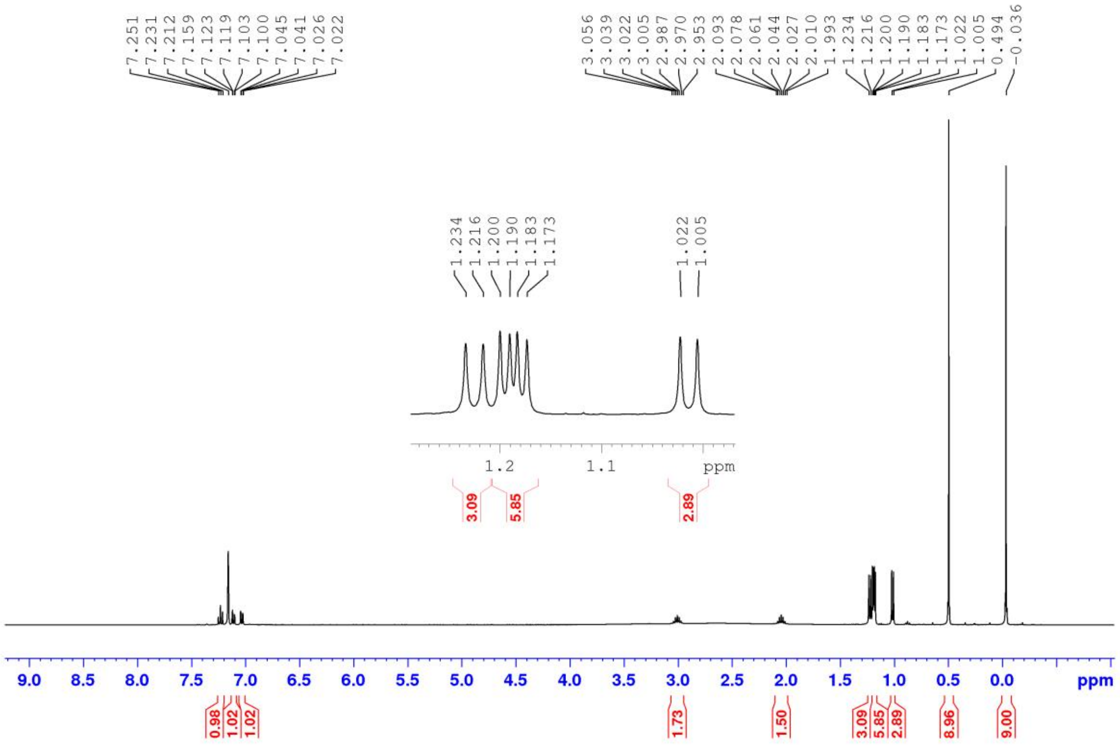

Figure S1. ^{1}H NMR spectrum of **2a** in C_6D_6.

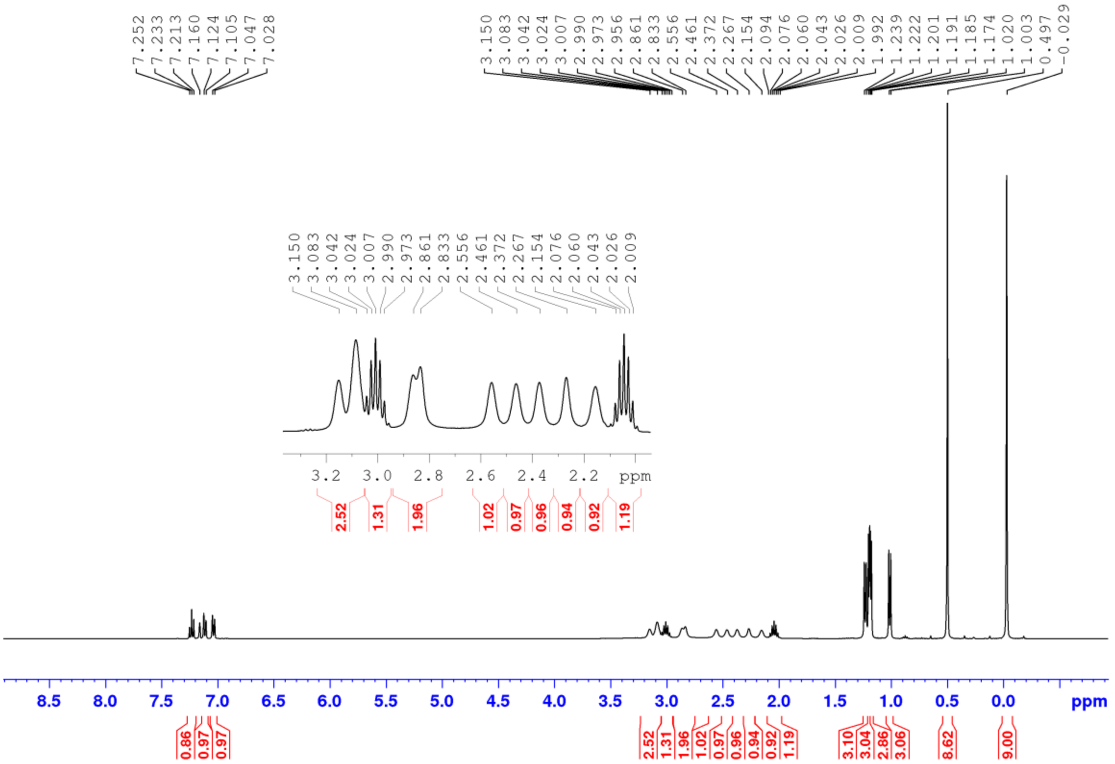

Figure S2. ^{1}H{^{11}B} NMR spectrum of **2a** in C_6D_6.

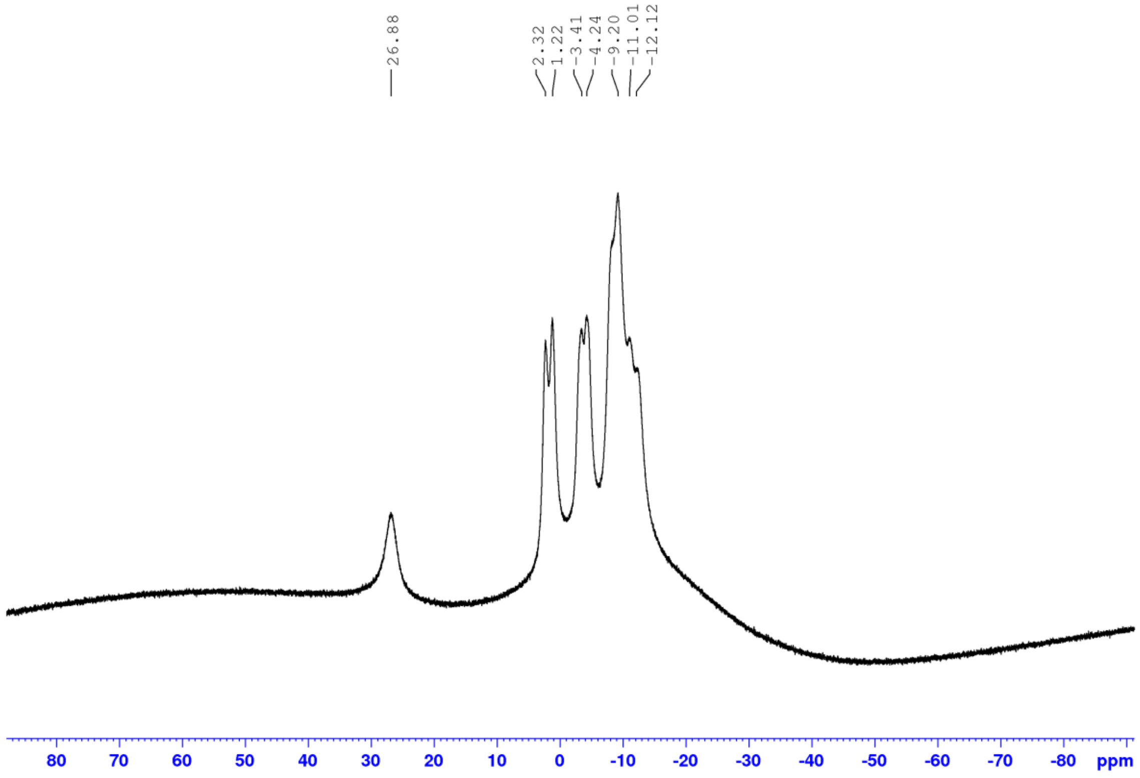

Figure S3. ^{11}B NMR spectrum of **2a** in C_6D_6.

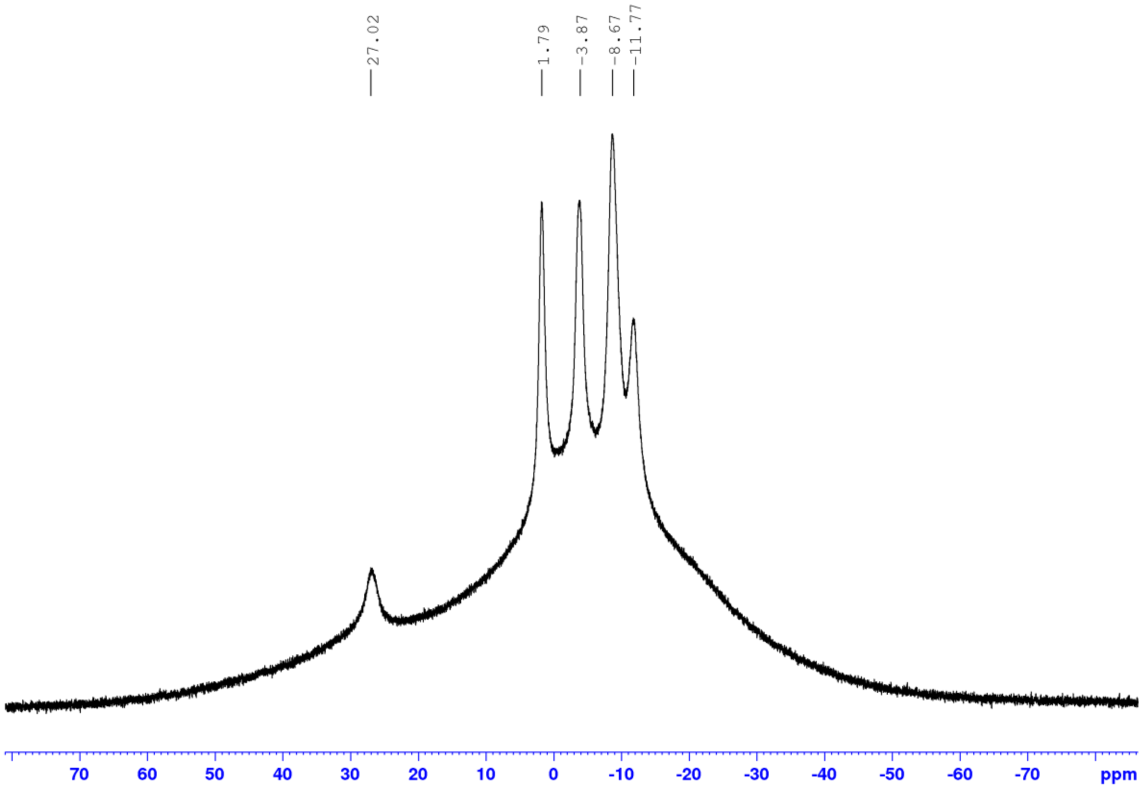

Figure S4. $^{11}B\{^1H\}$ NMR spectrum of **2a** in C_6D_6.

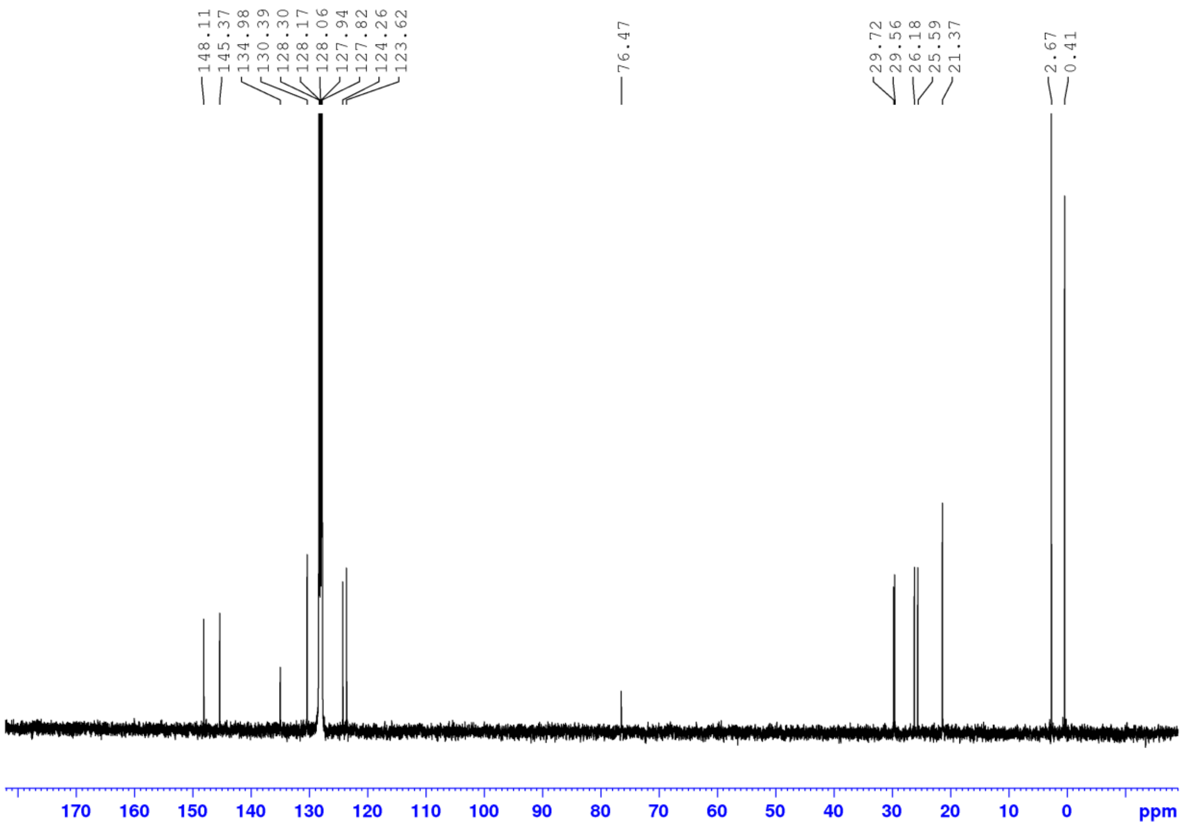

Figure S5. $^{13}C\{^{1}H\}$ NMR spectrum of **2a** in C_6D_6.

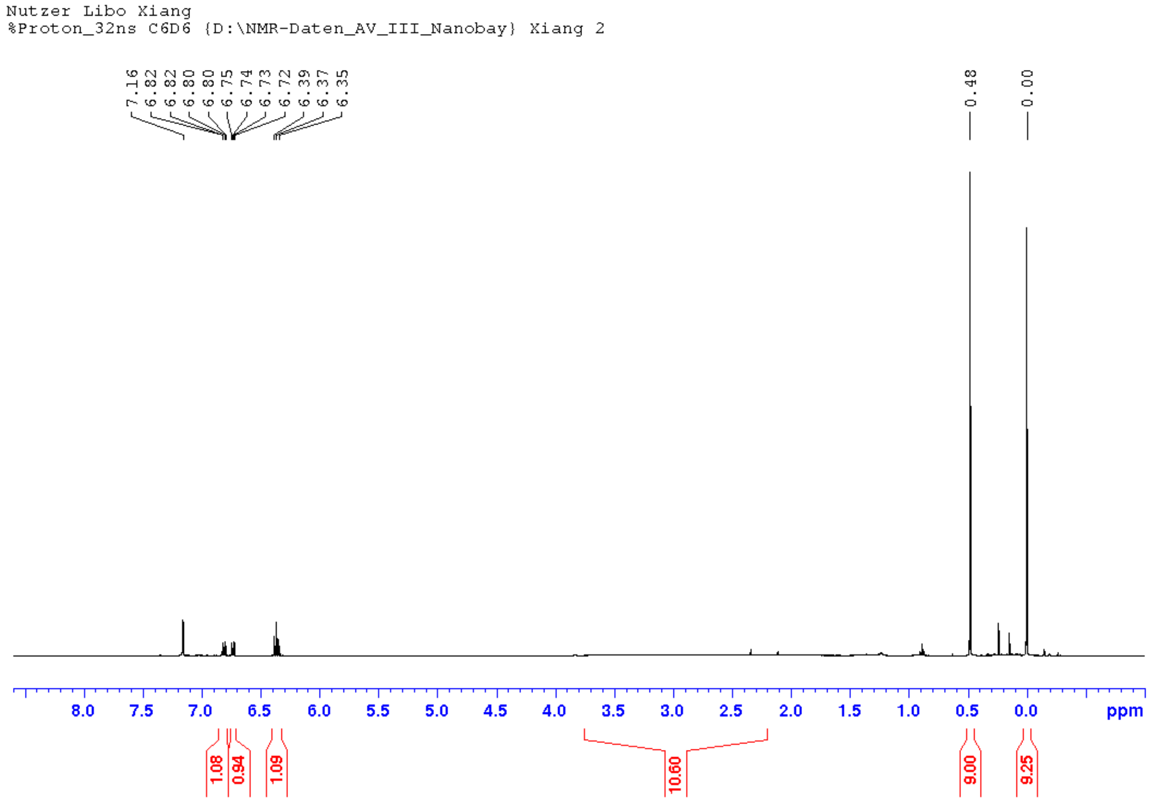

Figure S6. ^{1}H NMR spectrum of **2b** in C_6D_6.

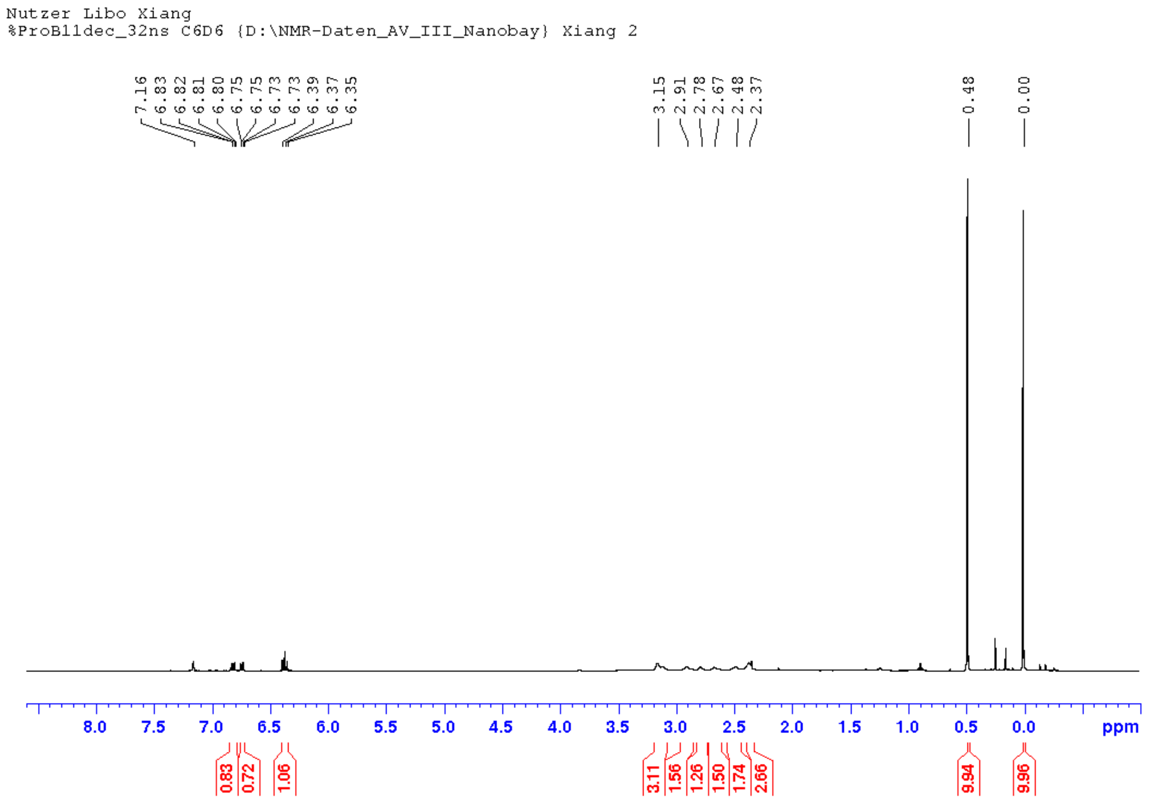

Figure S7. $^{1}H\{^{11}B\}$ NMR spectrum of **2b** in C_6D_6.

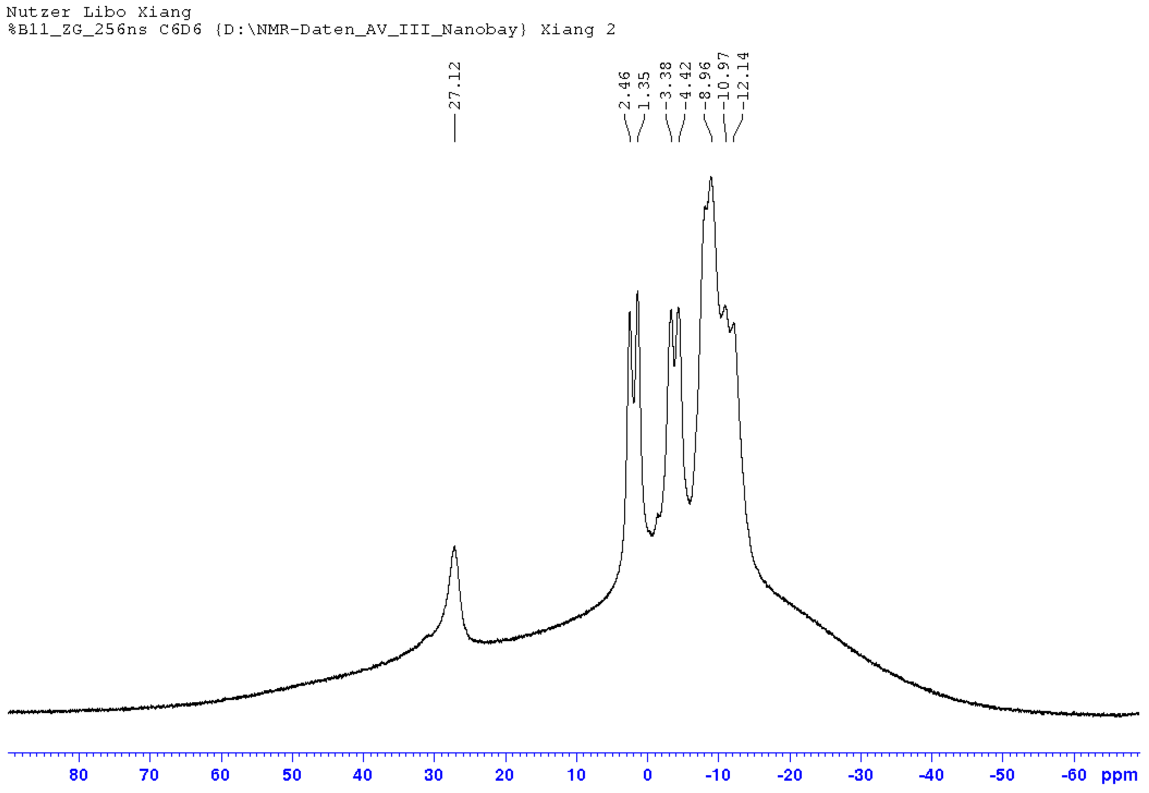

Figure S8. ^{11}B NMR spectrum of **2b** in C_6D_6.

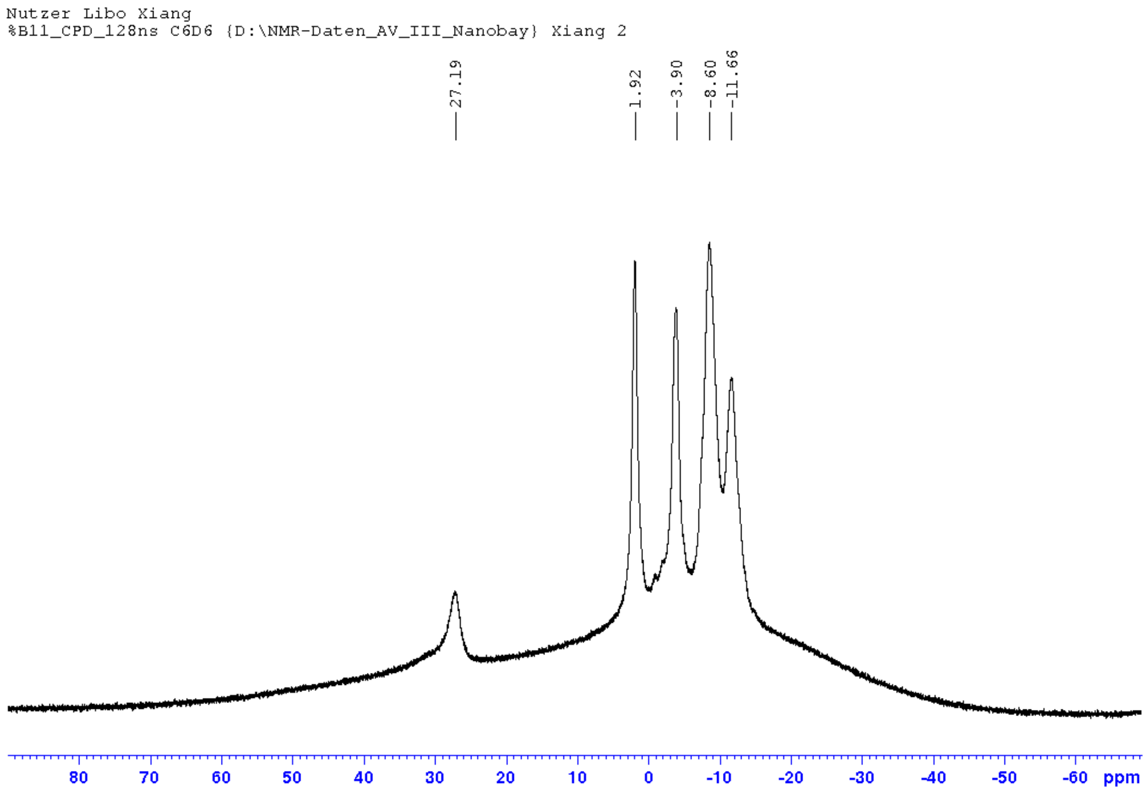

Figure S9. $^{11}B\{^1H\}$ NMR spectrum of **2b** in C_6D_6.

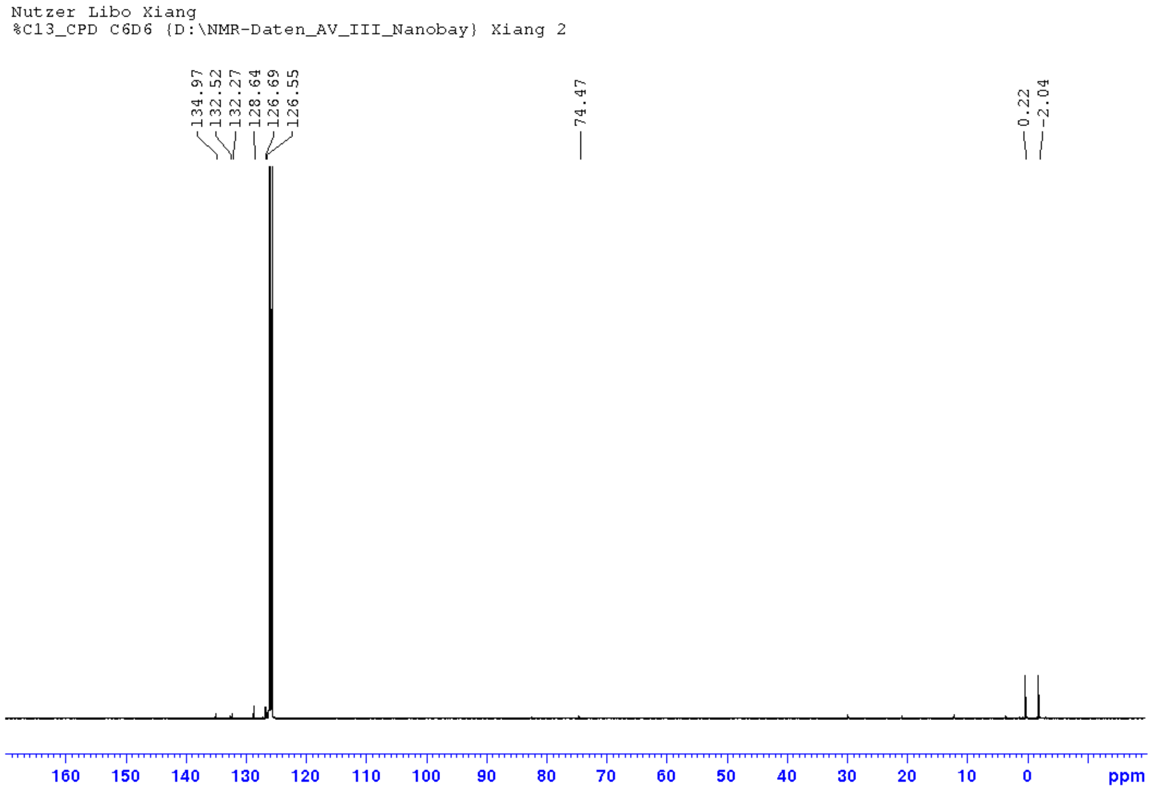

Figure S10. $^{13}C\{^1H\}$ NMR spectrum of **2b** in C_6D_6.

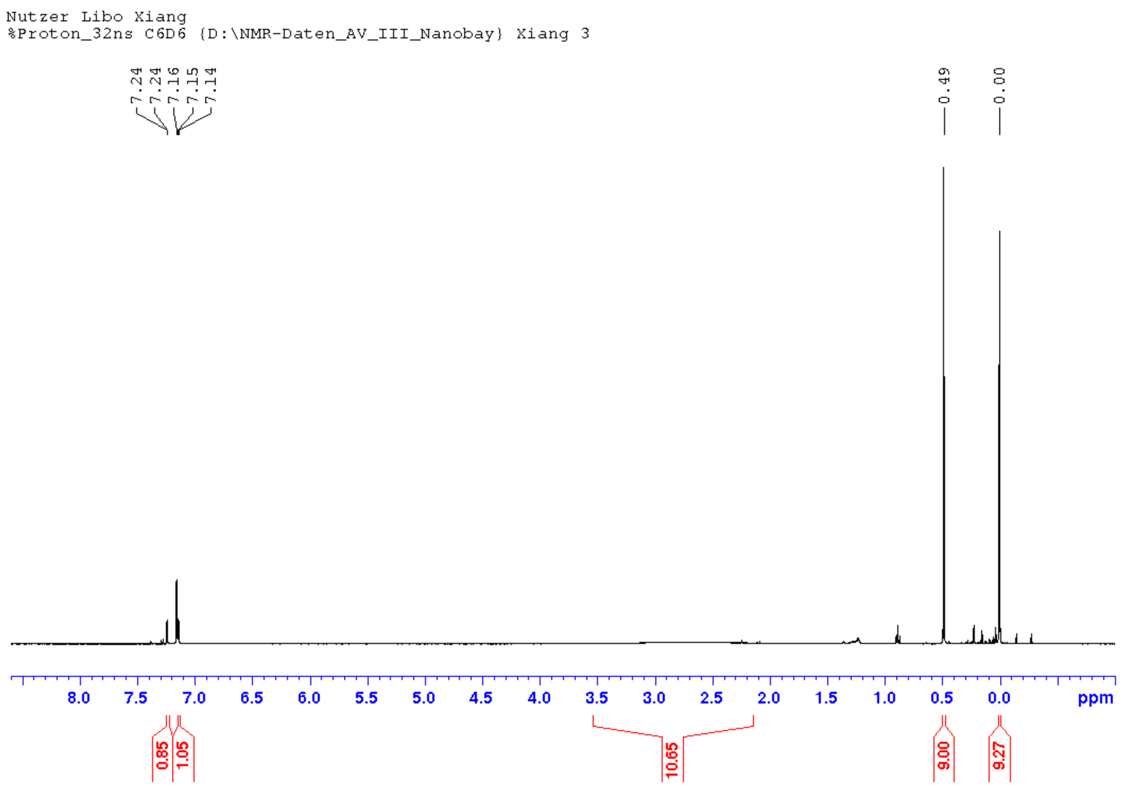

Figure S11. ^{1}H NMR spectrum of **2c** in C_6D_6.

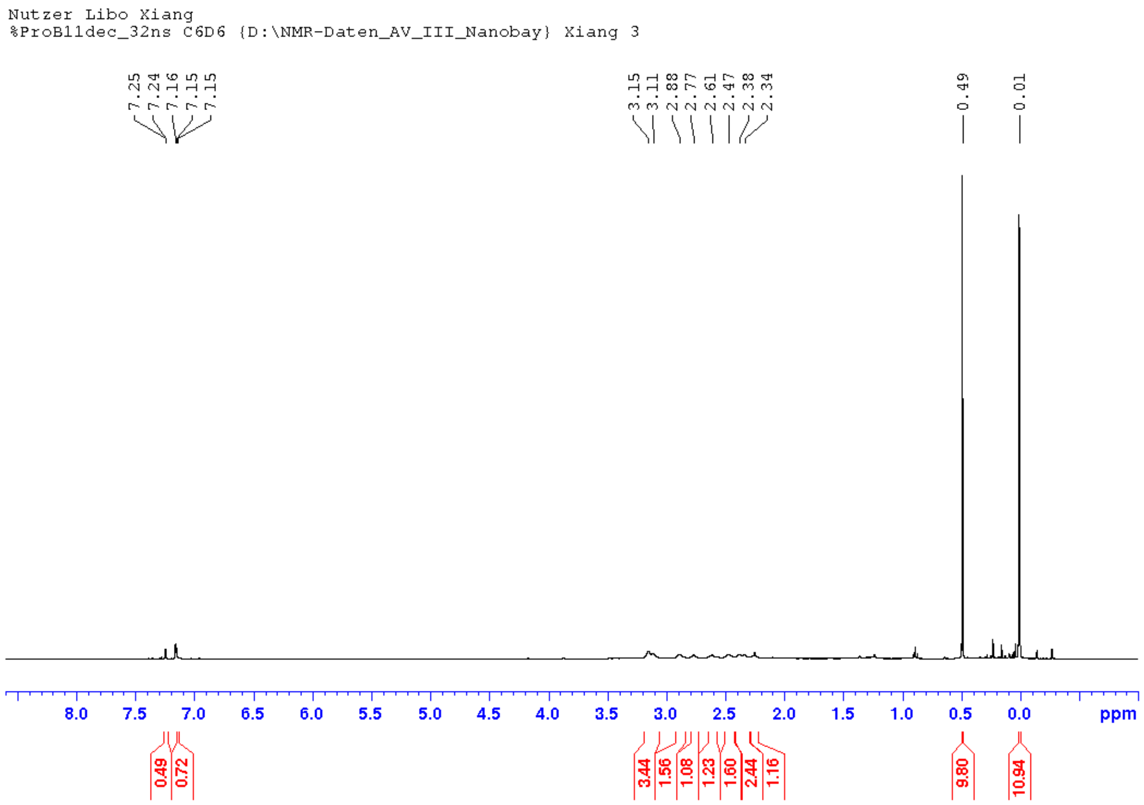

Figure S12. $^{1}H\{^{11}B\}$ NMR spectrum of **2c** in C_6D_6.

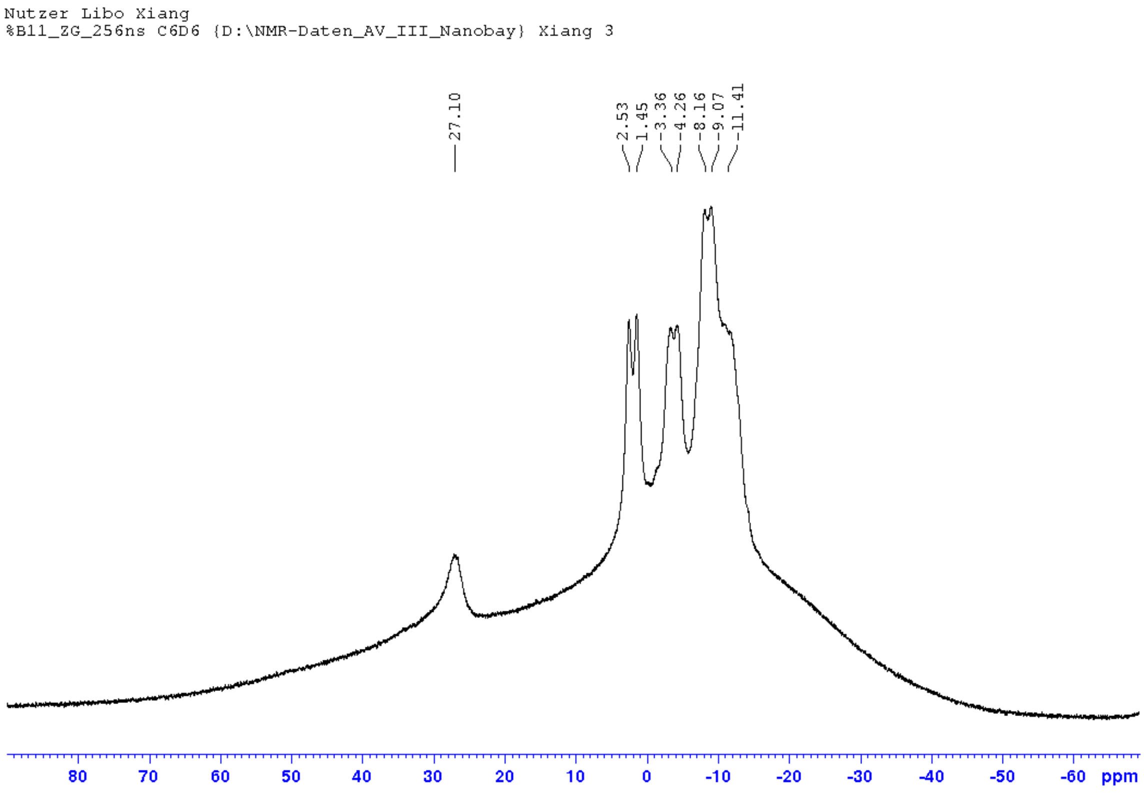

Figure S13. ^{11}B NMR spectrum of **2c** in C_6D_6.

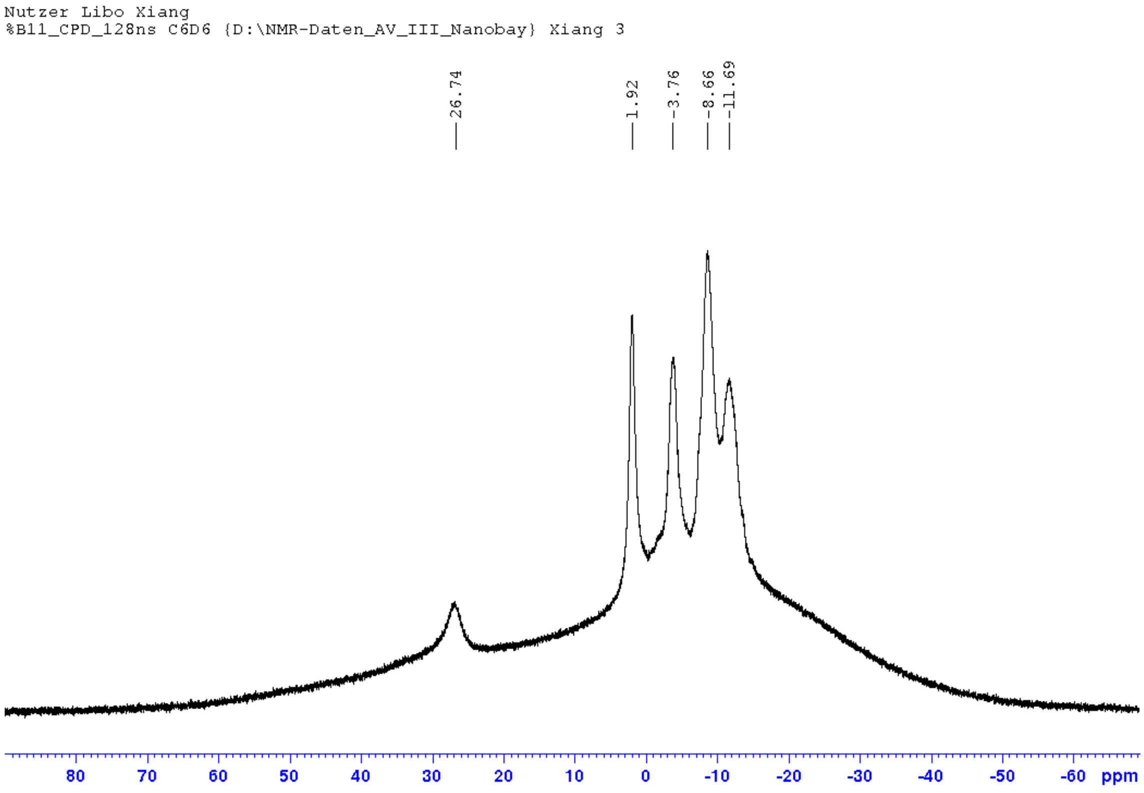

Figure S14. ^{11}B{^{1}H} NMR spectrum of **2c** in C_6D_6.

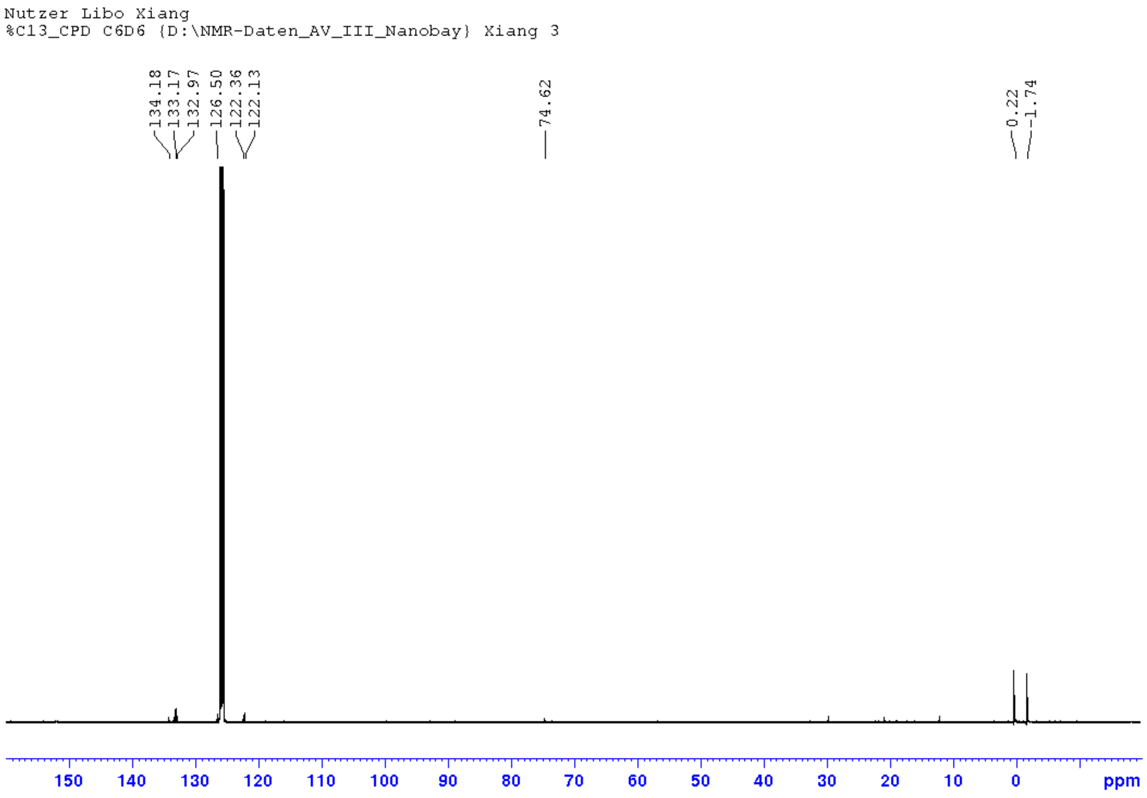

Figure S15. $^{13}C\{^{1}H\}$ NMR spectrum of **2c** in C_6D_6.

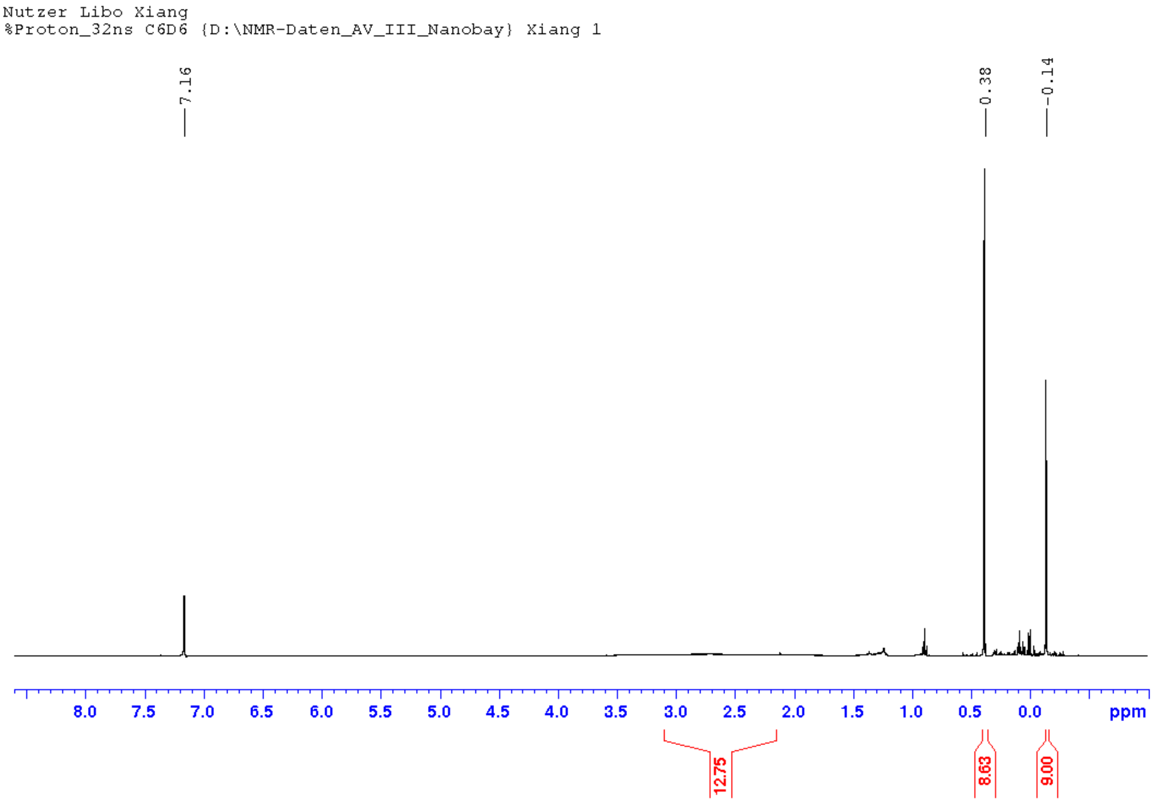

Figure S16. ^{1}H NMR spectrum of **2d** in C_6D_6.

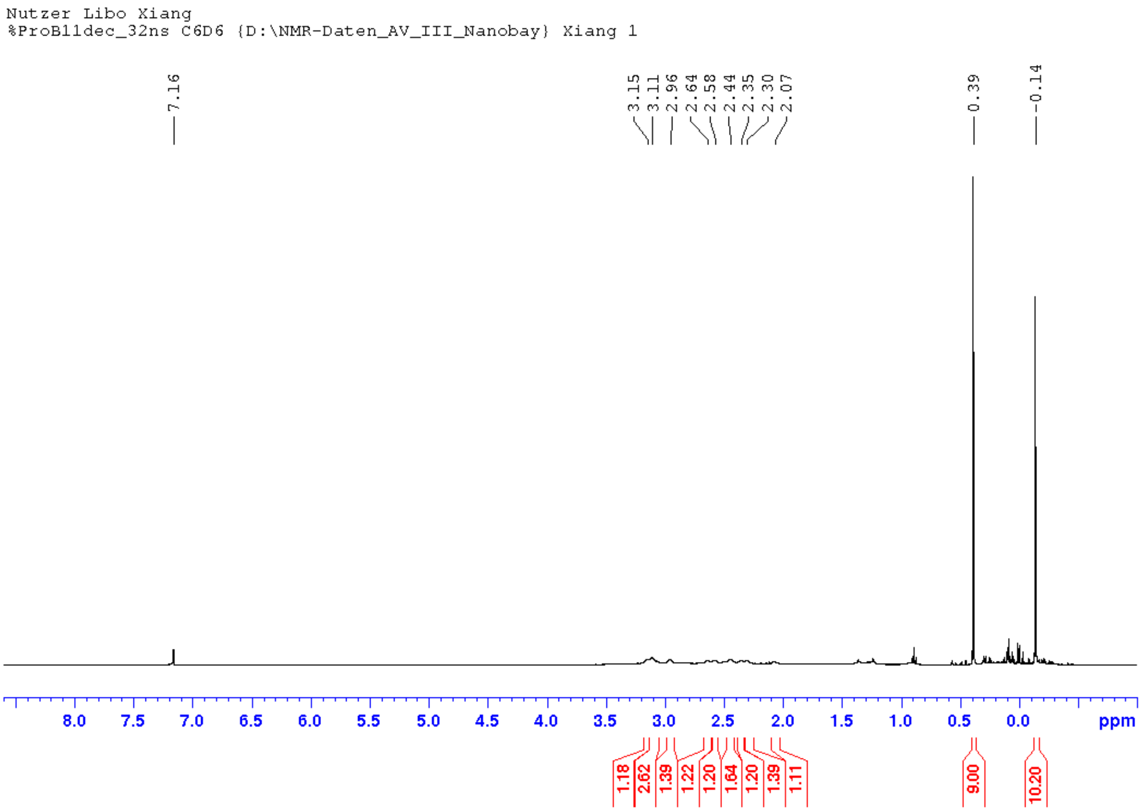

Figure S17. $^{1}H\{^{11}B\}$ NMR spectrum of **2d** in C_6D_6.

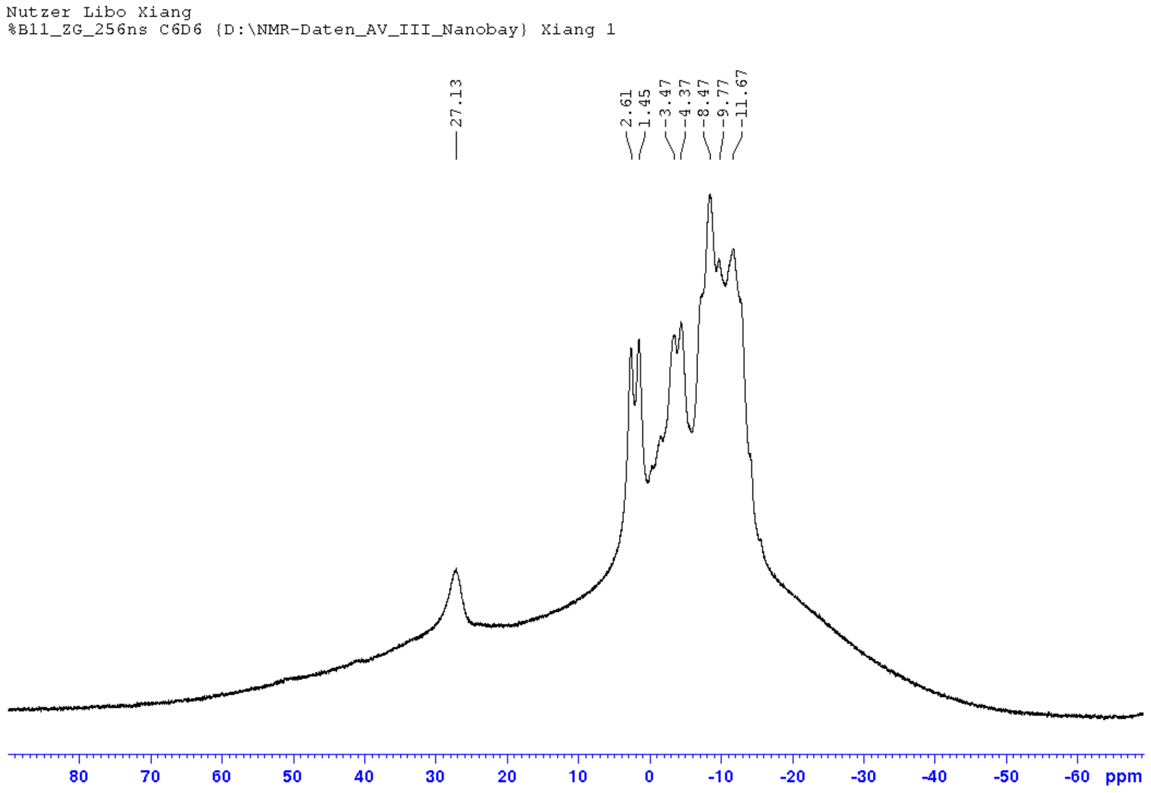

Figure S18. ^{11}B NMR spectrum of **2d** in C_6D_6.

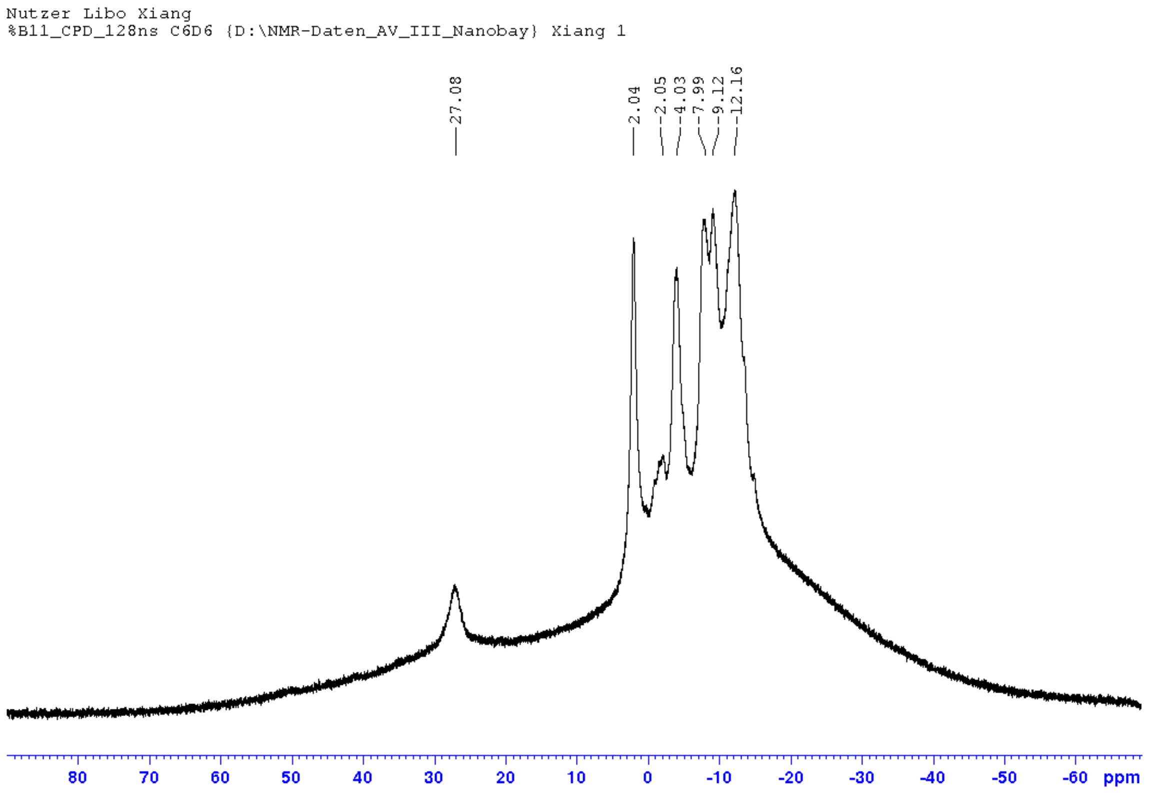

Figure S19. $^{11}B\{^1H\}$ NMR spectrum of **2d** in C_6D_6.

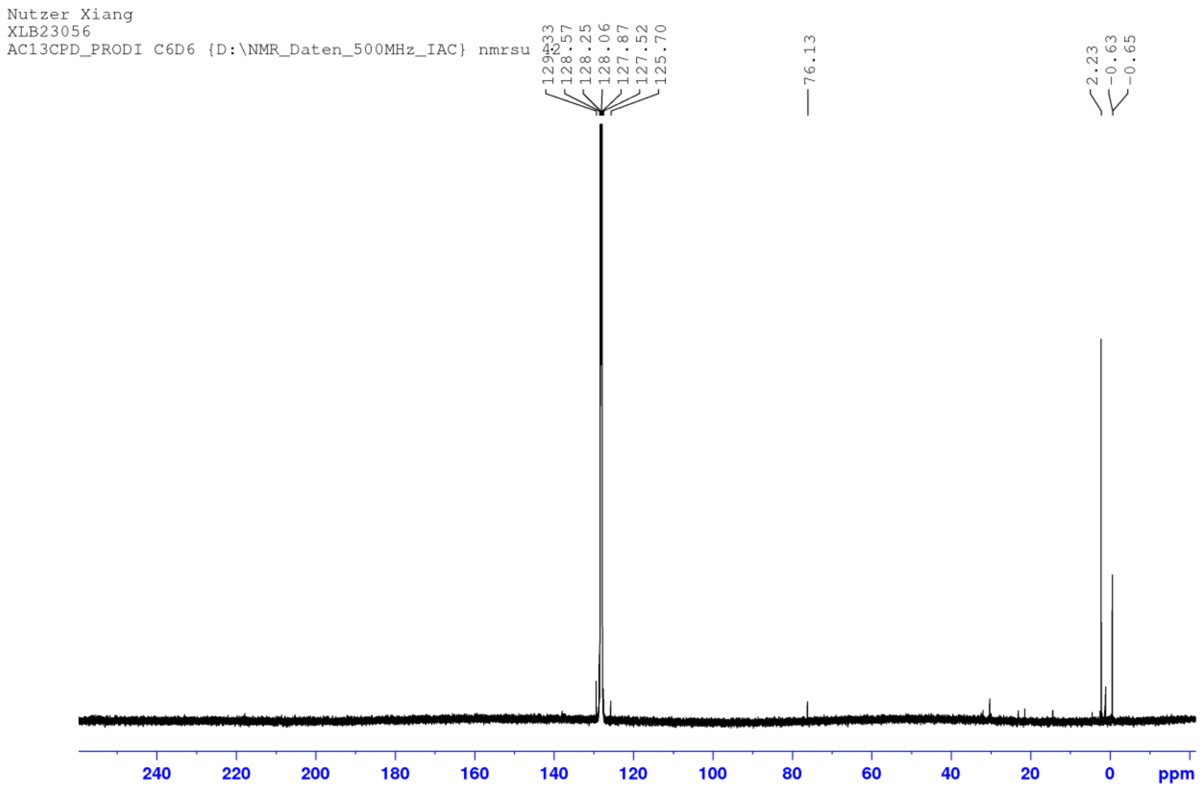

Figure S20. $^{13}C\{^1H\}$ NMR spectrum of **2d** in C_6D_6.

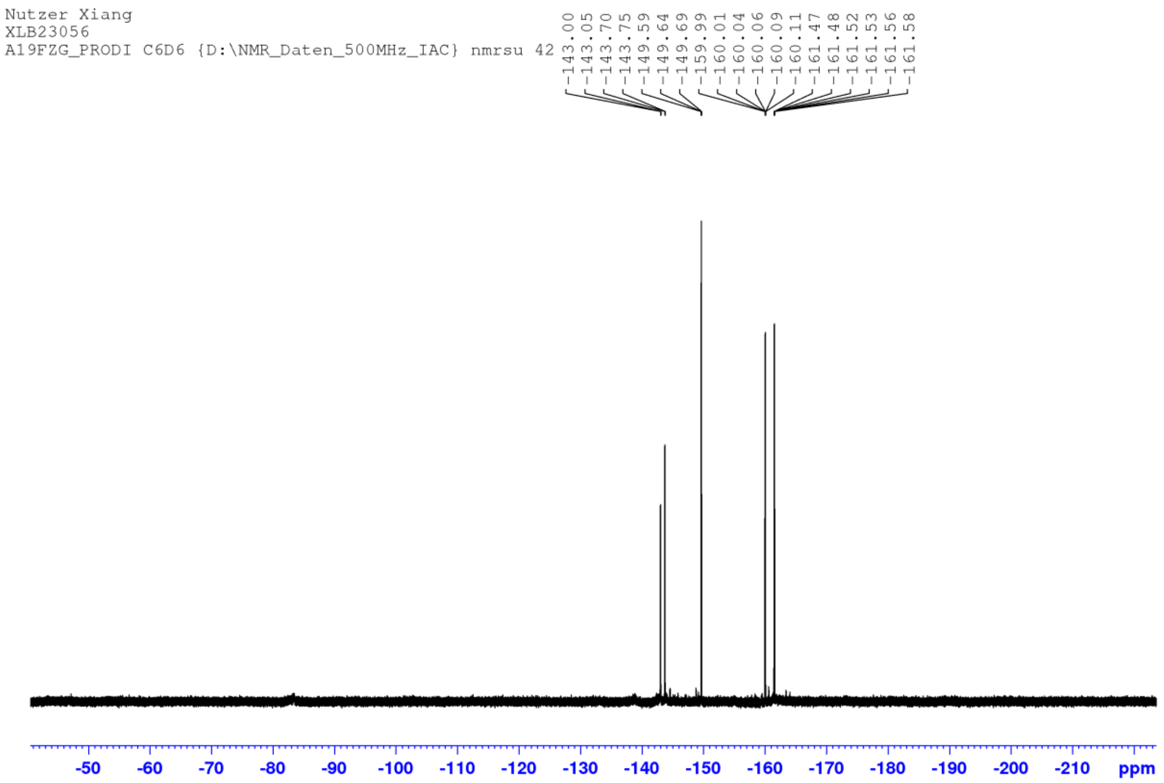

Figure S21. ^{19}F NMR spectrum of **2d** in C_6D_6.

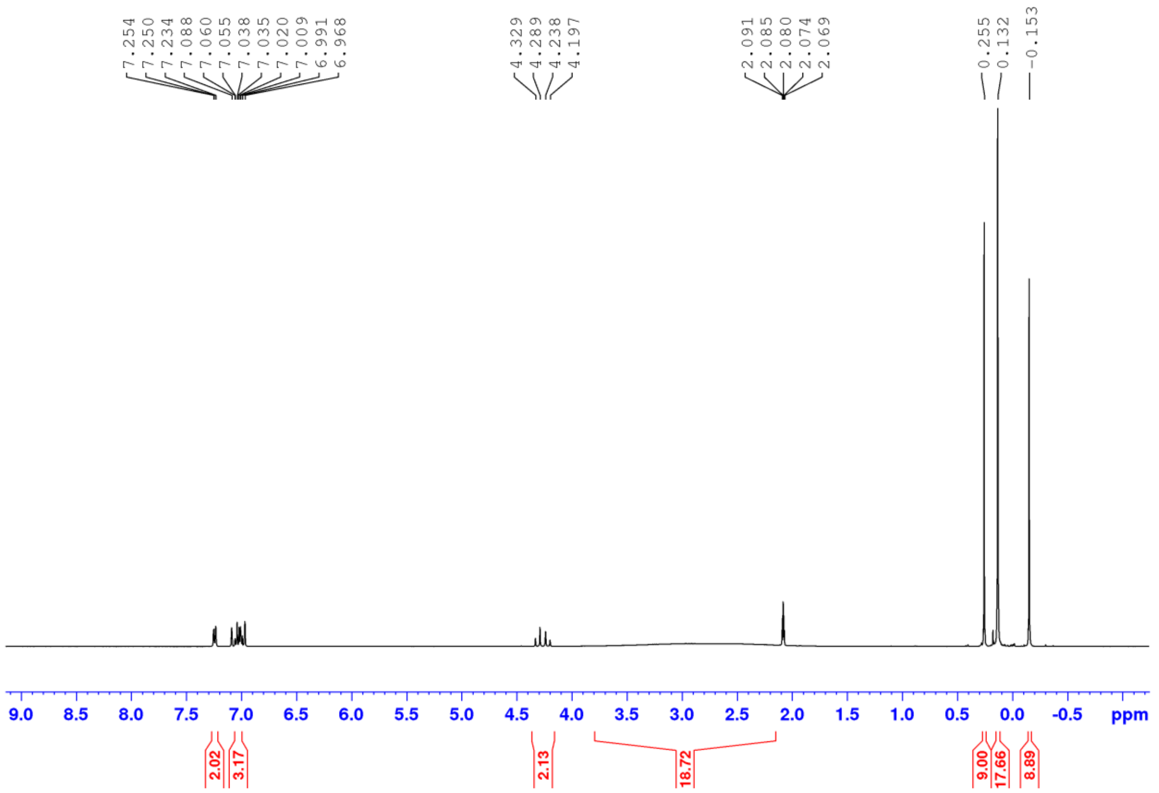

Figure S22. ^{1}H NMR spectrum of **3** in C_6D_6.

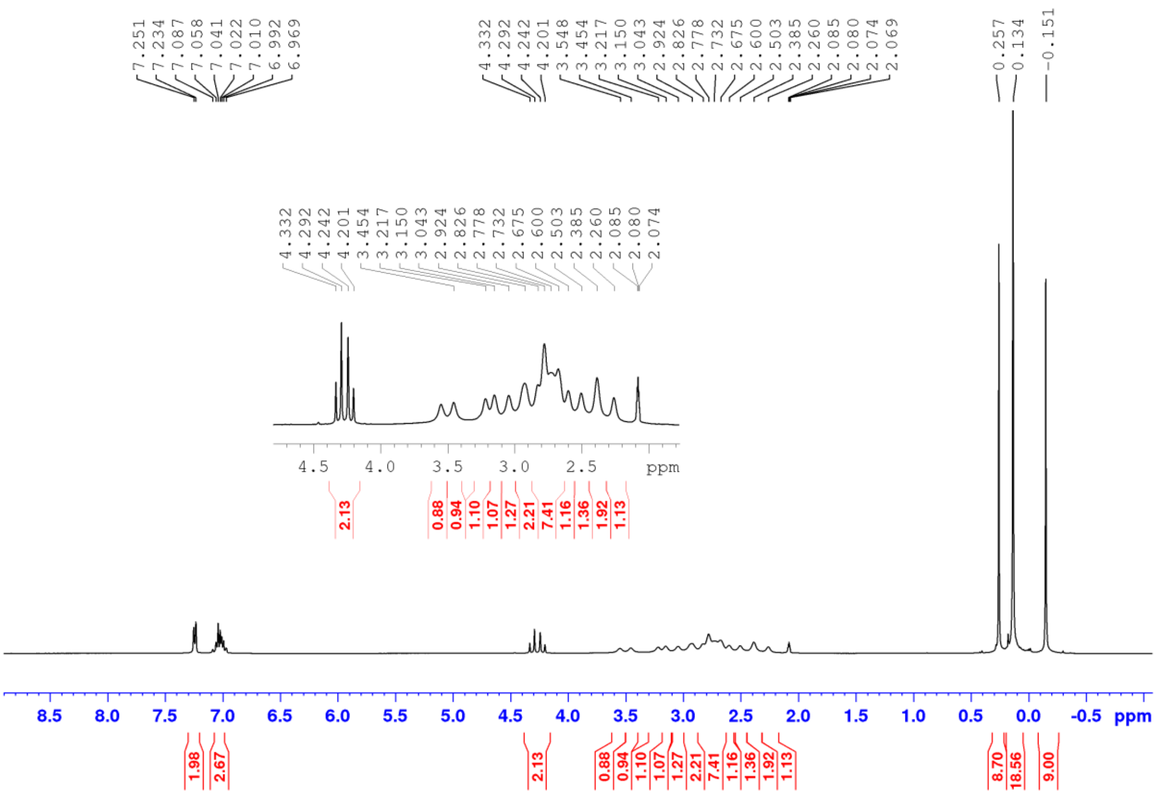

Figure S23. $^{1}H\{^{11}B\}$ NMR spectrum of **3** in C_6D_6.

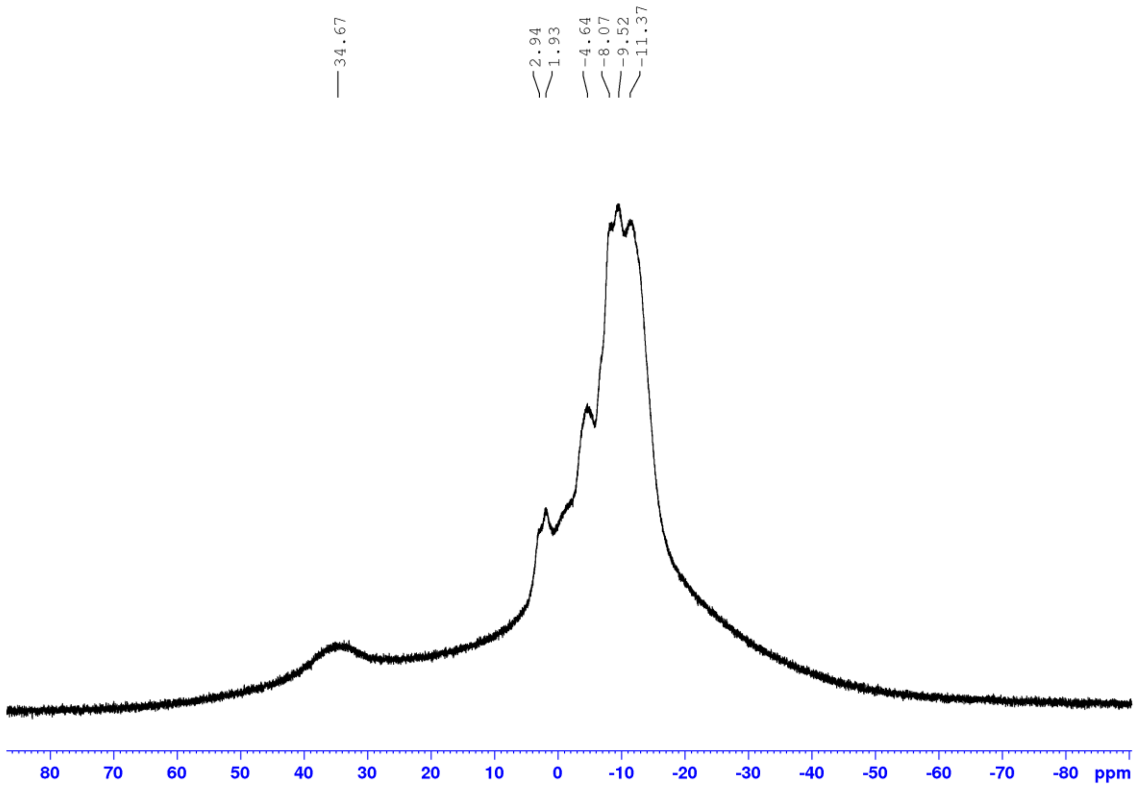

Figure S24. ^{11}B NMR spectrum of **3** in C_6D_6.

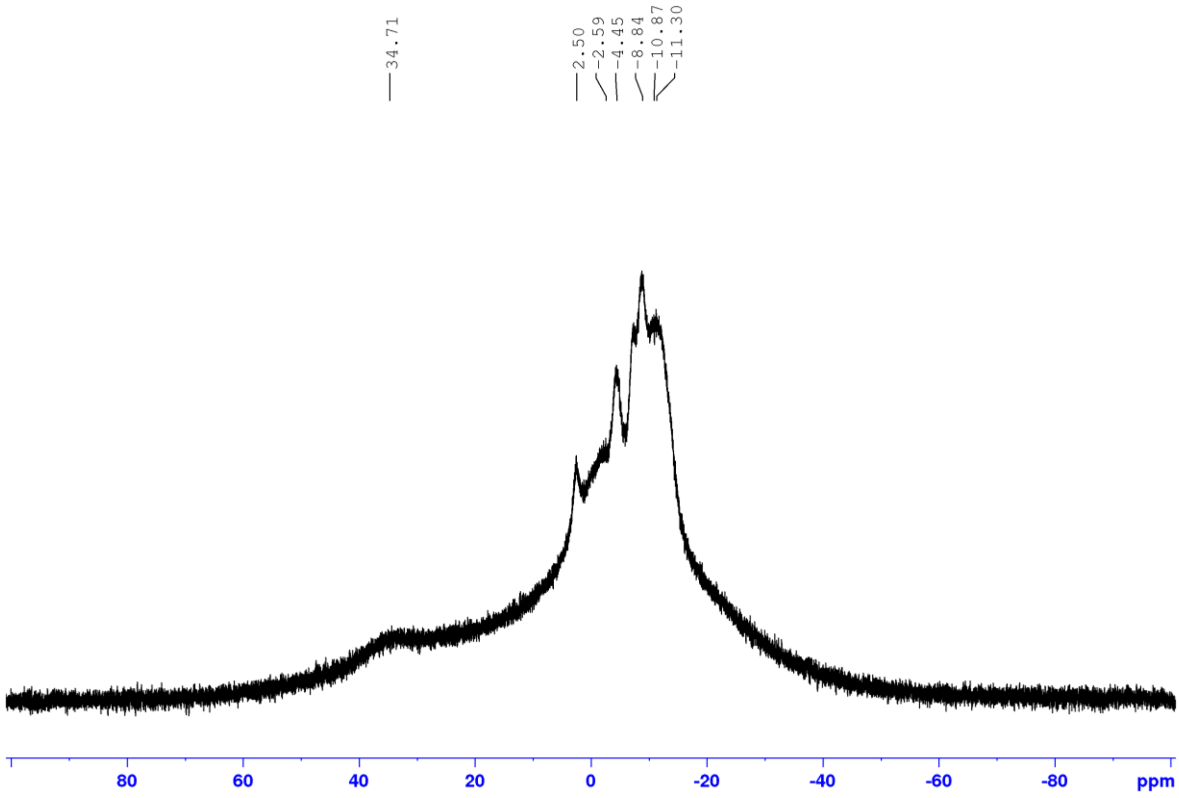

Figure S25. $^{11}B\{^{1}H\}$ NMR spectrum of **3** in C_6D_6.

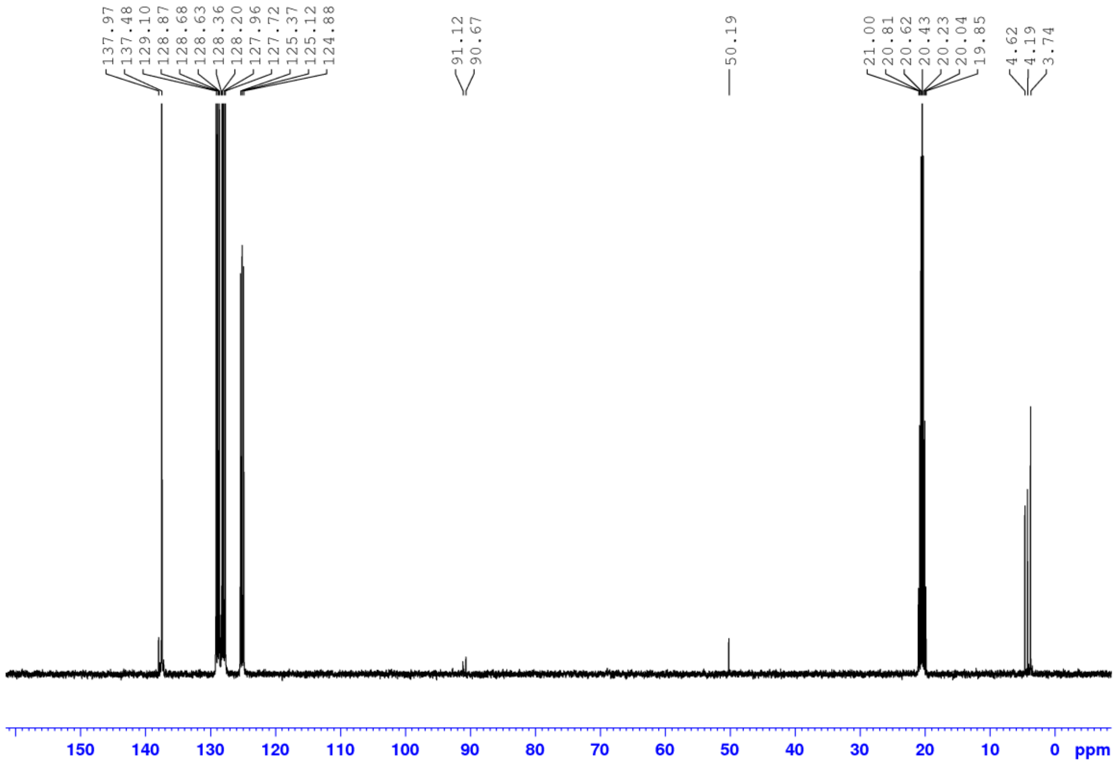

Figure S26. $^{13}C\{^{1}H\}$ NMR spectrum of **3** in C_6D_6.

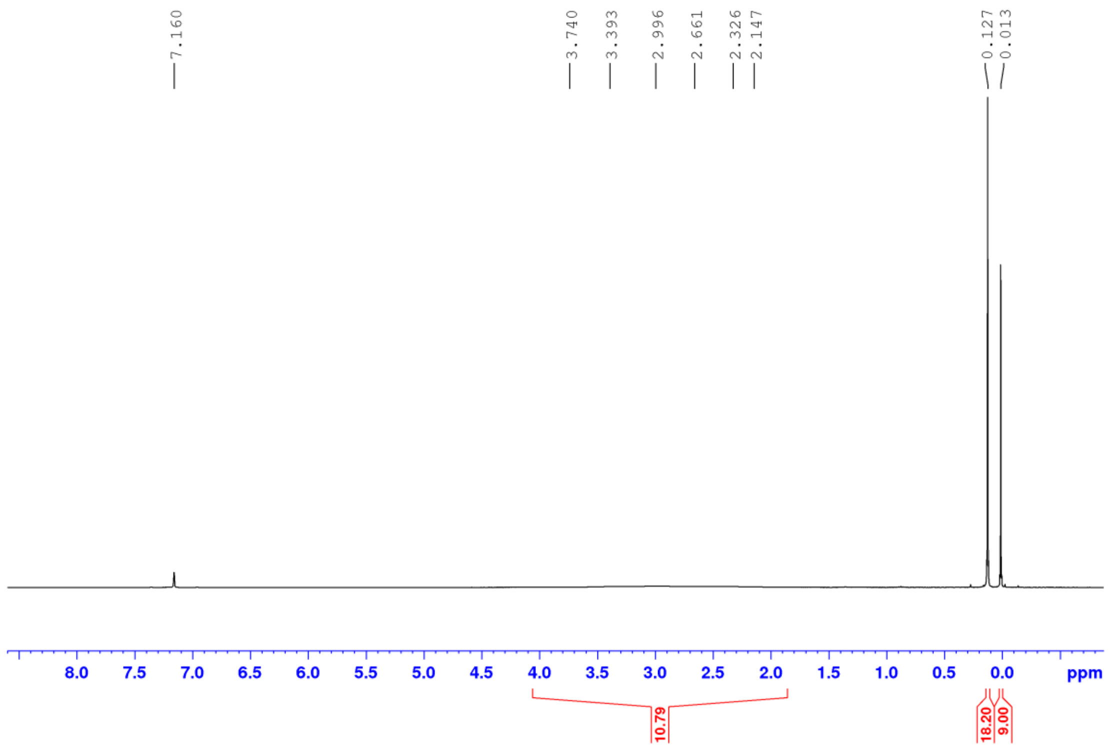

Figure S27. ^{1}H NMR spectrum of **4** in C_6D_6.

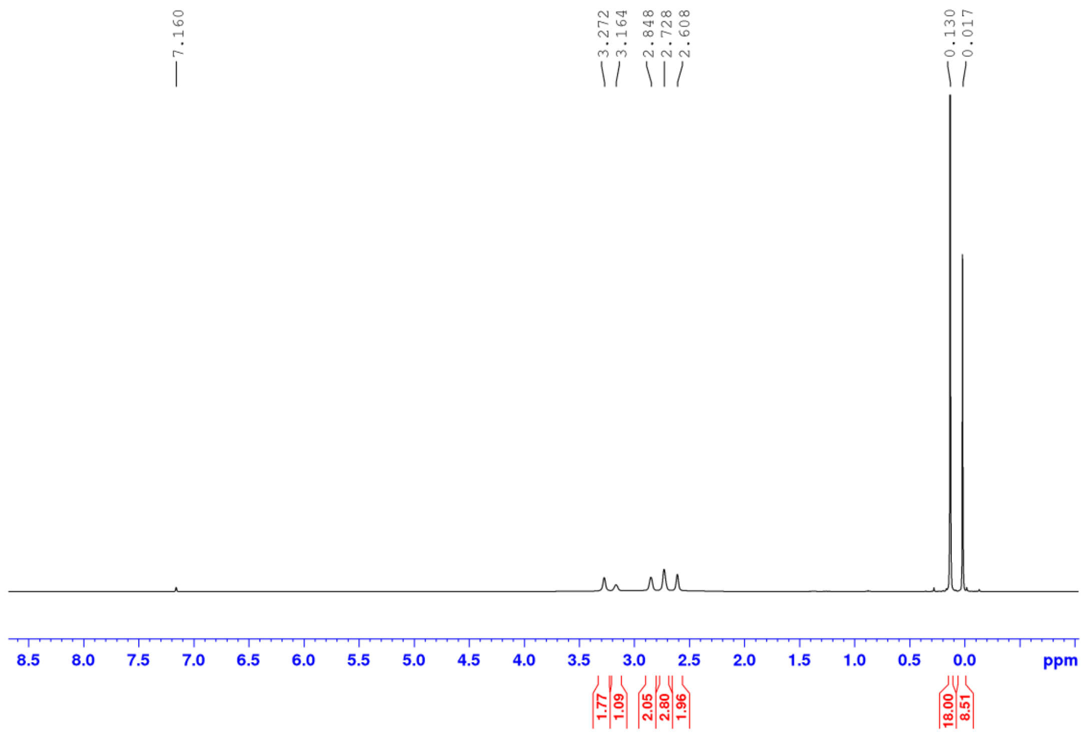

Figure S28. $^{1}H\{^{11}B\}$ NMR spectrum of **4** in C_6D_6.

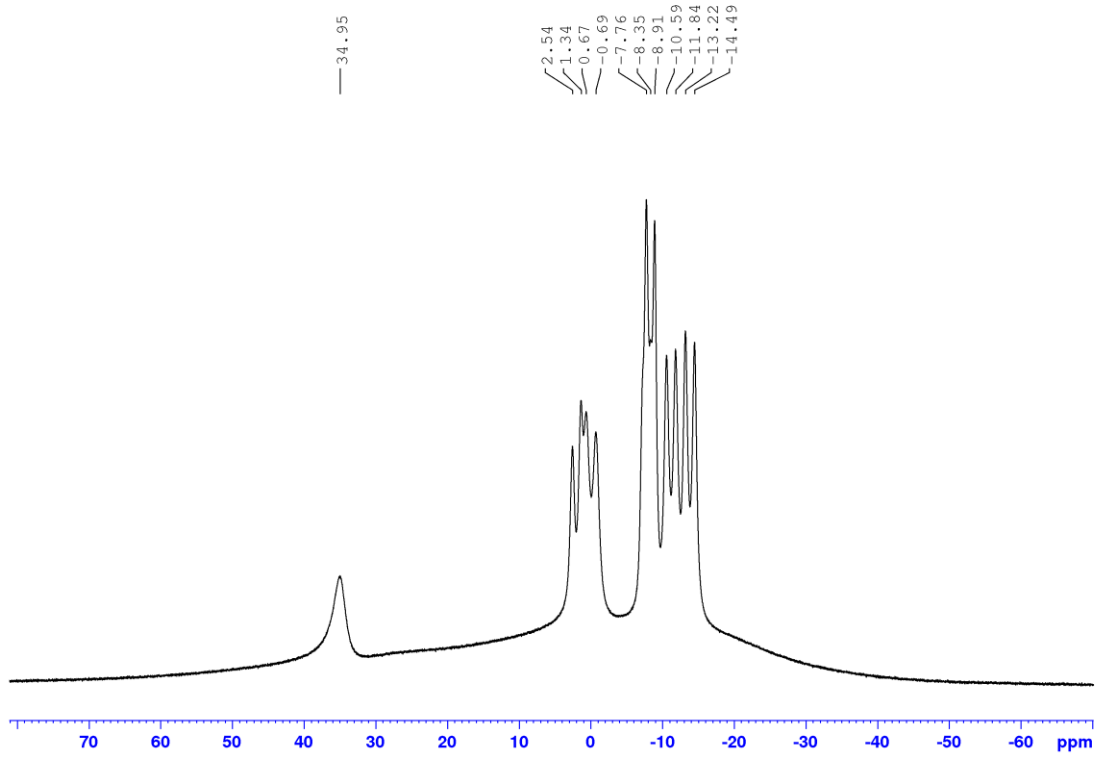

Figure S29. ^{11}B NMR spectrum of **4** in C_6D_6.

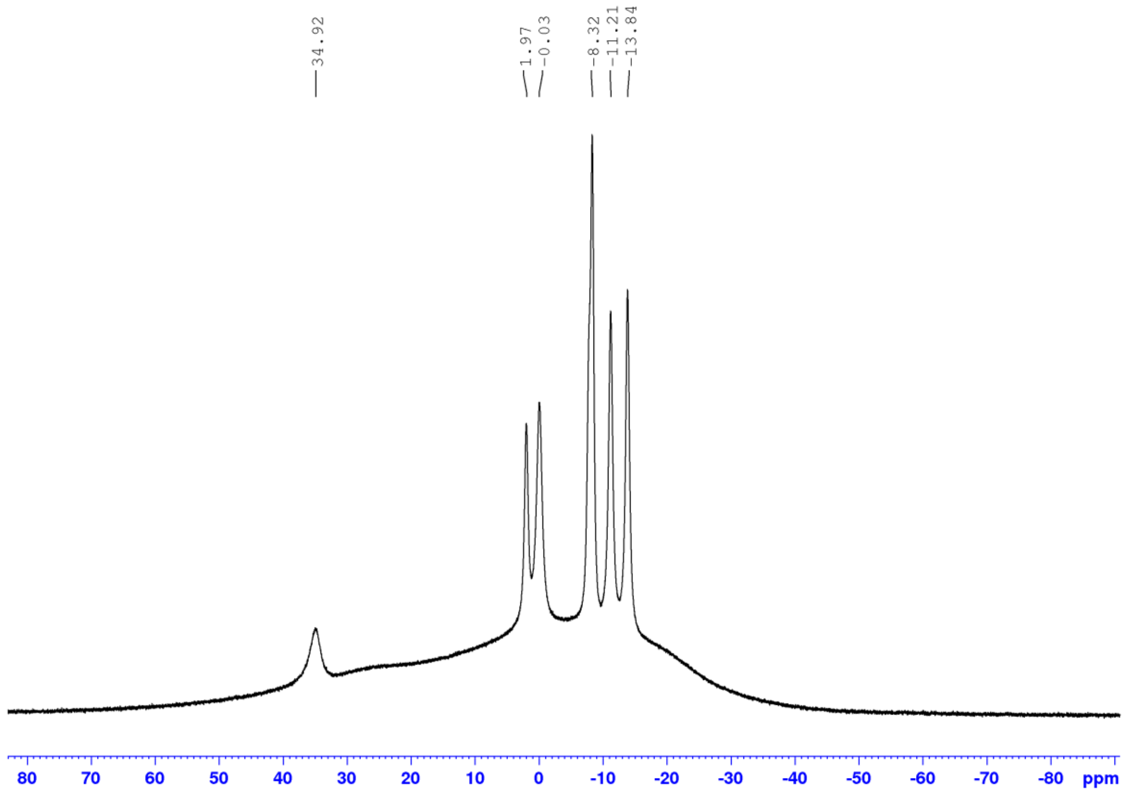

Figure S30. $^{11}B\{^1H\}$ NMR spectrum of **4** in C_6D_6.

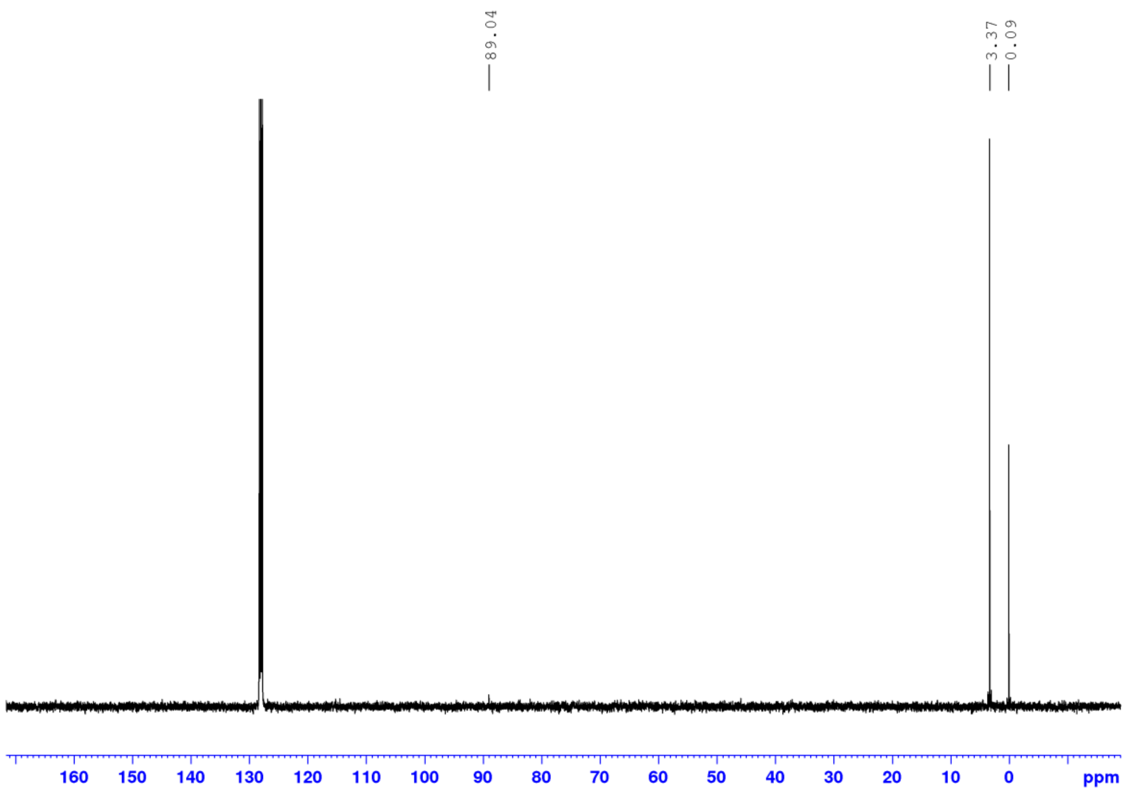

Figure S31 $^{13}C\{^{1}H\}$ NMR spectrum of **4** in C_6D_6.

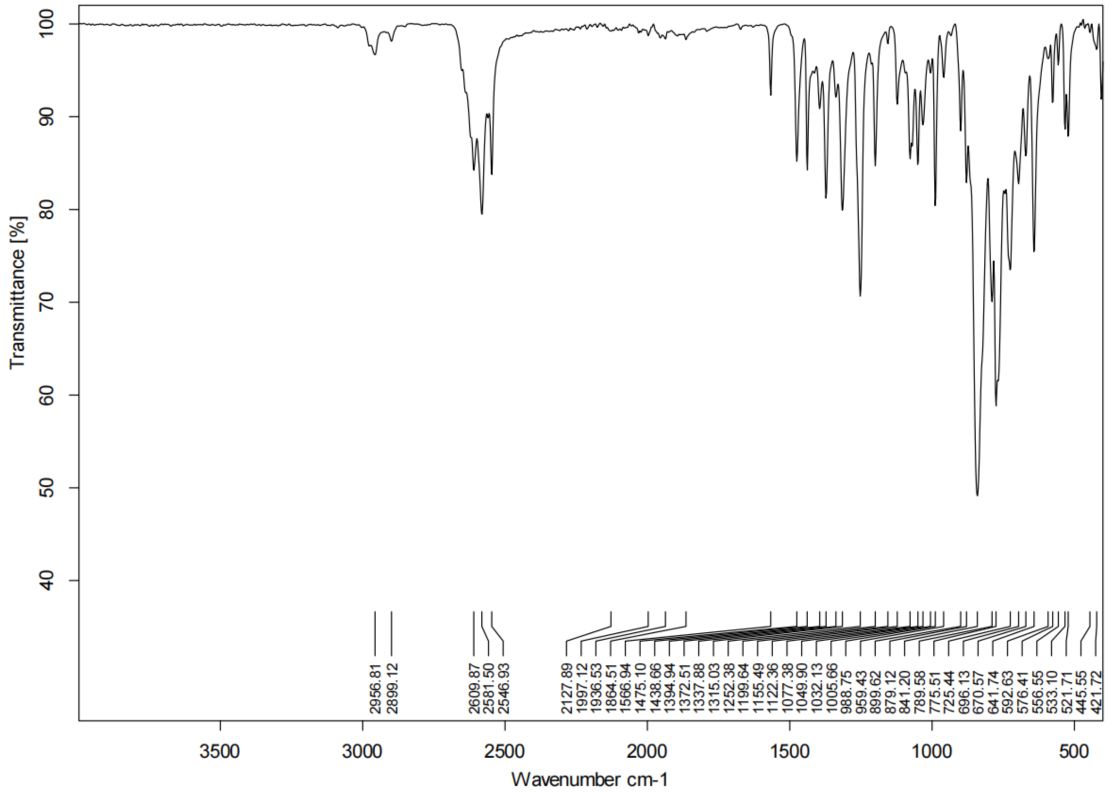

Figure S32. IR spectrum of **2b**.

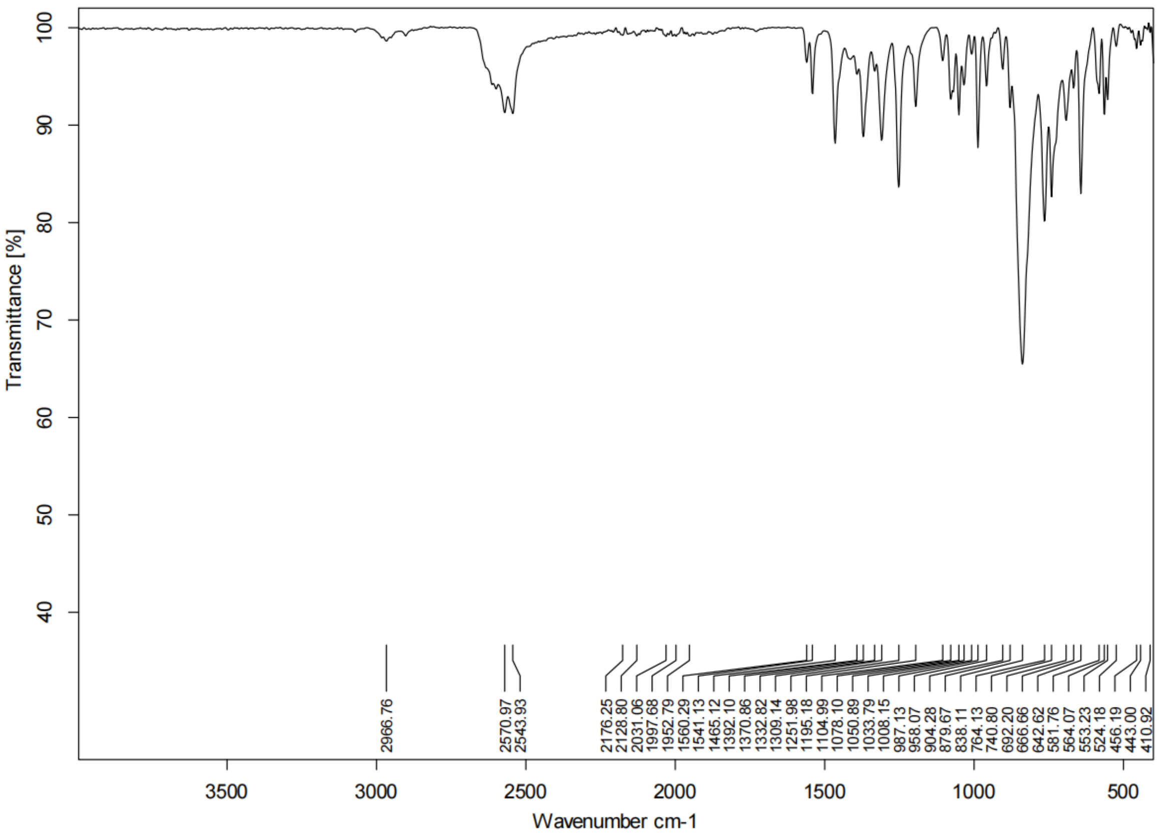

Figure S33. IR spectrum of **2c**.

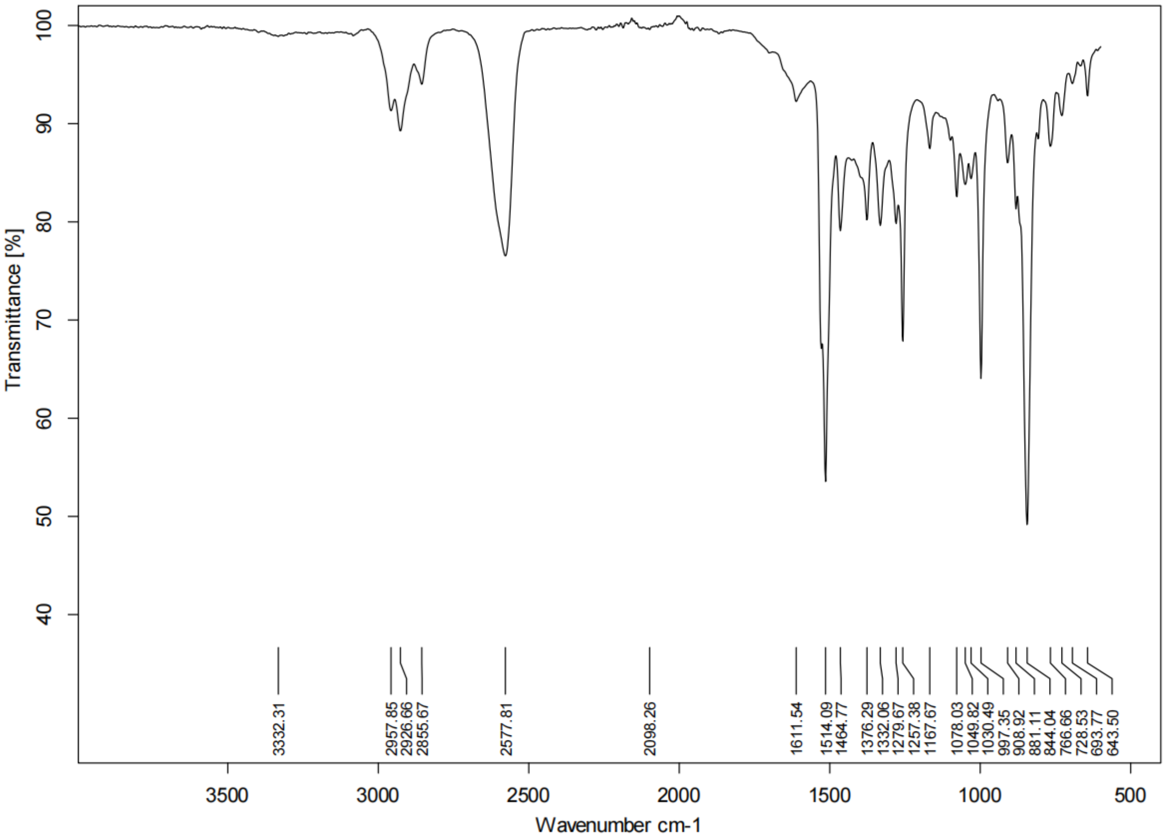

Figure S34. IR spectrum of **2d**.

Crystal structures

The crystal data of **2a**, **3**, **4** were collected on a Bruker D8 VENTURE diffractometer with graphite monochromated Mo Kα radiation (λ = 0.71073 Å) or Cu Kα radiation (λ = 1.54184). Data reduction, scaling and absorption corrections were performed using SAINT (Bruker, V8.38A, 2013). The structure was solved with the XT structure solution program using the Intrinsic Phasing solution method[3] and by using Olex2 as the graphical interface. The model was refined with the ShelXL program[4] using Least Squares minimization. All non-hydrogen atoms were refined anisotropically. Hydrogen atoms were included in structure factor calculations. All hydrogen atoms were assigned to idealized geometric positions.

The crystal data of **2b** were collected on a Rigaku XtaLAB Synergy-R diffractometer with a HPA area detector and multi-layer mirror monochromated CuKa radiation. The structure was solved using intrinsic phasing method5 , refined with the ShelXL program7 and expanded using Fourier techniques.

Crystallographic data have been deposited with the Cambridge Crystallographic Data as supplementary publication nos. CCDC-2278801 (**2a**), 2326703 (**2b**), 2278802 (**3**), 2278803 (**4**). These data can be obtained free of charge from The Cambridge Crystallographic Data Centre via Data https://www.ccdc.cam.ac.uk

Table S1. Crystal data and structure refinement.

Identification code	**2a**	**2b**
Empirical formula	$C_{20}H_{45}B_{11}N_4Si_2$	$C_{14}H_{31}B_{11}N_4Si_2Cl_2$
Formula weight	516.69	501.42
Temperature/K	100	100
Crystal system	triclinic	triclinic
Space group	P-1	P-1
a/Å	12.0996(9)	10.0990(2)
b/Å	15.2353(12)	10.65880(10)
c/Å	17.5608(12)	13.6478(2)
α/°	105.518(3)	85.5810(10)
β/°	90.022(3)	72.4300(10)
γ/°	94.870(3)	72.3450(10)
Volume/Å^3	3107.0(4)	1334.42(4)
Z	4	2
ρ_{calc}g/cm^3	1.105	1.248
μ/mm^{-1}	0.132	3.125
F(000)	1104.0	520.0
Crystal size/mm^3	0.12 × 0.11 × 0.07	0.13 × 0.1 × 0.09
Radiation	MoKα (λ = 0.71073)	CuKα (λ = 1.54184)
2Θ range for data collection/°	4.484 to 50	6.794 to 150.44
Index ranges	-14 ≤ h ≤ 14, -17 ≤ k ≤ 18, -20 ≤ l ≤ 20	-12 ≤ h ≤ 12, -12 ≤ k ≤ 10, -16 ≤ l ≤ 16
Reflections collected	10761	27112
Independent reflections	10761 [R_{int} = 0.1022, R_{sigma} = 0.0905]	5344 [R_{int} = 0.0322, R_{sigma} = 0.0189]
Data/restraints/parameters	10761/0/687	5344/0/304
Goodness-of-fit on F^2	1.093	1.070
Final R indexes [I>=2σ (I)]	R_1 = 0.0947, wR_2 = 0.2072	R_1 = 0.0295, wR_2 = 0.0817
Final R indexes [all data]	R_1 = 0.1314, wR_2 = 0.2209	R_1 = 0.0310, wR_2 = 0.0832
Largest diff. peak/hole / e Å^{-3}	0.86/-0.48	0.35/-0.28

Table S2. Crystal data and structure refinement.

Identification code	**3**	**4**
Empirical formula	$C_{23}H_{63}B_{22}N_5Si_4$	$C_{11}H_{37}B_{11}N_2Si_3$
Formula weight	759.96	400.60
Temperature/K	100	100
Crystal system	monoclinic	monoclinic
Space group	$P2_1/n$	$P2_1/c$
a/Å	18.6315(18)	9.9271(3)
b/Å	11.0376(10)	24.7750(6)
c/Å	22.7392(17)	10.7243(3)
α/°	90	90
β/°	108.033(3)	109.3940(10)
γ/°	90	90
Volume/Å^3	4446.5(7)	2487.91(12)
Z	4	4
ρ_{calc}g/cm^3	1.135	1.070
μ/mm^{-1}	0.161	0.192
F(000)	1608.0	856.0
Crystal size/mm^3	0.2 × 0.12 × 0.12	0.4 × 0.4 × 0.4
Radiation	MoKα (λ = 0.71073)	MoKα (λ = 0.71073)
2Θ range for data collection/°	4.348 to 50	4.65 to 49.518
Index ranges	-22 ≤ h ≤ 20, -13 ≤ k ≤ 13, -26 ≤ l ≤ 27	-11 ≤ h ≤ 11, -29 ≤ k ≤ 29, -12 ≤ l ≤ 12
Reflections collected	49782	41430
Independent reflections	7800 [R_{int} = 0.1041, R_{sigma} = 0.0621]	4255 [R_{int} = 0.0540, R_{sigma} = 0.0243]
Data/restraints/parameters	7800/0/499	4255/0/253
Goodness-of-fit on F^2	1.076	1.049
Final R indexes [I>=2σ (I)]	R_1 = 0.0457, wR_2 = 0.1158	R_1 = 0.0316, wR_2 = 0.0822
Final R indexes [all data]	R_1 = 0.0795, wR_2 = 0.1297	R_1 = 0.0357, wR_2 = 0.0854
Largest diff. peak/hole / e Å^{-3}	0.33/-0.33	0.38/-0.33

Computational details

All calculations were performed with the Gaussian 09 program.[5] GaussView was used to visualize the results, to measure calculated structural parameters, and to plot orbital surfaces. All structures were optimized using the B3LYP hybrid functional[6] and 6-31G* basis set,[7] with the D3 dispersion correction.[8] Frequency calculations were performed to confirm that a transition state has only one imaginary frequency, while a local minimum has no imaginary frequency. Intrinsic reaction coordinate (IRC) calculations[9] were also carried out to further confirm that transition states can link the relevant local minima. Solvent effects of toluene were considered by carrying out single-point energy calculations using the SMD model[10] at the B3LYP-D3/6-311G** level.[11]

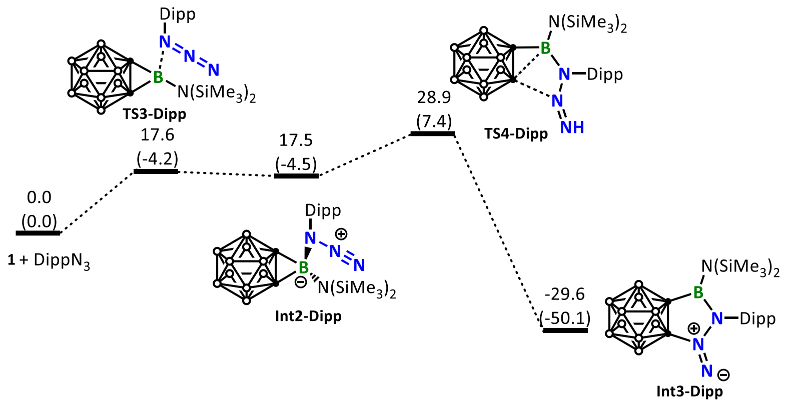

Figure S35. Energy profile calculated for the reaction of **1** with DippN$_3$ leading to the formation of **Int3-Dipp**. Relative free energies and electronic energies (in parentheses) are given in kcal/mol.

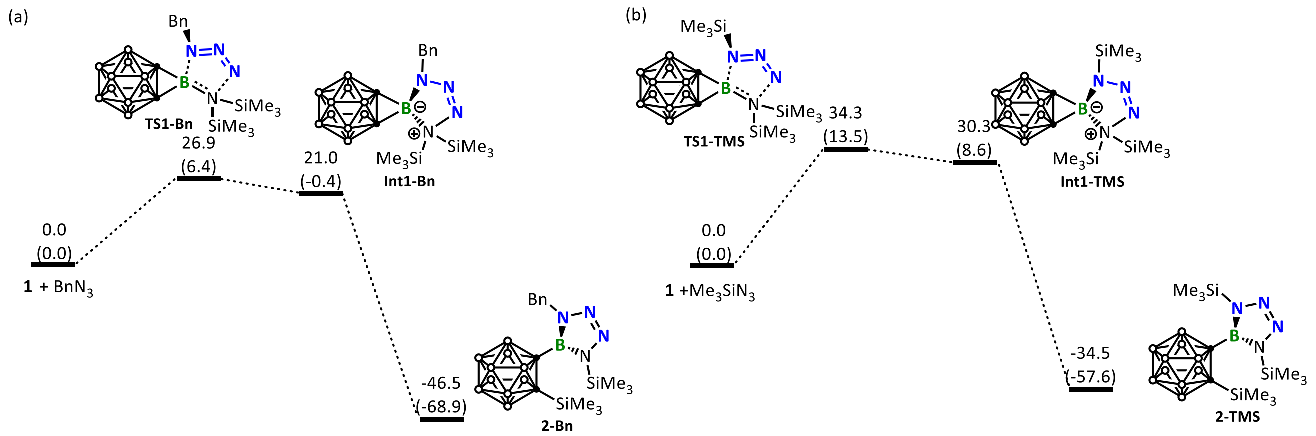

Figure S36. Energy profile calculated for the reaction of **1** with (a) BnN$_3$ and (b) SiMe$_3$N$_3$ leading to the [3+2] cycloaddition intermediate **2-Bn** and **2-TMS**, respectively. Relative free energies and electronic energies (in parentheses) are given in kcal/mol.

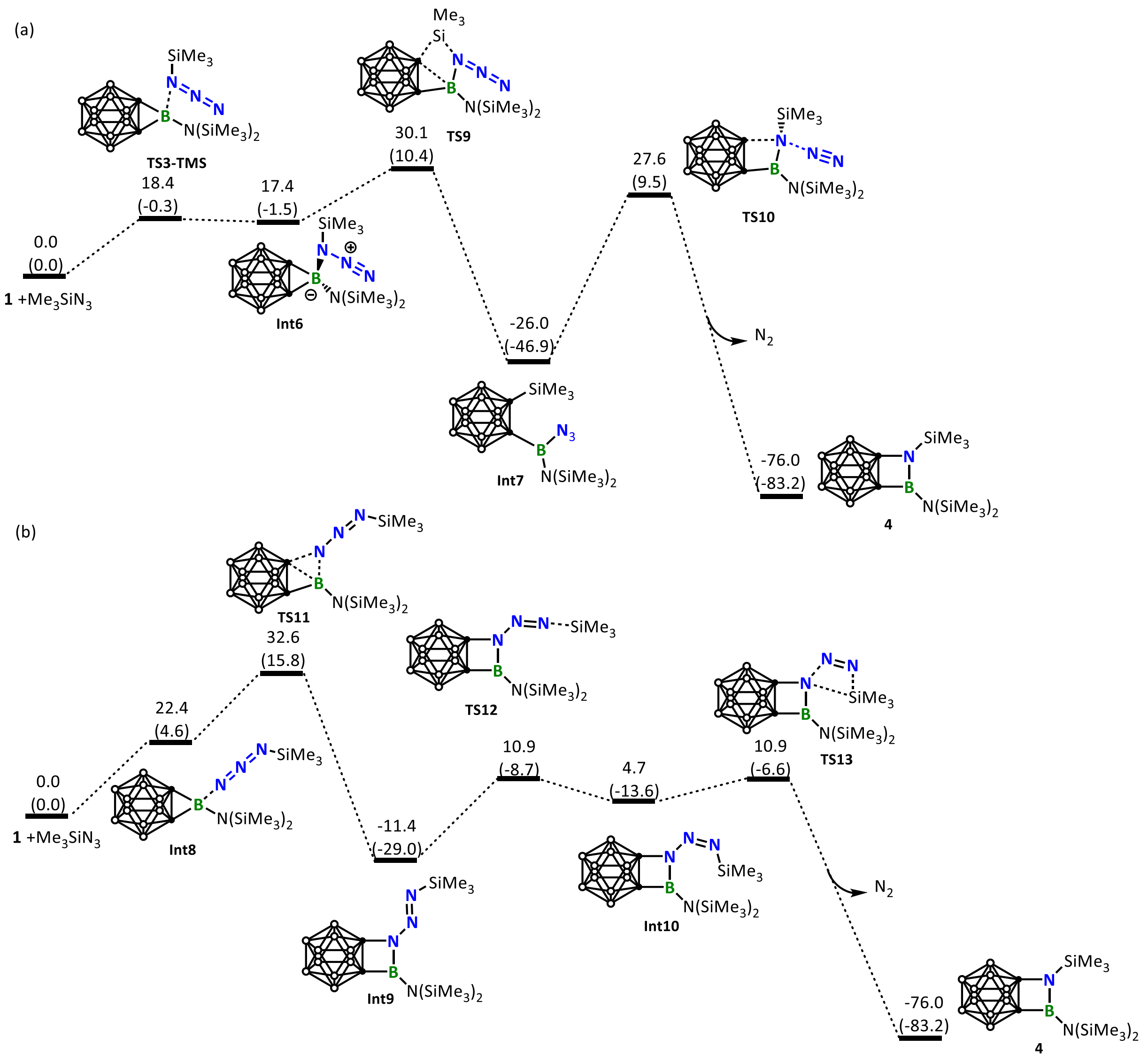

Figure S37. Energy profiles of less favorable pathways calculated for the reaction of **1** with Me_3SiN_3 leading to the formation of **4**. Relative free energies and electronic energies (in parentheses) are given in kcal/mol.

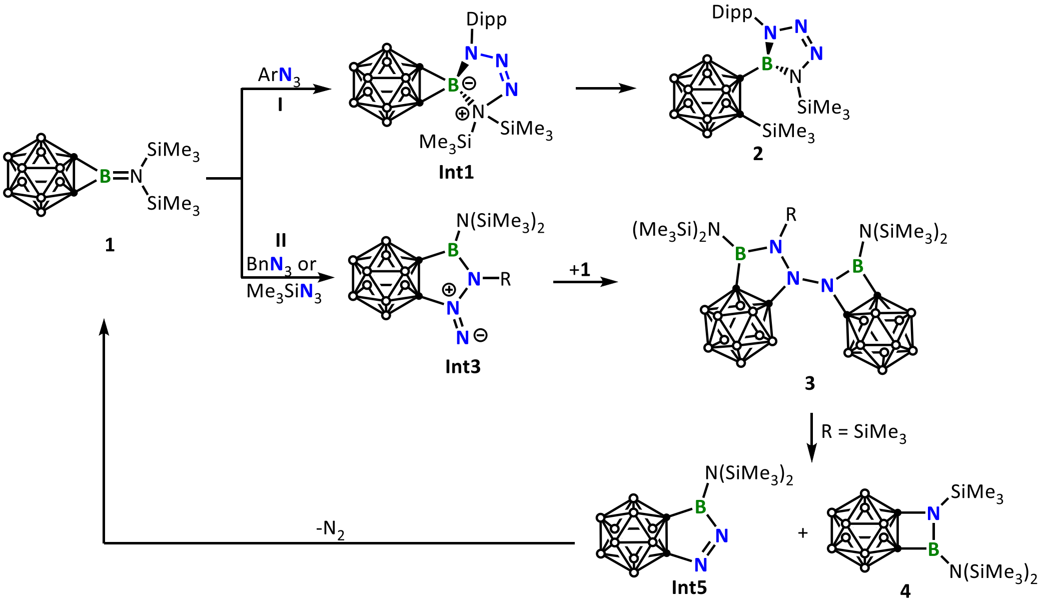

Figure S38. Summary of reaction mechanisms between **1** and RN_3.

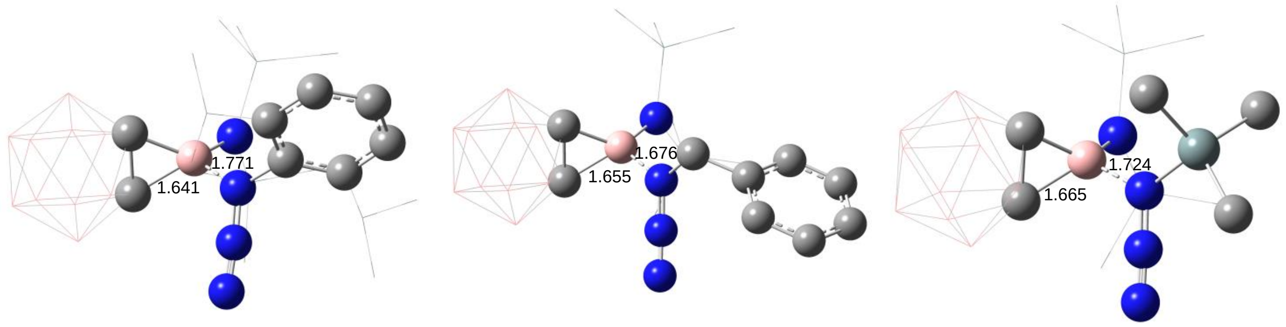

Figure S39. Bond distances of B–C and B–N$_\alpha$ (Å) in optimized RN$_3$-**1** adducts.

Cartesian coordinates

1

```
 SCF Done: -1229.348264
 Si                 1.98703700   -1.65257300   -0.00180700
 Si                 2.23741600    1.57096400   -0.00815000
 N                  1.31550600    0.02648000   -0.07684300
 C                 -1.39065600   -0.76401500   -0.00953000
 C                 -1.39491000    0.87194400   -0.05886800
 C                  1.22866200   -2.65173300   -1.40412400
 H                  0.14289300   -2.74492900   -1.30157100
 H                  1.65219300   -3.66347900   -1.42351000
 H                  1.43635700   -2.18436400   -2.37390500
 C                  3.85935300   -1.62968400   -0.21738300
 H                  4.14881300   -1.32919600   -1.22962100
 H                  4.23430800   -2.64869000   -0.05899200
 H                  4.37688900   -0.97924700    0.49496900
 C                  3.10818200    1.65297400    1.66120200
 H                  2.37632500    1.62883600    2.47720700
 H                  3.67911500    2.58512100    1.75269200
 H                  3.80517100    0.82173100    1.81301600
 C                  1.53142100   -2.36099300    1.68071600
 H                  1.95531100   -1.75589100    2.49081400
 H                  1.90702700   -3.38520500    1.79350600
 H                  0.44405300   -2.38660600    1.81520400
 B                 -0.05895000    0.06626800   -0.06320200
 C                  3.47168100    1.63427600   -1.43080700
 H                  4.31980000    0.95771300   -1.29091100
 H                  3.87166000    2.65169200   -1.52329300
 H                  2.98818800    1.38246400   -2.38203900
 C                  1.03892100    3.01190800   -0.16914300
 H                  0.46254600    2.96616800   -1.09948500
 H                  1.60870100    3.94957300   -0.17367500
 H                  0.32525200    3.05653600    0.65976400
 B                 -1.88998000    0.00917700   -1.48375400
 H                 -1.11817400   -0.01751500   -2.38548600
 B                 -3.56149200    0.09472000    1.47179000
 H                 -4.13758200    0.12504900    2.51114100
 B                 -2.71363000    1.48595800   -0.94482600
 H                 -2.59006400    2.48156700   -1.57799700
 B                 -2.66543700   -1.38072600    0.93777000
 H                 -2.50966100   -2.37607100    1.56493400
 B                 -3.63012700    0.00810500   -1.43607600
 H                 -4.25429800   -0.02334800   -2.44721800
 B                 -2.67124800    1.53905000    0.85050900
 H                 -2.51962300    2.57063100    1.41680500
 B                 -1.82194200    0.09646200    1.43799500
 H                 -1.00802900    0.12358700    2.30212800
 B                 -4.12423200    0.95388500    0.00315500
 H                 -5.13176200    1.58570300    0.00830500
 B                 -4.12091300   -0.85299100    0.05677400
 H                 -5.12573700   -1.48768600    0.09915600
 B                 -2.70748300   -1.43389900   -0.85743400
 H                 -2.57931700   -2.46510500   -1.43050300
```

DippN3

```
 SCF Done: -631.913696
 N                 -0.00000400   -1.21217600    0.50988100
 N                 -0.00013300   -2.08787300   -0.35672600
 N                 -0.00047200   -3.00235500   -1.04087400
 C                  0.00007700    0.17648500    0.09657200
 C                 -1.23529400    0.84171300   -0.04579400
 C                  1.23541100    0.84159600   -0.04592200
 C                 -1.20222500    2.19626400   -0.40264200
 C                  1.20241300    2.19617800   -0.40284300
 C                  0.00012800    2.87032500   -0.59092900
 H                 -2.14260300    2.72726500   -0.52747000
 H                  2.14282400    2.72707100   -0.52788700
 H                  0.00017300    3.92123900   -0.86708100
 C                 -2.59821600    0.19189000    0.18550100
 H                 -3.32778400    1.00320700    0.06276000
 C                  2.59831200    0.19171100    0.18533400
 H                  3.32793300    1.00293600    0.06234900
 C                 -2.96650900   -0.87325800   -0.86584100
 H                 -2.39473900   -1.79696000   -0.74698700
 H                 -4.02646900   -1.13659300   -0.76966900
 H                 -2.80106900   -0.50023900   -1.88258800
 C                 -2.77178200   -0.34186600    1.61977500
 H                 -3.79920200   -0.69601700    1.76619300
 H                 -2.09112500   -1.17311500    1.82149700
 H                 -2.57377700    0.44508700    2.35579300
 C                  2.96635200   -0.87377700   -0.86577500
 H                  4.02624400   -1.13731700   -0.76951700
 H                  2.39433300   -1.79730100   -0.74664000
 H                  2.80100400   -0.50100500   -1.88261400
 C                  2.77197100   -0.34184400    1.61966600
 H                  2.09119300   -1.17296400    1.82158500
 H                  3.79933700   -0.69616100    1.76605900
 H                  2.57416600    0.44520600    2.35563500
```

TS1

```
 SCF Done: -1861.255107
 B                 -1.04210200    2.09621300    0.11236700
 H                 -1.93790500    1.38939000   -0.12807300
 B                  1.74724900    3.85985200    0.75427100
 H                  2.74883000    4.46225500    0.97766100
 B                 -0.35236100    3.13343000   -1.10928500
 H                 -0.82482800    3.15189300   -2.19865200
 B                  1.06981800    2.69957100    1.95918500
 H                  1.55308600    2.43291600    3.00930700
 B                 -1.11338600    3.79022800    0.39740800
 H                 -2.16618600    4.34349300    0.36473800
 B                  1.41347600    3.17413900   -0.88320000
 H                  2.12840200    3.24300800   -1.82715100
 B                  1.84003100    2.15886800    0.48285500
 H                  2.79041000    1.46800900    0.48994900
 B                  0.40229300    4.48815400   -0.22546500
 H                  0.43417300    5.58063300   -0.69687700
 B                  0.18428200    4.18847200    1.54493400
 H                  0.06441500    5.06645500    2.33955900
 B                 -0.69762300    2.64541400    1.73935300
 H                 -1.41093100    2.33095000    2.63446000
 C                  0.49764400    1.72699100   -0.56671300
 C                  0.32718100    1.46570100    0.98702400
 B                  0.53981900    0.16930600   -0.00218300
 N                  1.71474900   -0.73926100   -0.13757500
 Si                 2.56185200   -1.40915700    1.32067500
 Si                 2.58510700   -0.74836100   -1.73302000
 C                  4.38422200   -0.91276700    1.31441600
 H                  4.84674600   -1.31336000    2.22548200
 H                  4.94492100   -1.31052400    0.46321100
 H                  4.51171200    0.17407700    1.32933800
 C                  2.45805800   -3.29382900    1.36783200
 H                  3.07653400   -3.65635000    2.19898700
 H                  1.43221600   -3.63427300    1.54672900
 H                  2.81347500   -3.77129600    0.45059300
 C                  1.81461700   -0.78064800    2.92619400
 H                  1.89983100    0.30102800    3.04045400
 H                  0.76449200   -1.05430800    3.04945800
 H                  2.37239300   -1.25101000    3.74648400
 C                  3.43326900   -2.41753100   -1.99551200
 H                  3.92937200   -2.39084800   -2.97414300
 H                  4.20675300   -2.63348100   -1.25135300
 H                  2.72583600   -3.25148500   -2.00305800
 C                  1.33738600   -0.48375500   -3.12276300
 H                  0.56688600   -1.26072100   -3.15707600
 H                  0.84715800    0.49124500   -3.04708200
 H                  1.87313100   -0.51234500   -4.07972600
 C                  3.91848700    0.57492100   -1.88045700
 H                  4.43422500    0.41805400   -2.83742000
 H                  3.51013900    1.58694400   -1.88531500
 H                  4.66992900    0.51712500   -1.08822700
 N                 -0.83862600   -0.85136700   -0.20444700
 C                 -2.29853400   -0.77023500   -0.05371100
 C                 -2.84843400   -0.94394500    1.22524400
 C                 -3.07268100   -0.56397000   -1.20733700
 C                 -4.24442100   -0.94545200    1.32334400
 C                 -4.46251300   -0.58870200   -1.05734000
 C                 -5.04331600   -0.78250200    0.19388600
 H                 -4.71198300   -1.07416400    2.29374300
 H                 -5.09582000   -0.43971400   -1.92635400
 H                 -6.12536400   -0.79186500    0.29214100
 N                 -0.46976100   -2.01849700   -0.58529700
 N                  0.47165700   -2.64700600   -0.79801000
 C                 -1.95697000   -1.13843300    2.44209000
 H                 -1.02434700   -0.60617800    2.23811300
 C                 -2.42408000   -0.29085300   -2.55630100
 H                 -1.38284400   -0.01593000   -2.36796200
 C                 -2.42721900   -1.55510500   -3.43620000
 H                 -3.45354000   -1.86659300   -3.66416700
 H                 -1.90980000   -1.36531100   -4.38346500
 H                 -1.92925600   -2.39516400   -2.93838700
 C                 -3.06640200    0.90005800   -3.28605700
 H                 -3.06825100    1.79289800   -2.65310700
 H                 -2.49759800    1.12860700   -4.19430700
 H                 -4.09837500    0.68902100   -3.58863400
 C                 -2.53667800   -0.53557600    3.73001000
 H                 -1.78321000   -0.57073200    4.52443900
 H                 -2.82601700    0.50930800    3.58204500
 H                 -3.41208300   -1.09121100    4.08572600
 C                 -1.62537000   -2.63139000    2.63965600
 H                 -0.94562600   -2.76779100    3.48876800
```

H	-2.53840000	-3.20405200	2.84008500
H	-1.14921400	-3.06651700	1.75417600

Int1

SCF Done: -1861.268649

B	1.07392500	2.17027200	-0.13392300
H	2.02192900	1.49928400	0.00282300
B	-1.86205900	3.78706500	-0.49861400
H	-2.91203400	4.33297600	-0.62416400
B	0.40872400	3.11323300	1.18110500
H	0.95354800	3.11727400	2.23586800
B	-1.19780400	2.72930000	-1.80281700
H	-1.74509700	2.46603100	-2.82329600
B	1.01852200	3.88115600	-0.33894300
H	2.03489100	4.49900700	-0.34986200
B	-1.36718600	3.05991400	1.07988000
H	-2.01744100	3.04277500	2.07229600
B	-1.81052200	2.07516300	-0.30220300
H	-2.70443100	1.31609600	-0.28287300
B	-0.48798200	4.45841200	0.41711300
H	-0.55292800	5.52551800	0.94020200
B	-0.37968400	4.25113300	-1.37650400
H	-0.36838400	5.17026600	-2.13257800
B	0.58048900	2.77689100	-1.70326400
H	1.24546100	2.54473800	-2.65840800
C	-0.40593100	1.68582100	0.63524300
C	-0.31797000	1.50551400	-0.94087700
B	-0.33565000	0.15403800	-0.00415300
N	-1.54172300	-0.90772100	0.12004900
Si	-2.53417500	-1.35566700	-1.45475800
Si	-2.54410700	-0.89180900	1.74795300
C	-4.25925900	-0.60423800	-1.42998200
H	-4.74373600	-0.93426100	-2.35851300
H	-4.87197100	-0.97110900	-0.60116900
H	-4.26958100	0.48757700	-1.42036700
C	-2.71784400	-3.22509900	-1.54256200
H	-3.36270300	-3.44812600	-2.40323400
H	-1.75376300	-3.71511800	-1.70161200
H	-3.17442600	-3.66657800	-0.65464800
C	-1.59464200	-0.81449900	-2.97918600
H	-1.39184600	0.25497600	-3.03152200
H	-0.65186900	-1.35797700	-3.07380000
H	-2.21576600	-1.08571900	-3.84307700
C	-3.46223600	-2.53065700	1.86286800
H	-3.91152100	-2.58031800	2.86316000
H	-4.28085100	-2.61012600	1.14032000
H	-2.79914900	-3.38942500	1.74083500
C	-1.29399900	-0.72276700	3.13055300
H	-0.54165100	-1.51495800	3.11454100
H	-0.79079000	0.24766100	3.09666800
H	-1.83057900	-0.78816100	4.08544500
C	-3.82637000	0.47040400	1.90264200
H	-4.40127300	0.21838600	2.80513200
H	-3.38885800	1.45727700	2.05207600
H	-4.53206900	0.52773700	1.07171600
N	0.83385800	-0.83211300	0.12956700
C	2.28031900	-0.74409300	0.05607100
C	2.90363000	-0.85244900	-1.19960500
C	3.01228500	-0.59676600	1.24726100
C	4.30180200	-0.83837400	-1.23744900
C	4.40822800	-0.59562800	1.16075400
C	5.04941600	-0.71861800	-0.06874200
H	4.81274100	-0.92224800	-2.19125900
H	4.99933000	-0.48997700	2.06519800
H	6.13503500	-0.71217100	-0.11791000
N	0.41899600	-2.07639200	0.35397800
N	-0.81021000	-2.26469200	0.38043400
C	2.08334300	-1.00654500	-2.47048500
H	1.10727900	-0.55557800	-2.27721100
C	2.31219300	-0.44413100	2.58732000
H	1.28344000	-0.14330200	2.38003600
C	2.26836400	-1.79403700	3.32788200
H	3.28321400	-2.14232300	3.55487600
H	1.72253100	-1.70001900	4.27446500
H	1.78011800	-2.56175100	2.71777500
C	2.92560900	0.65789700	3.46589100
H	2.97156600	1.61041300	2.92824500
H	2.31201800	0.80198500	4.36267100
H	3.93760000	0.40302400	3.80082900
C	2.67620800	-0.26635100	-3.67890700
H	1.96859700	-0.29680900	-4.51530100
H	2.87607700	0.78301000	-3.44020100
H	3.60931000	-0.72491900	-4.02596900
C	1.86953000	-2.50030200	-2.78696300
H	1.24982700	-2.62557600	-3.68370100
H	2.83182000	-2.99174000	-2.97315000
H	1.38597600	-3.02002200	-1.95303400

TS2

SCF Done: -1861.263885

B	-0.99017500	2.14267900	-0.52599600
H	-1.80643300	1.42639300	-0.97071200
B	1.56962200	3.89854600	0.77256800
H	2.49783600	4.48828200	1.22929100
B	-0.11916500	3.29351600	-1.49340500
H	-0.38478800	3.39564800	-2.64752800
B	0.69518100	2.67897700	1.74743900
H	0.97590400	2.33645900	2.84923300
B	-1.17493500	3.82721000	-0.13200400
H	-2.21688800	4.36972100	-0.32344700
B	1.55463700	3.34554100	-0.93609400
H	2.45013800	3.49892500	-1.70208200
B	1.72092300	2.20565200	0.37784700
H	2.65881400	1.50583800	0.53103600
B	0.41328000	4.59961300	-0.39257600
H	0.50605500	5.72485300	-0.77158300
B	-0.12988400	4.17366400	1.27125800
H	-0.41739700	4.99420600	2.08526200
B	-0.99737200	2.61998500	1.18365200
H	-1.85870700	2.22119100	1.89449800
C	0.65343400	1.84564000	-0.93096600
C	0.18894200	1.52109100	0.58102300
B	0.33370300	0.05574300	0.06027800
N	1.55302900	-0.92234400	-0.04951000
Si	2.59882900	-1.30139900	1.52181300
Si	2.55793100	-0.83764100	-1.70657700
C	4.30037200	-0.50936900	1.44868500
H	4.80687400	-0.81490100	2.37418700
H	4.91183500	-0.86535000	0.61541700
H	4.27538800	0.58186500	1.42894800
C	2.80180600	-3.16446300	1.64469800
H	3.44474300	-3.36626600	2.51184200
H	1.84372400	-3.66528700	1.80735900
H	3.26930000	-3.61159000	0.76499100
C	1.64572100	-0.70367100	3.01270100
H	1.34487200	0.34299400	2.95671100
H	0.76130600	-1.32020800	3.18754000
H	2.30149700	-0.81564900	3.88589000
C	3.58492100	-2.41784200	-1.75646800
H	4.05778700	-2.45418300	-2.74666500
H	4.39178500	-2.44356700	-1.01766300
H	2.97321900	-3.31546700	-1.64137900
C	1.31795800	-0.84922000	-3.10638600
H	0.64244400	-1.70800300	-3.06744800
H	0.73783900	0.07607900	-3.11217600
H	1.88002900	-0.90971700	-4.04747100
C	3.72686600	0.61382800	-1.85226000
H	4.34860300	0.39137700	-2.73113500
H	3.20564400	1.55240100	-2.03270500
H	4.39712000	0.74508100	-1.00171600
N	-0.78906200	-0.89673500	-0.09105800
C	-2.23209500	-0.79022900	0.00769900
C	-2.82757200	-0.74233600	1.28115500
C	-2.98773300	-0.78217500	-1.17988000
C	-4.22465600	-0.68158300	1.34125500
C	-4.38018700	-0.74070800	-1.06557500
C	-4.99474500	-0.68628100	0.18202800
H	-4.71767900	-0.63664800	2.30622100
H	-4.98966500	-0.73693900	-1.96359700
H	-6.07833600	-0.64326300	0.25192400
N	-0.35045300	-2.15117100	-0.32304200
N	0.88319200	-2.28990900	-0.32037300
C	-1.99543000	-0.83780700	2.55185100
H	-1.03668600	-0.35120600	2.35596900
C	-2.32089300	-0.81216400	-2.54636500
H	-1.28540100	-0.49749700	-2.40684800
C	-2.30622600	-2.24372600	-3.11502000
H	-3.32876600	-2.60511000	-3.27777500
H	-1.78095900	-2.26912400	-4.07749900
H	-1.80961300	-2.93715400	-2.42870900
C	-2.94760800	0.17639200	-3.54318600
H	-2.98314100	1.18827100	-3.12734100
H	-2.34826000	0.20521900	-4.46034800
H	-3.96507500	-0.11481000	-3.82812100
C	-2.61129300	-0.12051500	3.76211800
H	-1.88839500	-0.10203100	4.58530400
H	-2.87916900	0.91245200	3.52152400
H	-3.50737600	-0.63412400	4.12900500
C	-1.73038100	-2.32151700	2.88373700
H	-1.12722200	-2.41748200	3.79498400
H	-2.67817600	-2.84492000	3.05485000
H	-1.21248000	-2.83477800	2.06664700

2a

SCF Done: -1861.375415

B	-0.19216100	1.79563200	-1.17062400

H	0.56082300	2.23966000	-0.38053000
B	-2.51842300	0.30363800	-3.11647800
H	-3.35664400	-0.24822200	-3.75424500
B	-1.58036600	2.69489800	-1.81717800
H	-1.74345000	3.80889900	-1.44672000
B	-1.10833300	-0.57562900	-2.49231200
H	-0.90445900	-1.73875300	-2.58365800
B	-0.29327800	2.15346400	-2.90861200
H	0.46514000	2.92431900	-3.40186700
B	-2.93918800	1.56946900	-1.94683400
H	-4.04174300	1.90146900	-1.66876700
B	-2.37553100	-0.00809500	-1.37430800
H	-3.03717800	-0.71966300	-0.70669900
B	-2.00963600	1.99939900	-3.39762300
H	-2.49382100	2.67729000	-4.24634600
B	-0.87650000	0.67281500	-3.73500100
H	-0.52686100	0.37499500	-4.83198700
B	0.24881000	0.55740800	-2.36577600
H	1.36351900	0.17144000	-2.37655500
C	-1.79583600	1.43985200	-0.66679100
C	-0.71016200	0.16038200	-1.00579800
B	-0.08692300	-0.72809200	0.14673500
N	-0.62645700	-1.84792200	0.86719000
Si	-2.00030800	-3.05240000	0.83332800
Si	-2.36264400	1.94220900	1.12711700
C	-3.54194200	-2.33688000	1.64707200
H	-4.34865400	-3.07810700	1.58118200
H	-3.36721100	-2.13834700	2.71054500
H	-3.90160800	-1.41589100	1.18042300
C	-1.42202400	-4.53653300	1.82819700
H	-2.19797400	-5.31222900	1.80018400
H	-0.50049000	-4.95989800	1.41573200
H	-1.22638400	-4.27731900	2.87216600
C	-2.32979200	-3.60840200	-0.93156900
H	-2.80992400	-2.85530900	-1.55883300
H	-1.40110900	-3.91694200	-1.42400000
H	-2.98941900	-4.48502300	-0.89211900
C	-1.71760800	0.80446000	2.48315200
H	-2.05478000	1.23475100	3.43557100
H	-2.13029000	-0.20163600	2.41053900
H	-0.63053700	0.71617300	2.53397100
C	-1.74620100	3.69300900	1.44063900
H	-0.67511900	3.78500200	1.23715300
H	-2.26970800	4.42751300	0.82143700
H	-1.91280800	3.95536300	2.49266700
C	-4.24483600	1.87555400	1.15612500
H	-4.59763500	2.02863300	2.18346500
H	-4.69167200	2.64975400	0.52531300
H	-4.62199100	0.90592900	0.81335500
N	1.21561100	-0.57817900	0.71960300
C	2.43623400	0.07012800	0.31083900
C	3.24286900	-0.59209800	-0.63864600
C	2.81394900	1.29126900	0.89895600
C	4.44248500	0.02300800	-1.01206800
C	4.02200900	1.86557300	0.48866800
C	4.82702300	1.24131200	-0.45997000
H	5.08447000	-0.45784900	-1.74257700
H	4.33930000	2.81004000	0.91711600
H	5.76103100	1.70448700	-0.76683200
N	1.36565700	-1.51388300	1.72778400
N	0.32442900	-2.22162200	1.81856000
C	2.87107500	-1.96146800	-1.19878000
H	1.78447000	-2.07594300	-1.12537400
C	1.96717100	1.93627200	1.98662000
H	0.92606400	1.67543700	1.77936900
C	2.32541900	1.34939200	3.36681900
H	3.36453600	1.58728600	3.62417000
H	1.67708600	1.77374100	4.14330800
H	2.21469000	0.26139400	3.37597100
C	2.05119500	3.46960700	2.00764900
H	1.84794500	3.89808200	1.01989500
H	1.31804100	3.87250100	2.71504400
H	3.03723500	3.82054400	2.33236900
C	3.23755900	-2.14094200	-2.68114600
H	2.82983200	-3.08909200	-3.04914200
H	2.83249100	-1.33313900	-3.29842100
H	4.32253000	-2.17626400	-2.83249300
C	3.51328400	-3.07207000	-0.34132800
H	3.21321500	-4.06090900	-0.70872600
H	4.60696500	-3.00817200	-0.39216500
H	3.21516700	-2.98516600	0.70712300

TS3-Dipp

SCF Done: -1861.268584

Si	-0.10480200	2.91626200	-0.40440400
Si	0.44817100	1.33716300	2.22549100
N	-0.08395700	1.38549400	0.52207300
C	-2.18522000	0.20794700	-0.88516300
C	-2.03739100	-0.67243700	0.42841800
C	-0.11651600	2.58237000	-2.26590200
H	0.78260900	2.08313400	-2.64134100
H	-0.18538600	3.54871300	-2.78141700
H	-0.98558900	1.99090600	-2.56902000
C	-1.61817300	3.97809100	-0.02274000
H	-2.54025100	3.54480500	-0.41869500
H	-1.48358400	4.96313500	-0.48840500
H	-1.75502600	4.13358200	1.05195600
C	2.32207900	1.50532400	2.38196400
H	2.83475700	0.68123100	1.87601800
H	2.59911800	1.46830800	3.44343000
H	2.70108700	2.44722300	1.97571800
C	1.41028900	3.96739700	0.00774800
H	1.42929800	4.28641800	1.05519600
H	1.37263600	4.87618800	-0.60607700
H	2.35314000	3.45991600	-0.20782900
B	-0.78970500	0.27543900	-0.05052600
C	-0.37501200	2.73569800	3.19470400
H	-0.08678000	3.73203100	2.84210900
H	-0.08918100	2.66681400	4.25177400
H	-1.46655100	2.66147900	3.13841200
C	-0.02701400	-0.30171100	3.01770400
H	-1.10633400	-0.47281200	3.03375800
H	0.33354800	-0.30382200	4.05404800
H	0.44519500	-1.14252000	2.50347100
B	-3.01550100	0.76980200	0.56844100
H	-2.56919400	1.68193900	1.15998200
B	-3.88466300	-1.97206500	-1.21117900
H	-4.17032100	-2.94225600	-1.83737800
B	-3.49493900	-0.70922700	1.37148300
H	-3.43808300	-0.77089100	2.55540300
B	-3.28180900	-0.47317300	-2.03003400
H	-3.08271300	-0.34031900	-3.19248300
B	-4.66219400	0.28292800	0.41035900
H	-5.50177300	0.93458700	0.94465000
B	-3.01542200	-2.10244100	0.37077700
H	-2.63671500	-3.10758600	0.87582100
B	-2.23154300	-1.51049900	-1.08195700
H	-1.30960900	-2.06893900	-1.54657500
B	-4.69088400	-1.49273400	0.30279100
H	-5.59020500	-2.12899300	0.75287400
B	-4.85500600	-0.47762200	-1.18564500
H	-5.87305200	-0.39098800	-1.79557500
B	-3.75716200	0.92076400	-1.02061500
H	-3.88926000	1.99225900	-1.51281600
N	0.60682300	-0.73418700	-0.91517700
N	0.53311700	-0.74661700	-2.16225400
N	0.33609200	-0.69646900	-3.27762300
C	1.85447000	-1.23874200	-0.32701500
C	3.07423600	-0.57010700	-0.55983500
C	1.76319300	-2.39459500	0.47793800
C	4.21653900	-1.08400400	0.06858000
C	2.93304500	-2.84063800	1.10139000
C	4.14841900	-2.19293200	0.90357300
H	5.17216200	-0.59696800	-0.09165600
H	2.89261900	-3.71781100	1.73716200
H	5.04631100	-2.55997100	1.39287000
C	3.20769000	0.62560800	-1.49305500
H	2.23201000	1.11446600	-1.55212900
C	0.47509400	-3.19648100	0.58224700
H	-0.35430400	-2.49394600	0.54456000
C	0.32445400	-3.99616200	1.88447100
H	1.02371000	-4.83907100	1.93567100
H	0.47717900	-3.36953800	2.76886200
H	-0.68800100	-4.40937600	1.93616200
C	0.35502900	-4.13661600	-0.63530600
H	-0.61439100	-4.64619900	-0.62621400
H	0.43821600	-3.59235200	-1.58153100
H	1.14729500	-4.89433000	-0.61311300
C	3.62312100	0.16626700	-2.90840600
H	3.64949400	1.02018500	-3.59482900
H	4.62530500	-0.27679700	-2.87841200
H	2.94663200	-0.58321100	-3.32721100
C	4.21046700	1.67944900	-0.98987200
H	4.12813000	2.58695700	-1.59740100
H	4.03109500	1.94264900	0.05544900
H	5.24442100	1.32805200	-1.07772400

Int2-Dipp

SCF Done: -1861.269103

Si	-0.06520300	2.90853900	-0.39397500
Si	0.48558600	1.32663600	2.21696500
N	-0.03324300	1.37219000	0.51389500
C	-2.14308500	0.20938700	-0.88528100
C	-2.00873500	-0.66343300	0.42387400
C	-0.09679700	2.59450200	-2.26114400
H	0.79954300	2.10663400	-2.65833500

H	-0.17932500	3.56683800	-2.76311800
H	-0.96798200	2.00314600	-2.55877400
C	-1.56654500	3.98656100	-0.00444800
H	-2.49446200	3.57126900	-0.40521500
H	-1.41505000	4.97288300	-0.46245300
H	-1.70307700	4.13660400	1.07090900
C	2.36087400	1.48491800	2.38852600
H	2.87324800	0.65618500	1.88960300
H	2.63130600	1.45198700	3.45183000
H	2.74662900	2.42322600	1.97970900
C	1.45307500	3.95990700	0.01155800
H	1.47601400	4.27520900	1.06012900
H	1.41201400	4.87106000	-0.59852400
H	2.39568900	3.45446900	-0.20830300
B	-0.71031900	0.23499400	-0.08546300
C	-0.32610700	2.72922400	3.19167100
H	-0.03065300	3.72503500	2.84360600
H	-0.03812900	2.65301900	4.24773200
H	-1.41826800	2.66408100	3.13796200
C	-0.00106800	-0.30628600	3.01650800
H	-1.08181100	-0.46736600	3.03288400
H	0.35960500	-0.30684700	4.05281200
H	0.46294800	-1.15399200	2.50631700
B	-2.94830900	0.79872400	0.56680000
H	-2.47918100	1.70005100	1.15496300
B	-3.90804700	-1.92527500	-1.19218500
H	-4.22352000	-2.89045300	-1.81213200
B	-3.46121000	-0.66104900	1.37989300
H	-3.39833400	-0.71928700	2.56367900
B	-3.26830700	-0.44802900	-2.02188600
H	-3.07385600	-0.32609600	-3.18676900
B	-4.60891500	0.35812900	0.42341300
H	-5.42762900	1.03426200	0.95987800
B	-3.03022900	-2.07076900	0.38313500
H	-2.67507500	-3.08384900	0.88997700
B	-2.24334200	-1.50785400	-1.07541400
H	-1.34131900	-2.09598300	-1.54531100
B	-4.68848400	-1.41649900	0.32491900
H	-5.60124100	-2.02502000	0.78649500
B	-4.83498300	-0.40441700	-1.16693300
H	-5.85446000	-0.29097100	-1.77034800
B	-3.69651300	0.96325700	-1.01675000
H	-3.80434900	2.03420300	-1.51595700
N	0.55004800	-0.71119500	-0.89221500
N	0.46885600	-0.74195000	-2.14426700
N	0.29064900	-0.70820600	-3.26100700
C	1.80130700	-1.24532000	-0.32071900
C	3.02558300	-0.59095600	-0.57042500
C	1.70813600	-2.41280800	0.46700600
C	4.17114800	-1.12686100	0.03290500
C	2.88337400	-2.87948700	1.06531000
C	4.10275100	-2.24397700	0.85645400
H	5.13001100	-0.65066500	-0.13897700
H	2.84241900	-3.76492500	1.68928100
H	5.00381700	-2.62802600	1.32641800
C	3.16585700	0.60775700	-1.49842400
H	2.19784800	1.11161400	-1.54368200
C	0.41955400	-3.21174100	0.58114400
H	-0.40907400	-2.50994800	0.54395600
C	0.27184100	-4.00315400	1.88909500
H	0.96815700	-4.84820700	1.94367200
H	0.42838500	-3.37108900	2.76871500
H	-0.74187400	-4.41248300	1.94582600
C	0.29715600	-4.15983500	-0.63005100
H	-0.67386000	-4.66608200	-0.61712800
H	0.38185500	-3.62281200	-1.58041600
H	1.08723900	-4.91965800	-0.60267800
C	3.56162400	0.14896500	-2.91987000
H	3.58866700	1.00525500	-3.60317200
H	4.56000100	-0.30312200	-2.90215400
H	2.87539000	-0.59336800	-3.33566300
C	4.19084000	1.64323400	-1.00188500
H	4.11542400	2.55420500	-1.60489500
H	4.02732500	1.90474700	0.04618900
H	5.21806200	1.27629800	-1.10371200

TS4-Dipp

SCF Done: -1861.250097

Si	0.54336900	2.60645000	-0.89632900
Si	-0.54156300	2.11520100	1.94356100
N	-0.13458300	1.47538000	0.31390700
C	-2.01175000	-0.49442000	-1.52867400
C	-2.02306800	-0.43485100	0.07068700
C	0.28474100	1.94307900	-2.64326900
H	0.86752800	1.05715100	-2.90708500
H	0.56302800	2.73425800	-3.35080200
H	-0.76905500	1.69645600	-2.80382200
C	-0.38274500	4.25164200	-0.83487000
H	-1.46397700	4.11650600	-0.93917700
H	-0.04011200	4.87010100	-1.67454600
H	-0.19597200	4.81942700	0.08210900
C	0.93857800	3.10709000	2.57593100
H	1.87131000	2.53816600	2.49489000
H	0.77590200	3.33579000	3.63696400
H	1.07413000	4.06095800	2.05661900
C	2.36313200	2.95541800	-0.53014000
H	2.46687700	3.76306900	0.20102500
H	2.89123300	3.26288800	-1.44010800
H	2.86090700	2.07306700	-0.11780300
B	-0.56145400	0.17539900	-0.05999400
C	-2.06135800	3.23268500	1.97603100
H	-1.81817900	4.25578300	1.67308200
H	-2.45434600	3.27353400	2.99976200
H	-2.86217200	2.86807600	1.32965300
C	-0.73214700	0.65617900	3.11450700
H	-1.50545700	-0.05803200	2.82109400
H	-0.98123100	1.02222500	4.11795700
H	0.22340600	0.12689200	3.18289000
B	-2.91298000	0.77898600	-0.80014500
H	-2.48428000	1.87853400	-0.85201000
B	-3.76325800	-2.47982600	-0.59458700
H	-4.02344000	-3.63959500	-0.52479500
B	-3.54561800	0.08834000	0.70719700
H	-3.57855300	0.74416200	1.69375100
B	-3.05241300	-1.76299800	-2.08600000
H	-2.75591600	-2.37172800	-3.06410400
B	-4.58404300	0.28540700	-0.74373200
H	-5.44233000	1.10996700	-0.77241400
B	-3.04770300	-1.61862200	0.80630800
H	-2.73294500	-2.09474800	1.85004000
B	-2.10008600	-1.97442600	-0.64018100
H	-1.15919900	-2.68255200	-0.59441100
B	-4.68226800	-1.19840400	0.24300000
H	-5.63687500	-1.43965200	0.91306900
B	-4.69446700	-1.30127500	-1.55525700
H	-5.66040400	-1.62006800	-2.17454000
B	-3.55594300	-0.07104200	-2.17921200
H	-3.61322100	0.49015100	-3.22558800
N	0.62548700	-0.76582200	-0.58414400
N	0.45302700	-1.35851200	-1.70967200
N	0.55394500	-1.91637600	-2.68607500
C	1.87953300	-1.05496000	0.14505200
C	3.12961700	-0.66194400	-0.38767600
C	1.76003600	-1.68143500	1.40839100
C	4.23820500	-0.74360500	0.46481400
C	2.90181400	-1.71267500	2.21735800
C	4.12079700	-1.21723500	1.76644900
H	5.20972700	-0.43217200	0.09792700
H	2.83975400	-2.15574600	3.20456300
H	4.99184200	-1.24345300	2.41519300
C	3.35461800	-0.32955400	-1.85950200
H	2.43986400	0.10924000	-2.26426800
C	0.51954700	-2.47089700	1.80979300
H	-0.37047000	-1.93427500	1.48315000
C	0.36718700	-2.73198300	3.31584900
H	1.11501600	-3.44500300	3.68152700
H	0.44386000	-1.81854000	3.91059100
H	-0.61853900	-3.16994800	3.50227900
C	0.55746300	-3.82674200	1.06646500
H	-0.37002500	-4.38093300	1.24359400
H	0.67125400	-3.70312900	-0.01427500
H	1.40034700	-4.42966000	1.42467200
C	3.66277900	-1.64283100	-2.62078400
H	3.72899100	-1.45426900	-3.69776300
H	4.62433000	-2.04589400	-2.28329800
H	2.91171300	-2.42002900	-2.45700900
C	4.49702800	0.66440700	-2.13728900
H	4.46306100	0.96783000	-3.18903700
H	4.43204500	1.56214100	-1.52203700
H	5.47727700	0.20534900	-1.96859700

Int3-Dipp

SCF Done: -1861.341777

Si	0.68385200	1.92796100	-2.02133600
Si	-0.85733500	3.06907700	0.39350400
N	-0.23719900	1.66993700	-0.51551400
C	-1.72001200	-1.93944900	0.38722500
C	-1.91498300	-0.39793900	-0.03459100
C	0.23795500	0.56158600	-3.24289700
H	0.44986700	-0.44285400	-2.87768600
H	0.79238900	0.69779600	-4.17928900
H	-0.83201000	0.61480800	-3.47668200
C	0.16017200	3.51587100	-2.90868600
H	-0.89081000	3.46950200	-3.21404000
H	0.76182400	3.58778900	-3.82435400
H	0.31242800	4.44190400	-2.34870800

C	0.52098700	4.35194900	0.58863100
H	1.38782700	3.89824000	1.08338700
H	0.16354600	5.16592400	1.23245000
H	0.86513600	4.80083900	-0.34691100
C	2.53658300	2.01171900	-1.67577300
H	2.75939700	2.86030800	-1.01761500
H	3.10381200	2.15186900	-2.60470300
H	2.90573400	1.10693400	-1.18414300
B	-0.44346700	0.29929800	-0.10720700
C	-2.35014400	3.83991200	-0.46859400
H	-2.07196800	4.34939900	-1.39588500
H	-2.82761200	4.57925900	0.18674600
H	-3.09866000	3.07936400	-0.71574100
C	-1.33704500	2.61799800	2.17045300
H	-1.70493500	1.60256700	2.31752900
H	-2.13378600	3.29724500	2.49759600
H	-0.48623900	2.76641700	2.84324500
B	-2.06411500	-1.63198100	-1.26217400
H	-1.18022700	-1.74376100	-2.03211700
B	-4.14940600	-1.23936300	1.39642200
H	-4.85999700	-1.08910300	2.33634900
B	-3.19644000	-0.27865300	-1.16562900
H	-3.13194300	0.56820400	-1.99234300
B	-2.98800400	-2.59207900	1.31866300
H	-2.70746300	-3.37675500	2.15966400
B	-3.77928800	-1.96139500	-1.39700300
H	-4.22598200	-2.32550000	-2.43577800
B	-3.42826100	0.15739400	0.53937100
H	-3.53958200	1.29159400	0.85184000
B	-2.43260100	-0.90688200	1.54568800
H	-1.75502700	-0.58925500	2.45874400
B	-4.63775300	-0.85274300	-0.28530400
H	-5.71745100	-0.42586000	-0.53722600
B	-4.36239500	-2.55338100	0.19360200
H	-5.23833600	-3.35116200	0.28306000
B	-2.75823300	-3.03610700	-0.40098800
H	-2.32752600	-4.10653500	-0.66822800
N	0.50760700	-0.68303800	0.18450800
N	-0.30197100	-2.21266900	0.65655100
N	0.24781000	-3.14225200	1.07668500
C	1.86914700	-0.80852500	0.44600000
C	2.67280600	-1.61356000	-0.40439800
C	2.42601900	-0.22505900	1.61990000
C	4.02817100	-1.77117700	-0.09424200
C	3.78542200	-0.40579500	1.87484200
C	4.58620600	-1.16862100	1.02621900
H	4.65386600	-2.37707900	-0.74317500
H	4.22904300	0.04364000	2.75731800
H	5.64219600	-1.29937800	1.24727300
C	2.12475100	-2.29199400	-1.65158800
H	1.06683200	-2.03078500	-1.73529700
C	1.55791900	0.53018800	2.61289200
H	0.69160400	0.90993100	2.07243400
C	2.25826400	1.74155300	3.24749200
H	3.06555500	1.44236200	3.92548100
H	2.68457900	2.40139600	2.48413800
H	1.54220800	2.32054000	3.84130400
C	1.03931900	-0.43269600	3.69975300
H	0.34138400	0.08226700	4.37072200
H	0.52275100	-1.29099700	3.25940000
H	1.87136500	-0.81884300	4.30034800
C	2.21671400	-3.82711800	-1.55793700
H	1.76727800	-4.28794700	-2.44549400
H	3.26072800	-4.15734000	-1.50369300
H	1.70040500	-4.20538000	-0.67130000
C	2.84059600	-1.79024600	-2.92149800
H	2.35910800	-2.19969200	-3.81715000
H	2.82085000	-0.69890900	-2.99093000
H	3.88958000	-2.10746100	-2.93291300

BnN3

SCF Done: -435.271127

N	-2.23576000	-0.78546900	-0.09930000
N	-2.38774500	0.38333900	-0.46997600
N	-2.60976900	1.42267400	-0.88928100
C	-1.17798200	-0.98049300	0.93914800
H	-1.49226900	-0.49651800	1.87336700
H	-1.16757400	-2.05903500	1.10817100
C	0.17988200	-0.47892800	0.50657800
C	0.63115200	0.78334900	0.90833100
C	0.97767800	-1.24604000	-0.35248900
C	1.86306800	1.27003400	0.46632700
H	0.01733900	1.38685900	1.57331300
C	2.20801600	-0.76301500	-0.79454800
H	0.62676900	-2.22285800	-0.67692000
C	2.65335600	0.49711100	-0.38501800
H	2.20375600	2.25052100	0.78760000
H	2.82075800	-1.36832400	-1.45710200
H	3.61311100	0.87344000	-0.72851400

TS1-Bn

SCF Done: -1664.60913

B	1.09717500	2.10728200	0.13022600
H	2.03995300	1.43343700	0.32807100
B	-1.82897900	3.70956900	-0.37002200
H	-2.87089700	4.25796700	-0.54109400
B	0.30235900	2.93514200	1.44041000
H	0.75474700	2.86090500	2.53555900
B	-1.04476800	2.75826600	-1.69273000
H	-1.48794700	2.56602100	-2.77765100
B	1.02770400	3.82569600	0.03825900
H	2.02919400	4.45676900	0.15555200
B	-1.45739100	2.85784500	1.18129800
H	-2.18712000	2.75113100	2.11063100
B	-1.78715700	1.98782500	-0.30608300
H	-2.66850500	1.21890000	-0.42785100
B	-0.55073400	4.32094100	0.70371900
H	-0.67550000	5.34360500	1.29962500
B	-0.28598100	4.25391300	-1.08375100
H	-0.22950000	5.22834800	-1.76451900
B	0.72289700	2.82002400	-1.43530600
H	1.46896700	2.66719300	-2.34792200
C	-0.41677500	1.54236600	0.71073400
C	-0.22769900	1.49662000	-0.85696100
B	-0.31915200	0.06845600	-0.06578000
N	-1.40170300	-0.96092200	0.04151300
Si	-2.01783100	-1.66416800	-1.51340100
Si	-2.35842500	-1.12927300	1.58013500
C	-3.85702200	-1.29451300	-1.70624900
H	-4.18689600	-1.65928100	-2.68713000
H	-4.47468200	-1.78768200	-0.94864800
H	-4.05834600	-0.21934300	-1.66483900
C	-1.74657900	-3.53062700	-1.58290900
H	-2.23539400	-3.92354000	-2.48367300
H	-0.68280700	-3.78323900	-1.64939800
H	-2.16048200	-4.05786200	-0.71944600
C	-1.08702500	-0.90144800	-2.96266600
H	-1.28515400	0.16779100	-3.07207800
H	-0.00291000	-1.03447700	-2.88358100
H	-1.41207800	-1.40296300	-3.88294000
C	-3.01064900	-2.89848300	1.69671100
H	-3.54764900	-3.00069600	2.64825300
H	-3.72183700	-3.14562900	0.90092300
H	-2.20858300	-3.64187300	1.68357900
C	-1.23277600	-0.78222700	3.05012900
H	-0.38677600	-1.47601400	3.10050900
H	-0.84109000	0.23841000	3.03005500
H	-1.81228100	-0.90076900	3.97426000
C	-3.86080100	0.00505500	1.66833100
H	-4.45580200	-0.30386700	2.53815500
H	-3.59480700	1.05517800	1.80115100
H	-4.50084400	-0.07493700	0.78530200
N	1.07613900	-0.77561600	-0.18808700
N	0.95796100	-1.96334300	0.32144300
N	0.07694600	-2.59505600	0.73454900
C	2.25001100	-0.61146600	-1.06789800
H	2.17458700	-1.34021800	-1.88480700
H	2.14620700	0.37525800	-1.51779800
C	3.58582700	-0.75905100	-0.36579300
C	3.73253800	-0.58028700	1.01358900
C	4.71434900	-1.05846400	-1.13864600
C	4.99039700	-0.69538600	1.60680600
H	2.86657500	-0.34746100	1.62508500
C	5.97103800	-1.16773200	-0.54533200
H	4.60881300	-1.20717600	-2.21138000
C	6.11230000	-0.98692300	0.83264500
H	5.09023400	-0.55384200	2.68114500
H	6.83793300	-1.40043600	-1.15760600
H	7.09019700	-1.07628500	1.29735900

Int1-Bn

SCF Done: -1664.620048

B	1.45961000	1.87360600	-0.43849900
H	2.30137700	1.12936500	-0.75837000
B	-1.14839100	3.74175100	0.57868100
H	-2.08703600	4.37636000	0.94226300
B	1.39048700	2.47830300	1.19790300
H	2.22895200	2.15516500	1.97323200
B	-1.09342200	3.02196600	-1.07890700
H	-1.95462800	3.08125200	-1.89466400
B	1.61836600	3.57576500	-0.22294500
H	2.66660300	4.09525900	-0.43686500
B	-0.31480300	2.58226700	1.68905300
H	-0.63197300	2.33806100	2.80695800
B	-1.30913800	2.04084000	0.35603200
H	-2.25992700	1.37595400	0.51247200

B	0.52640600	4.04090900	1.10546900
H	0.79503100	4.93124500	1.84829700
B	0.04601900	4.31168600	-0.61792100
H	-0.03144800	5.39621200	-1.10177800
B	0.61500800	2.91152700	-1.57488100
H	0.91259900	2.89502900	-2.72428200
C	0.23677800	1.30930400	0.63802000
C	-0.20193200	1.55584200	-0.87013500
B	-0.14938800	0.00762500	-0.33491200
N	-1.40296000	-0.94882600	-0.02638900
Si	-2.75271800	-0.91023100	-1.37057100
Si	-1.83450900	-1.26118400	1.80415900
C	-3.95552600	0.52048900	-1.19855900
H	-4.76211200	0.32354100	-1.91839400
H	-4.41379500	0.58152900	-0.20698400
H	-3.51957100	1.49152700	-1.43746700
C	-3.75428700	-2.49856400	-1.28007000
H	-4.29987800	-2.59206000	-2.22824800
H	-3.13295100	-3.38707500	-1.15502700
H	-4.50024500	-2.47426700	-0.47999900
C	-1.80676400	-0.80139700	-2.98797300
H	-1.26662600	0.14541600	-3.07684600
H	-1.09297100	-1.62293100	-3.10087400
H	-2.52215400	-0.86285700	-3.81722900
C	-2.77891800	-2.88313200	1.94002800
H	-2.79382500	-3.15425400	3.00400200
H	-3.82024200	-2.78916600	1.61859800
H	-2.31226900	-3.69832900	1.38495500
C	-0.17654000	-1.38715600	2.67148600
H	0.49025000	-2.09012100	2.16205100
H	0.32315000	-0.41736600	2.73697400
H	-0.33778300	-1.75982800	3.69043100
C	-2.94045400	0.04794600	2.57366800
H	-3.28200500	-0.37317800	3.52932500
H	-2.43784200	0.99305900	2.78018000
H	-3.83306100	0.25262200	1.97444200
N	0.79444600	-1.06318200	-0.89177700
N	0.29823500	-2.28654400	-0.77629500
N	-0.85784800	-2.38260900	-0.32400400
C	2.07431100	-1.06342400	-1.60940500
H	2.04153200	-1.91366700	-2.29722600
H	2.11432000	-0.16212600	-2.22484400
C	3.30474400	-1.18087500	-0.72682000
C	3.24760900	-1.02605100	0.66062200
C	4.54058800	-1.44048200	-1.33125600
C	4.40669300	-1.12907400	1.43232100
H	2.29857100	-0.81201300	1.13910900
C	5.69920100	-1.54220400	-0.56276900
H	4.59578900	-1.56452400	-2.41117800
C	5.63514800	-1.38746200	0.82448700
H	4.34602800	-1.00176000	2.50978800
H	6.65134600	-1.74465700	-1.04595500
H	6.53717400	-1.46725400	1.42494200

2-Bn

SCF Done: -1664.729206

B	-0.99690900	-1.76479600	-0.01721600
H	-1.53426300	-1.68537100	1.02995400
B	0.88316800	-1.84014000	-2.81816000
H	1.58613100	-1.83731200	-3.77726900
B	-0.22352600	-3.26085000	-0.57459500
H	-0.28009200	-4.21219700	0.12986400
B	0.07195600	-0.36120300	-2.26726700
H	0.18782800	0.72046000	-2.73078900
B	-1.58484000	-2.57720700	-1.48302400
H	-2.65067000	-3.10359100	-1.48587500
B	1.28062100	-2.81415400	-1.38966200
H	2.26951500	-3.45256300	-1.24913300
B	1.41554300	-1.05082100	-1.32084300
H	2.43568600	-0.49846000	-1.10852200
B	-0.15575600	-3.22377600	-2.35082300
H	-0.19507900	-4.23310500	-2.97860900
B	-0.90231900	-1.70623900	-2.89241500
H	-1.48930700	-1.59992600	-3.92116300
B	-1.43889600	-0.80885600	-1.45365400
H	-2.32720200	-0.03383500	-1.37541500
C	0.70929300	-1.92538900	-0.03168500
C	-0.01134700	-0.46507500	-0.56739600
B	0.08457600	0.88699500	0.25491900
N	1.02856200	1.96484600	0.13632500
Si	2.57979500	2.47008300	-0.68761700
Si	1.66271800	-2.07996300	1.65357800
C	3.99829300	1.61784700	0.21401500
H	4.95832800	1.94023500	-0.20783100
H	3.99380500	1.89216500	1.27541400
H	3.95473700	0.52685600	0.14499500
C	2.71575200	4.33092500	-0.46942100
H	3.59989400	4.69404900	-1.00875400
H	1.83676300	4.84233200	-0.87629800
H	2.80581200	4.61461300	0.58204700
C	2.58811300	2.10875700	-2.53171900
H	2.62430800	1.05048700	-2.79339900
H	1.71821800	2.55326200	-3.02639600
H	3.48342500	2.58986600	-2.94767200
C	1.68336700	-0.46864100	2.62999800
H	2.27119400	-0.65173100	3.53898700
H	2.15777600	0.35757200	2.09429300
H	0.68822700	-0.14223600	2.94393800
C	0.79282600	-3.40174800	2.67361100
H	-0.27837100	-3.19379600	2.76976900
H	0.90184600	-4.39613900	2.23057200
H	1.22368100	-3.42991200	3.68195000
C	3.43389400	-2.58436100	1.26220200
H	4.02821900	-2.57697700	2.18409000
H	3.48705800	-3.58957900	0.83355100
H	3.90457600	-1.89354100	0.55459500
N	-0.78615700	1.35176600	1.28856900
N	-0.35524100	2.58226600	1.72056500
N	0.67316000	2.93299700	1.07593800
C	-1.99846700	0.87681000	1.95674200
H	-1.82521300	-0.12978300	2.34378000
H	-2.13131500	1.54023100	2.81713800
C	-3.22410400	0.90434100	1.06766300
C	-3.49056000	2.02536900	0.27245500
C	-4.09814900	-0.18452800	1.02851200
C	-4.60601600	2.04630500	-0.56288000
H	-2.81250400	2.87436900	0.29942400
C	-5.21760600	-0.16429600	0.19457600
H	-3.89295000	-1.06267800	1.63558300
C	-5.47040700	0.94892000	-0.60737200
H	-4.79913800	2.91719300	-1.18344700
H	-5.88157400	-1.02363500	0.16167100
H	-6.33416200	0.96168100	-1.26636000

TS3

SCF Done: -1664.621982

Si	-0.18164800	2.01585900	1.66119200
Si	0.52843800	2.43766300	-1.36491800
N	0.40723100	1.40858400	0.08764100
C	1.83662200	-0.97460800	0.75140400
C	1.95592500	-0.72151500	-0.82363400
C	0.38307500	0.86949500	3.04524600
H	-0.05290800	-0.13228000	2.97952900
H	0.07677200	1.29859600	4.00746400
H	1.47139400	0.75316300	3.05903700
C	0.51985400	3.73718000	1.99191400
H	1.61546400	3.72551600	1.98776300
H	0.19344400	4.07732800	2.98253700
H	0.18691300	4.48473200	1.26398300
C	-1.05652600	3.45965200	-1.52538800
H	-1.94213000	2.81635500	-1.60058400
H	-1.01067700	4.06664200	-2.43795900
H	-1.21294200	4.14406900	-0.68500500
C	-2.06938000	2.10177600	1.67810500
H	-2.46152300	2.76381100	0.89865800
H	-2.42042700	2.48341900	2.64508500
H	-2.51221700	1.11125200	1.53167500
B	0.83205000	0.05674900	0.02033000
C	2.02031800	3.58534100	-1.26214600
H	1.97432200	4.26329900	-0.40443000
H	2.08586300	4.19713200	-2.17081800
H	2.94637500	3.00551700	-1.18361600
C	0.70570800	1.39515800	-2.92811900
H	1.70091700	0.94936500	-3.00856200
H	0.56093300	2.04867100	-3.79787500
H	-0.02532400	0.58346200	-2.99993200
B	3.14481000	0.06011300	0.22611900
H	3.03430300	1.21813100	0.42146000
B	2.95354200	-3.26823600	-0.37157800
H	2.87316600	-4.43659400	-0.57999500
B	3.60445500	-0.60676200	-1.33664000
H	3.90781400	0.13188000	-2.21542700
B	2.37508700	-2.55232400	1.19018200
H	1.84948800	-3.14023200	2.07637300
B	4.55958200	-0.92760000	0.16574400
H	5.62015400	-0.41815100	0.34041100
B	2.61793900	-2.05714500	-1.67536600
H	2.25281800	-2.29549100	-2.77934800
B	1.51789700	-2.31447800	-0.33093000
H	0.40878100	-2.67285100	-0.51215800
B	4.26250900	-2.23238400	-1.00631600
H	5.14208400	-2.68764300	-1.66585100
B	4.11407300	-2.54391600	0.77094600
H	4.89122600	-3.21667600	1.37024900
B	3.37223100	-1.10522700	1.52036300
H	3.51323000	-0.71581500	2.63277000

N	-1.01461000	-0.84134900	0.09521900
N	-1.25977500	-1.67363300	0.98281700
N	-1.38041600	-2.37059500	1.87199100
C	-1.88980900	-0.84529500	-1.11530800
H	-1.49441300	-1.60278500	-1.80256600
H	-1.71990700	0.13855400	-1.55348500
C	-3.34641000	-1.07173200	-0.80843800
C	-4.19431300	0.01373300	-0.55492300
C	-3.85586200	-2.37389200	-0.72858000
C	-5.53225900	-0.19943800	-0.22523100
H	-3.80234800	1.02552800	-0.61483600
C	-5.19399200	-2.58823900	-0.39619600
H	-3.20419300	-3.22079400	-0.93212200
C	-6.03292700	-1.50112700	-0.14352600
H	-6.18338300	0.64878500	-0.03382900
H	-5.58078500	-3.60162500	-0.33854300
H	-7.07537900	-1.66751200	0.11307900

Int2

SCF Done: -1664.625835

Si	-0.38978800	1.90407800	1.65939600
Si	0.20289900	2.32369700	-1.34822100
N	0.10089800	1.26370400	0.07490300
C	1.83975500	-0.91034600	0.74792200
C	1.91062700	-0.63999200	-0.80727800
C	0.12640800	0.73526300	3.04800400
H	-0.37132500	-0.23921300	3.01580100
H	-0.14617900	1.20155300	4.00332200
H	1.20551200	0.55781000	3.05742100
C	0.45810700	3.56082300	1.98798300
H	1.54716700	3.43898500	1.97420900
H	0.17573600	3.93088800	2.98141100
H	0.19892400	4.33817000	1.26185100
C	-1.27887400	3.50536100	-1.37040600
H	-2.22982800	2.95960000	-1.39835700
H	-1.22620900	4.11874000	-2.27875100
H	-1.31221000	4.19041000	-0.51691300
C	-2.26650800	2.11871800	1.77590300
H	-2.66345800	2.79426300	1.01127900
H	-2.53291100	2.53339800	2.75645400
H	-2.78268400	1.15713500	1.67469600
B	0.59353000	-0.11115800	0.00719500
C	1.78740500	3.34832500	-1.39023600
H	1.97365100	3.86870600	-0.44593700
H	1.71503700	4.10384700	-2.18325200
H	2.65899500	2.72185000	-1.60313300
C	0.12521600	1.35211700	-2.97308600
H	0.76125300	0.46241700	-2.97768900
H	0.48313200	2.01144500	-3.77386400
H	-0.89573000	1.05650000	-3.24447900
B	2.93895500	0.33764800	0.24941700
H	2.63000700	1.45202100	0.46592900
B	3.37058400	-2.96185700	-0.37782500
H	3.50578400	-4.12398900	-0.59479500
B	3.50887700	-0.21676400	-1.31886200
H	3.67206200	0.56480700	-2.19754200
B	2.66311100	-2.37533500	1.18428400
H	2.25160300	-3.05715600	2.06482700
B	4.51009000	-0.36920100	0.18124200
H	5.46098900	0.32298000	0.36043700
B	2.81300700	-1.82355900	-1.67160400
H	2.50002300	-2.11639500	-2.77935200
B	1.78647300	-2.29216400	-0.33428300
H	0.76473300	-2.85960400	-0.51442900
B	4.46297100	-1.69648700	-1.00105300
H	5.41238400	-1.97237800	-1.66346600
B	4.37166500	-2.04522600	0.77190800
H	5.25880500	-2.56347200	1.37259600
B	3.37104300	-0.77407400	1.52850700
H	3.43699700	-0.37620700	2.64524200
N	-0.76035500	-1.09747100	-0.03137900
N	-0.97357600	-1.87632500	0.92327200
N	-1.12827700	-2.53273700	1.83141300
C	-1.65800700	-1.25129800	-1.22345600
H	-1.31985300	-2.13833500	-1.76968100
H	-1.42909200	-0.36918800	-1.81652700
C	-3.11212500	-1.32206600	-0.84855000
C	-3.82700700	-0.14286600	-0.60098500
C	-3.74747900	-2.55950900	-0.69496100
C	-5.16080100	-0.20206500	-0.20208300
H	-3.33103200	0.81689500	-0.71466500
C	-5.08446700	-2.61902600	-0.29728600
H	-3.19807000	-3.47723500	-0.89326500
C	-5.79039300	-1.44086400	-0.04884200
H	-5.70860000	0.71629400	-0.01154600
H	-5.57214200	-3.58282300	-0.18346300
H	-6.83053700	-1.48632500	0.26123400

TS4

SCF Done: -1664.615522

Si	-0.30174500	2.18870100	1.49798800
Si	1.15694200	2.50045700	-1.24514800
N	0.40899500	1.47085400	0.01749700
C	1.45019600	-1.51814900	0.96136800
C	1.69658300	-0.86374000	-0.48139900
C	-0.41020200	0.90460400	2.87589500
H	-1.30749200	0.28032200	2.80470800
H	-0.46471700	1.43101400	3.83676700
H	0.46108700	0.24277000	2.89923900
C	0.82776800	3.57530900	2.10021200
H	1.84185200	3.19748800	2.27172000
H	0.44769100	3.95906200	3.05506000
H	0.89506700	4.42162400	1.40982100
C	0.03582500	3.99675500	-1.54462600
H	-0.96224100	3.69051600	-1.87977900
H	0.47723100	4.60447400	-2.34481600
H	-0.08669800	4.64788700	-0.67330400
C	-2.03987100	2.85619600	1.17654400
H	-2.08955500	3.49922800	0.29236800
H	-2.38337900	3.44338000	2.03751400
H	-2.74897500	2.03188700	1.04351400
B	0.47118100	0.05764900	-0.10591600
C	2.87452800	3.10906100	-0.77315800
H	2.83011400	3.85775600	0.02450700
H	3.35806200	3.57335300	-1.64180100
H	3.51157500	2.28914100	-0.43299400
C	1.17600100	1.54004700	-2.86945300
H	1.81600900	0.65574700	-2.85136400
H	1.54206400	2.19689000	-3.66838700
H	0.16347600	1.22294200	-3.15093000
B	2.77340500	-0.42682500	0.81350200
H	2.66565200	0.64995100	1.29419600
B	2.63254100	-3.44836000	-0.69721600
H	2.54716400	-4.51001200	-1.22920400
B	3.37345600	-0.66015700	-0.84263800
H	3.73748600	0.27628200	-1.47219200
B	1.97494300	-3.17045000	0.95483200
H	1.38562200	-3.97435200	1.60352000
B	4.21415000	-1.38176400	0.56696500
H	5.26935800	-0.96695200	0.92946700
B	2.40488000	-1.93360200	-1.63089500
H	2.10330900	-1.84067700	-2.77700100
B	1.20206900	-2.48532700	-0.45787000
H	0.09890100	-2.75827400	-0.80582000
B	3.99878300	-2.32061200	-0.93768800
H	4.92044900	-2.58708100	-1.64320100
B	3.73506900	-3.10024200	0.66305200
H	4.47081600	-3.92646000	1.10370500
B	2.94007300	-1.90287800	1.72928400
H	3.01833800	-1.84141200	2.91369900
N	-0.96394500	-0.59932300	-0.01964600
N	-1.18419400	-1.50571100	0.84594100
N	-1.64273800	-2.24966500	1.56035800
C	-1.89151200	-0.58179200	-1.21681600
H	-1.48640300	-1.31035000	-1.92703600
H	-1.74302600	0.42423400	-1.61411800
C	-3.32813200	-0.85766400	-0.88111000
C	-4.21094500	0.19452000	-0.60397500
C	-3.79253000	-2.17919800	-0.81918600
C	-5.53508700	-0.07089800	-0.25846700
H	-3.86143700	1.22084900	-0.66935300
C	-5.11680500	-2.44457400	-0.46997000
H	-3.11714500	-3.00002600	-1.04954300
C	-5.98781000	-1.39085300	-0.18666900
H	-6.21354300	0.75103900	-0.04913600
H	-5.46742200	-3.47137400	-0.42391100
H	-7.01966000	-1.59706600	0.08306700

Int3

SCF Done: -1664.692454

Si	-1.41670500	1.80421500	1.30680100
Si	0.40240200	2.99493400	-0.90516700
N	-0.19835600	1.59944800	0.02169800
C	1.78843100	-1.79543700	-0.39982400
C	1.77243300	-0.28113600	0.13373800
C	-1.33517000	0.34123200	2.48927900
H	-1.61283200	-0.59871000	2.00392500
H	-2.04367600	0.50568600	3.31069500
H	-0.33783500	0.22716900	2.92742100
C	-1.03766900	3.36750200	2.29899400
H	-0.02537600	3.33994000	2.71700500
H	-1.74335700	3.43368900	3.13671400
H	-1.13849600	4.28927700	1.71650600
C	-1.05752000	4.07199900	-1.43706600
H	-1.77306800	3.50047500	-2.03950200
H	-0.68981000	4.90187200	-2.05366200

H	-1.60281700	4.50898800	-0.59413000
C	-3.16347000	1.93736900	0.60636600
H	-3.25401400	2.74858400	-0.12341300
H	-3.87561400	2.13802100	1.41714800
H	-3.47322500	1.00517200	0.12394100
B	0.31787000	0.29113300	-0.26918600
C	1.62192900	4.04403400	0.08057400
H	1.13402800	4.58991000	0.89388700
H	2.10058800	4.78171900	-0.57629900
H	2.41236800	3.42603700	0.51884000
C	1.25013300	2.37595900	-2.47567500
H	2.25933100	1.99921000	-2.28710400
H	1.34033100	3.20971600	-3.18291100
H	0.67911900	1.58313400	-2.97295700
B	1.58998200	-1.57388600	1.28982400
H	0.51026900	-1.75206400	1.72872500
B	4.37384800	-0.95294000	-0.56368900
H	5.33482100	-0.72615300	-1.22409300
B	2.62403200	-0.17281900	1.61118300
H	2.25902700	0.62544900	2.40837000
B	3.31044000	-2.35025500	-0.91493800
H	3.35312000	-3.09542300	-1.83335300
B	3.19133300	-1.84462200	1.93914200
H	3.31363600	-2.25135700	3.04854800
B	3.34808000	0.36957200	0.07924200
H	3.47943000	1.52483400	-0.14112700
B	2.77907100	-0.67740700	-1.23247100
H	2.40094700	-0.32748500	-2.29428200
B	4.29270500	-0.64298800	1.19938300
H	5.21865500	-0.19622700	1.79485600
B	4.26509800	-2.32575300	0.58537900
H	5.16736400	-3.08279000	0.74156200
B	2.58087400	-2.89799900	0.62438300
H	2.14932600	-3.99781900	0.70274000
N	-0.34518300	-0.76297700	-0.93035100
N	0.51494400	-2.06683500	-1.12042400
N	0.19613600	-3.02470100	-1.72677500
C	-1.61298800	-0.84731000	-1.61071200
H	-1.44894700	-1.41976200	-2.53583700
H	-1.89423900	0.17187800	-1.89597700
C	-2.73623600	-1.50376300	-0.82471200
C	-4.06216600	-1.23935300	-1.18936100
C	-2.49077100	-2.39387200	0.22489300
C	-5.12226600	-1.84307600	-0.51292500
H	-4.26624200	-0.54787100	-2.00456100
C	-3.55000800	-2.99673700	0.90641100
H	-1.47076100	-2.61755500	0.51766300
C	-4.86853200	-2.72335100	0.54109800
H	-6.14510100	-1.62063900	-0.80483400
H	-3.34081300	-3.68261500	1.72285600
H	-5.69213000	-3.19145300	1.07299700

TS5

SCF Done: -2894.048707

Si	4.69570600	1.18352600	-0.71510500
Si	3.65550100	-1.44645100	-2.09771400
N	3.46234700	-0.09301400	-0.93709500
C	1.04947500	-0.86031000	2.04523800
C	2.57683100	-0.76514500	1.57218400
C	4.06443800	2.43550500	0.54568700
H	3.08253000	2.83080800	0.26488900
H	4.76225100	3.28206500	0.56217200
H	4.00340800	2.04900400	1.56483400
C	6.31865000	0.41196100	-0.14134700
H	6.18292800	-0.13614900	0.79809800
H	7.08241200	1.18164200	0.02463100
H	6.71430600	-0.29178500	-0.88366200
C	4.65960300	-0.90871400	-3.59924300
H	4.21825000	-0.05044900	-4.11563000
H	4.67808700	-1.74856000	-4.30525500
H	5.69960900	-0.66690200	-3.35632200
C	4.97002800	2.15148200	-2.31297900
H	5.58319300	1.62814600	-3.05025000
H	5.48202300	3.08901200	-2.06120700
H	4.01652500	2.41623800	-2.78116400
B	2.45382700	-0.23776100	0.05680100
C	4.55233300	-2.88206800	-1.26652200
H	5.47541300	-2.55581700	-0.77495900
H	4.81664300	-3.64237000	-2.01230200
H	3.92688900	-3.36632800	-0.50941600
C	1.97831100	-2.06440700	-2.69530800
H	1.27961600	-2.28322600	-1.88303500
H	2.12999600	-2.99944500	-3.24877400
H	1.48542400	-1.36431400	-3.37899100
B	1.99154300	0.37876700	2.75273200
H	1.79011500	1.47784400	2.36858300
B	2.29030900	-2.98791000	3.21552300
H	2.39112600	-4.16203300	3.35881500
B	3.57544200	-0.40799400	2.91407600
H	4.55450500	0.23544000	2.74607600
B	0.68894800	-2.20964700	3.01425800
H	-0.37184400	-2.71803000	2.88648200
B	2.44695800	-0.29108600	4.30402800
H	2.66053700	0.43841100	5.21640800
B	3.48038400	-2.05409200	2.25109300
H	4.39178100	-2.48413800	1.63255400
B	1.83822500	-2.34458800	1.65348500
H	1.54706900	-2.84296300	0.62204900
B	3.36977300	-1.79752800	4.00738400
H	4.25037800	-2.13805900	4.72783400
B	1.64629200	-1.89067700	4.48041100
H	1.28943800	-2.29626100	5.53793700
B	0.78630700	-0.55139400	3.69010600
H	-0.21049600	-0.00138600	4.00916900
N	1.05420100	-0.01681700	-0.16907800
N	0.18361200	-0.38204700	0.93488800
N	-1.01528000	-0.41564000	0.97054400
Si	-3.78228800	2.20275500	-0.70734200
Si	-3.73805600	0.90692500	2.13858900
N	-3.34082000	0.86157400	0.39217300
C	-2.80408900	-1.09278800	-1.53717400
C	-3.08107000	-1.91919900	-0.19309000
C	-2.78870900	2.17746800	-2.30973000
H	-1.72978900	2.40060200	-2.15357000
H	-3.19218600	2.96711700	-2.95695200
H	-2.87564000	1.23342700	-2.85362600
C	-5.60857600	2.11518100	-1.17851900
H	-5.80601500	1.26473500	-1.83916100
H	-5.89221600	3.02808200	-1.71787100
H	-6.26577900	2.02353300	-0.30851000
C	-2.47176500	1.98834400	3.03400400
H	-1.46048300	1.59041500	2.89834600
H	-2.67302200	2.00903800	4.11235100
H	-2.48335000	3.02123300	2.67124200
C	-3.44688900	3.86317200	0.13230800
H	-4.02374200	4.01815200	1.04958300
H	-3.71920500	4.66630700	-0.56415900
H	-2.38460800	3.98099800	0.36852200
B	-2.77876100	-0.33223300	-0.11778800
C	-5.46914900	1.61970400	2.39780800
H	-5.59041000	2.63958800	2.01965800
H	-5.69100900	1.63823000	3.47234800
H	-6.22493400	0.98890400	1.91604400
C	-3.72340400	-0.83050500	2.86181800
H	-4.44660600	-1.48197700	2.35955900
H	-3.99740500	-0.77926600	3.92318000
H	-2.73842200	-1.29785400	2.78464600
B	-4.46926300	-1.53926500	-1.17433700
H	-5.19661700	-0.70022200	-0.77946300
B	-2.01675800	-3.70246500	-2.04919100
H	-1.14158400	-4.45185500	-2.34740100
B	-4.31856700	-3.10083800	-0.38324800
H	-5.03832700	-3.35961400	0.52426100
B	-2.12325600	-2.05120200	-2.78378000
H	-1.34444800	-1.56979000	-3.54060800
B	-4.80478200	-2.94688600	-2.12041500
H	-5.92343600	-3.15342600	-2.46790800
B	-2.59564800	-3.56676100	-0.33535500
H	-2.14461900	-4.13991900	0.60078100
B	-1.65413100	-2.30511200	-1.11050400
H	-0.60195800	-1.96654000	-0.71118800
B	-3.66515700	-4.22075600	-1.61042300
H	-3.97492700	-5.36963100	-1.62172400
B	-3.37401300	-3.27718600	-3.12882300
H	-3.47394700	-3.75986400	-4.21197500
B	-3.84814200	-1.58317300	-2.82334000
H	-4.23647700	-0.78742700	-3.61434600
C	0.43164400	0.73442700	-1.25510400
H	-0.62671000	0.47047900	-1.28758200
H	0.87654300	0.38867500	-2.18702700
C	0.63228600	2.22483800	-1.07974600
C	1.25575700	2.97870000	-2.07741700
C	0.21671700	2.86332800	0.09705900
C	1.47685500	4.34701100	-1.90119300
H	1.57501700	2.49089700	-2.99502100
C	0.44228900	4.22697700	0.27812100
H	-0.29299300	2.29726600	0.87016700
C	1.07640600	4.97242000	-0.72022800
H	1.96869900	4.91877900	-2.68306900
H	0.11851600	4.70773300	1.19705700
H	1.25398500	6.03446800	-0.57739600

Int4

SCF Done: -2894.060093

Si	4.67354100	0.99919600	-0.70382900
Si	3.63694500	-1.76959100	-1.78693200

N	3.39141200	-0.25345000	-0.84388400
C	0.84002900	-0.54409900	2.08598200
C	2.39066000	-0.49478400	1.69567500
C	4.01092300	2.46230400	0.28167100
H	3.09917700	2.86881800	-0.16553200
H	4.76868200	3.25554800	0.25206200
H	3.81547900	2.24665700	1.33269200
C	6.19772400	0.26540500	0.12963800
H	5.95313400	-0.13048100	1.12169300
H	6.97637600	1.02785500	0.25474300
H	6.62858700	-0.55303600	-0.45911700
C	4.81900500	-1.48790400	-3.22595500
H	4.47645600	-0.70792200	-3.91272200
H	4.87391800	-2.42706600	-3.79091000
H	5.83664600	-1.24304300	-2.90509400
C	5.12373000	1.68828400	-2.40352400
H	5.82558900	1.06630800	-2.96346100
H	5.59373100	2.66887700	-2.25659400
H	4.23220800	1.84340900	-3.01930300
B	2.34623700	-0.25885900	0.10975700
C	4.38227200	-3.08467900	-0.66007600
H	5.28493600	-2.72262400	-0.15610800
H	4.65581500	-3.96933100	-1.24866400
H	3.67799600	-3.40906200	0.11281900
C	2.00990900	-2.41923700	-2.47433100
H	1.21003300	-2.48517300	-1.73209200
H	2.17842800	-3.43559600	-2.85175900
H	1.63868300	-1.82545500	-3.31685300
B	1.72125600	0.81548900	2.63581400
H	1.53170600	1.82981700	2.06099500
B	2.03789300	-2.42526100	3.66508800
H	2.14438100	-3.55766600	4.00394400
B	3.30140100	0.09910400	3.01835900
H	4.28065400	0.72680800	2.80646200
B	0.44013700	-1.72981300	3.24334200
H	-0.60209800	-2.27816300	3.13460600
B	2.08915200	0.41679300	4.29996400
H	2.23589100	1.29122400	5.08926000
B	3.26992400	-1.63356500	2.62924600
H	4.22065800	-2.13646800	2.13715200
B	1.67353800	-2.06262700	1.99666700
H	1.45401500	-2.72992900	1.04735800
B	3.04967700	-1.09699000	4.31067500
H	3.88933600	-1.29360800	5.12673100
B	1.30023200	-1.15112200	4.68848200
H	0.88234000	-1.38591700	5.77457200
B	0.47222400	0.02016800	3.64246600
H	-0.55013700	0.58476200	3.81062500
N	0.93639200	-0.14062900	-0.23037000
N	0.05471600	-0.28442700	0.86124100
N	-1.16319100	-0.33043100	0.91372300
Si	-3.61387400	2.07336100	-0.99716900
Si	-3.65131300	1.21438900	1.96313600
N	-3.14246900	0.92269900	0.27830200
C	-2.57482400	-1.27012100	-1.34475600
C	-2.99500600	-1.94445100	0.02429300
C	-2.60891500	1.90389600	-2.58705200
H	-1.59656600	2.30463900	-2.49780400
H	-3.12524900	2.50074100	-3.35021900
H	-2.55695300	0.87989500	-2.96411400
C	-5.42864900	1.90992600	-1.51164500
H	-5.56823000	1.07800100	-2.20917100
H	-5.73866300	2.83054700	-2.02352500
H	-6.10438600	1.75299100	-0.66664700
C	-2.50475900	2.49143500	2.77018600
H	-1.45129700	2.24970500	2.59174400
H	-2.65422600	2.52271400	3.85677500
H	-2.68534600	3.49885900	2.38048800
C	-3.35417200	3.84789300	-0.38502400
H	-4.01207700	4.12414100	0.44492200
H	-3.56958700	4.53445600	-1.21365600
H	-2.31988200	4.02281200	-0.07373300
B	-2.43054800	-0.34204400	0.00563400
C	-5.42218400	1.87559700	2.05144400
H	-5.58208400	2.81406400	1.51176300
H	-5.67372600	2.05612300	3.10456700
H	-6.13400700	1.13719200	1.66537400
C	-3.64503600	-0.39036000	2.95318600
H	-4.29706800	-1.13741700	2.48797600
H	-4.03181600	-0.18617200	3.95971200
H	-2.65121100	-0.83304800	3.05369400
B	-4.28231600	-1.53110500	-1.04400400
H	-4.95309500	-0.61023100	-0.75287400
B	-2.02273400	-3.98139300	-1.61969000
H	-1.21059000	-4.83081400	-1.81097700
B	-4.32719800	-3.02708200	-0.13515200
H	-5.10987400	-3.14206200	0.75022000
B	-1.93354600	-2.39290700	-2.48067900
H	-1.07805300	-2.04989500	-3.23147200
B	-4.71526000	-2.97221500	-1.89990400
H	-5.82961700	-3.10281500	-2.29606400
B	-2.66647100	-3.64897300	0.04009900
H	-2.31689900	-4.18279100	1.04156500
B	-1.57719700	-2.54982600	-0.77178100
H	-0.51546200	-2.28547300	-0.33065000
B	-3.73238900	-4.30325700	-1.23422900
H	-4.15510700	-5.41456500	-1.17314300
B	-3.27878100	-3.51890600	-2.80032400
H	-3.37774100	-4.07365300	-3.84919900
B	-3.59730600	-1.76824600	-2.64943000
H	-3.86914400	-1.01345700	-3.52469000
C	0.41617100	0.49197200	-1.45061300
H	-0.64916500	0.28204700	-1.52648200
H	0.89633100	-0.00531800	-2.29108200
C	0.70613400	1.97680200	-1.45796900
C	1.44560800	2.54703900	-2.49701200
C	0.23991400	2.79662000	-0.42109700
C	1.72695400	3.91539800	-2.49968300
H	1.80522300	1.91603900	-3.30554600
C	0.52624100	4.16061600	-0.41744700
H	-0.37061700	2.37534800	0.37205100
C	1.27326700	4.72342800	-1.45643400
H	2.30689100	4.34504100	-3.31151200
H	0.15830700	4.78441200	0.39215600
H	1.49655200	5.78638500	-1.45266300

TS6

SCF Done: -2894.058679

Si	4.63887300	1.09267500	-0.72460700
Si	3.68914500	-1.70410400	-1.81330500
N	3.40619700	-0.20726900	-0.85268300
C	0.85208700	-0.52982700	2.07832800
C	2.40269800	-0.49519100	1.68415300
C	3.94193400	2.52338700	0.28634500
H	2.97721600	2.86704500	-0.09946300
H	4.64248200	3.36365200	0.20034400
H	3.83332200	2.30436400	1.34940700
C	6.20415900	0.41617500	0.08032600
H	5.99633600	0.03718200	1.08718400
H	6.96713200	1.19958000	0.16717200
H	6.63824900	-0.40512400	-0.50190200
C	4.94203100	-1.40169200	-3.18727800
H	4.61470300	-0.64002600	-3.90130100
H	5.05123200	-2.34509900	-3.73720100
H	5.93488900	-1.12540200	-2.81811700
C	5.03404500	1.80127800	-2.43177000
H	5.78829000	1.23065800	-2.97869000
H	5.41887300	2.82011400	-2.29988900
H	4.13662200	1.86898500	-3.05461400
B	2.35840500	-0.24662500	0.10042300
C	4.37022700	-3.04537100	-0.67760900
H	5.25821300	-2.70154500	-0.13567500
H	4.65345000	-3.92722700	-1.26565300
H	3.63261500	-3.36835800	0.06422000
C	2.09518400	-2.32553200	-2.59803500
H	1.26914600	-2.43125000	-1.88985900
H	2.28305500	-3.32121000	-3.01902200
H	1.75445900	-1.68992700	-3.42323200
B	1.74679500	0.82847600	2.61664400
H	1.55919300	1.83847800	2.03384200
B	2.03944300	-2.40522300	3.67113200
H	2.13838600	-3.53526200	4.02010300
B	3.32172500	0.10298200	2.99907400
H	4.30535100	0.72067500	2.77697700
B	0.44598400	-1.70118500	3.24715400
H	-0.60134000	-2.24104200	3.14651600
B	2.11672300	0.44089700	4.28191900
H	2.27223300	1.32105900	5.06331600
B	3.27512000	-1.63312600	2.62500500
H	4.22067800	-2.14913400	2.13645900
B	1.67393900	-2.05532700	2.00014600
H	1.44523100	-2.72863200	1.05767200
B	3.06437000	-1.08021000	4.30267300
H	3.90444800	-1.27712500	5.11832600
B	1.31480200	-1.11655100	4.68551400
H	0.89822200	-1.33868000	5.77485100
B	0.49506700	0.05270900	3.63188100
H	-0.52076700	0.63046300	3.79809600
N	0.95095200	-0.13151800	-0.23781000
N	0.07246600	-0.26601100	0.86048600
N	-1.15507400	-0.25516200	0.92713500
Si	-3.75853300	1.93330900	-1.09112300
Si	-3.64391700	1.25576300	1.93788000
N	-3.17165300	0.90673500	0.24766500
C	-2.56439200	-1.32343300	-1.19165100
C	-2.76922800	-2.14595900	0.15451500

C	-2.69803200	1.76281900	-2.64584200
H	-1.73231200	2.26381700	-2.53369700
H	-3.23187700	2.26659400	-3.46190900
H	-2.53016200	0.73212300	-2.96550300
C	-5.54726900	1.55699100	-1.55833300
H	-5.63763800	0.56750000	-2.01597900
H	-5.89645800	2.29909000	-2.28793000
H	-6.22007600	1.59179400	-0.69653800
C	-2.52052400	2.60274900	2.65123700
H	-1.46565800	2.31238500	2.59507700
H	-2.75225200	2.79047000	3.70705100
H	-2.63805300	3.54769800	2.10949700
C	-3.62268800	3.75490800	-0.59171100
H	-4.22015300	4.02777200	0.28295600
H	-3.96935800	4.37231900	-1.43019900
H	-2.58047900	4.02739300	-0.39400300
B	-2.35112300	-0.25993000	-0.01320700
C	-5.43814600	1.85050700	2.01657800
H	-5.64653600	2.76038800	1.44625700
H	-5.68911900	2.05592900	3.06513600
H	-6.11847600	1.06688200	1.66430500
C	-3.57419900	-0.30737600	2.98595500
H	-4.16401000	-1.10654500	2.52472500
H	-4.00564800	-0.09379100	3.97218400
H	-2.56057800	-0.68702300	3.12898900
B	-4.19245100	-1.76897100	-0.72465800
H	-4.90809400	-0.93724400	-0.29217800
B	-1.80554200	-3.91967000	-1.77523900
H	-0.94212800	-4.65975400	-2.12899300
B	-4.00846500	-3.34131100	0.01400600
H	-4.67955900	-3.61211800	0.95570200
B	-1.95313500	-2.27110400	-2.48444300
H	-1.21733300	-1.77231800	-3.27486800
B	-4.59335200	-3.16468600	-1.68280500
H	-5.72886600	-3.36403900	-1.97790700
B	-2.29709600	-3.80502100	-0.03953300
H	-1.79051400	-4.39111600	0.86105900
B	-1.40893200	-2.52816300	-0.82638400
H	-0.33686600	-2.20175500	-0.46585500
B	-3.42878400	-4.45156200	-1.26333500
H	-3.74000100	-5.60085100	-1.26971600
B	-3.22038800	-3.48788100	-2.77661200
H	-3.38571000	-3.95429000	-3.85975500
B	-3.67649700	-1.80253700	-2.42029900
H	-4.10815900	-1.00371900	-3.18410500
C	0.42801500	0.50754600	-1.45252800
H	-0.61352100	0.22035900	-1.58589100
H	0.97890300	0.07289200	-2.28406600
C	0.61048100	2.00964600	-1.41427500
C	1.34488200	2.64860200	-2.41694600
C	0.06435000	2.77617900	-0.37508200
C	1.54880500	4.02983100	-2.37869200
H	1.76378500	2.06032700	-3.22905000
C	0.27636200	4.15325200	-0.32912000
H	-0.55069200	2.30711700	0.38540800
C	1.02264400	4.78363400	-1.32912900
H	2.12629000	4.51149000	-3.16257600
H	-0.14980500	4.73334800	0.48453700
H	1.18741600	5.85657000	-1.29130000

3

SCF Done: -2894.132709

Si	4.58681800	1.13174800	-0.35947000
Si	3.61486900	-1.41274200	-1.90829500
N	3.34436400	-0.09280900	-0.73767300
C	0.48660900	-0.59719600	1.91965600
C	2.09483000	-0.66365700	1.64624600
C	3.87448400	2.49513700	0.73422700
H	2.92015200	2.87859000	0.36407300
H	4.58656700	3.33035200	0.72337300
H	3.74547100	2.19535400	1.77580600
C	6.05269400	0.33939900	0.52881100
H	5.72523700	-0.15737900	1.44858400
H	6.79470000	1.09944900	0.80474300
H	6.55918400	-0.40930400	-0.09015200
C	5.24238100	-1.21579600	-2.84617800
H	5.21727000	-0.38442000	-3.55679600
H	5.38848000	-2.13712000	-3.42452600
H	6.11972800	-1.09359200	-2.20421600
C	5.17717900	1.97224100	-1.95196000
H	6.00841300	1.44625600	-2.42928500
H	5.51989300	2.98621600	-1.71223900
H	4.36721300	2.06534300	-2.68338500
B	2.18854400	-0.22059500	0.10680300
C	3.67640900	-3.06222400	-0.99750600
H	4.47843500	-3.06674400	-0.25060800
H	3.86829000	-3.87812400	-1.70542700
H	2.74173700	-3.29296400	-0.47821800
C	2.26083800	-1.47804200	-3.22376800
H	1.26130800	-1.61418100	-2.80410500
H	2.45066400	-2.33035400	-3.88844700
H	2.25760700	-0.57419500	-3.84514100
B	1.46506800	0.62616200	2.63332600
H	1.40260400	1.69607700	2.13916300
B	1.46608900	-2.66620000	3.46121600
H	1.45136800	-3.82379400	3.72738300
B	2.95623400	-0.22722500	3.05606700
H	3.98770900	0.34000400	2.94502100
B	-0.03525800	-1.80624200	2.98645500
H	-1.10432100	-2.28273000	2.82079100
B	1.70707300	0.10535600	4.28892000
H	1.87512600	0.91852700	5.13910500
B	2.82380800	-1.92590400	2.55963600
H	3.75926300	-2.48326300	2.09318200
B	1.24846100	-2.15558900	1.80571100
H	1.05665100	-2.76356400	0.81831700
B	2.53879400	-1.47682600	4.25543700
H	3.30642500	-1.79227000	5.10596100
B	0.76883600	-1.40085400	4.52399200
H	0.26034500	-1.67320300	5.56324200
B	0.11531000	-0.10092400	3.51413300
H	-0.85229400	0.55602800	3.68513800
N	0.84668500	0.03689000	-0.30116900
N	-0.17455500	-0.11908300	0.72243700
N	-1.30585000	-0.79851400	0.31522000
Si	-3.92366500	1.83150800	-1.37616900
Si	-3.62308600	1.51304800	1.71695200
N	-3.22515300	0.98094400	0.04376200
C	-2.90554600	-1.64515900	-0.94640900
C	-1.48680000	-2.14612000	-0.32484300
C	-2.91479100	1.47778300	-2.92946100
H	-1.91064200	1.91127400	-2.87084100
H	-3.42364900	1.96024200	-3.77395500
H	-2.82655300	0.41642100	-3.17393200
C	-5.69479400	1.26272500	-1.67515000
H	-5.72733000	0.17309400	-1.79764900
H	-6.09210300	1.71148100	-2.59394200
H	-6.36692900	1.52780100	-0.85365900
C	-2.48542700	2.88873800	2.32067200
H	-1.46847600	2.52878500	2.50441200
H	-2.86922300	3.28521800	3.26903600
H	-2.43567100	3.71761200	1.60677300
C	-3.78187400	3.69052500	-1.08636100
H	-4.34049900	4.06436500	-0.22405400
H	-4.15074800	4.22011400	-1.97355400
H	-2.72810700	3.96311300	-0.95333400
B	-2.56980100	-0.24157700	-0.16732900
C	-5.41015400	2.12912500	1.77288400
H	-5.62625300	2.99851600	1.14591700
H	-5.63300800	2.41129500	2.81000100
H	-6.10538700	1.32857700	1.49695500
C	-3.57957600	0.04252600	2.88853000
H	-4.42197600	-0.62904800	2.68858800
H	-3.68325600	0.40905600	3.91733900
H	-2.66154300	-0.54284000	2.84181400
B	-2.95836400	-2.75067900	0.40069500
H	-3.24435900	-2.30363600	1.45195000
B	-1.84060100	-3.49677500	-2.71027400
H	-1.43769900	-3.74810700	-3.79979000
B	-1.54579400	-3.78608300	0.17164100
H	-0.93188900	-4.14508900	1.11497100
B	-3.23762000	-2.39698700	-2.44776400
H	-3.81137600	-1.77971000	-3.28238000
B	-3.20891700	-4.32345500	-0.28986800
H	-3.77488100	-5.15919700	0.33700300
B	-0.69695100	-3.27233800	-1.32484600
H	0.48304500	-3.29927300	-1.38546000
B	-1.58661400	-1.92273000	-2.04091200
H	-1.04519900	-0.98129600	-2.49141900
B	-1.81340400	-4.66585900	-1.35322400
H	-1.39222200	-5.76832600	-1.49473500
B	-3.37804200	-4.12954200	-2.05508800
H	-4.07272300	-4.85197300	-2.69354800
B	-4.06977400	-2.89503900	-0.96074600
H	-5.20944700	-2.61342400	-0.78868900
C	0.45042900	0.91173600	-1.40787600
H	-0.60696800	0.73897200	-1.60142700
H	0.99000700	0.57495500	-2.29107600
C	0.69165000	2.38558400	-1.17860400
C	1.52349900	3.11138700	-2.03618400
C	0.06991400	3.04544600	-0.11186000
C	1.74777500	4.47346900	-1.82159200
H	2.00671600	2.60589100	-2.86840500
C	0.29675000	4.40290200	0.10964900
H	-0.58221700	2.48083900	0.54075800
C	1.13926500	5.12054800	-0.74505500

H	2.40408100	5.02424400	-2.48976300
H	-0.18313400	4.89823100	0.94953100
H	1.31901200	6.17801500	-0.57218000

TMSN3
SCF Done: -573.611528

Si	-0.66336300	0.00017200	0.02017100
C	-0.72155100	-1.54686600	1.09237300
H	-0.67358700	-2.45485700	0.48088100
H	-1.64859700	-1.58277700	1.67801400
H	0.11710200	-1.57247600	1.79856100
C	-0.71809700	1.54917700	1.08970800
H	0.12003100	1.57357800	1.79656300
H	-1.64552800	1.58870500	1.67449400
H	-0.66703200	2.45600700	0.47674400
C	-1.99793800	0.00013400	-1.29635100
H	-1.91755500	0.88609100	-1.93584300
H	-2.99607100	-0.00057000	-0.84230300
H	-1.91654200	-0.88546600	-1.93622600
N	0.86428100	-0.00229600	-0.90269900
N	1.97173500	-0.00111300	-0.37825500
N	3.04118100	-0.00020700	0.02557300

TS1-TMS
SCF Done: -1802.938318

B	2.50360200	-0.44213100	-0.44294300
H	2.64431600	0.63906300	-0.88283600
B	1.69316500	-3.46675000	0.79084400
H	1.38257000	-4.52917200	1.22756300
B	2.41489300	-1.83415200	-1.49772400
H	2.55934200	-1.69585000	-2.66799600
B	1.69439100	-1.98845200	1.83554000
H	1.36395600	-1.93483200	2.97520700
B	3.70183900	-1.66440900	-0.23513500
H	4.83157200	-1.43558800	-0.53094800
B	1.17262900	-2.94180100	-0.85752200
H	0.48946300	-3.55851700	-1.60663300
B	0.48774400	-2.25060600	0.60373600
H	-0.65240500	-2.29956400	0.87943000
B	2.89679400	-3.23501900	-0.50082900
H	3.47866100	-4.15592500	-0.98033700
B	3.22198900	-2.63932700	1.17549400
H	4.03263300	-3.13310300	1.89369000
B	2.93421900	-0.88111500	1.20169000
H	3.45298300	-0.09957900	1.92420400
C	0.99982700	-1.22211800	-0.72745100
C	1.26345100	-0.70948200	0.74394000
B	0.08689900	0.03145700	-0.15387000
N	-1.41553400	-0.06290000	-0.03166400
Si	-2.12824400	0.38563600	1.58115900
Si	-2.39071900	-1.03621400	-1.23271000
C	-3.03433900	-1.09155300	2.32498000
H	-3.39755500	-0.81264600	3.32218700
H	-3.90224100	-1.40618200	1.73628300
H	-2.36969900	-1.95353000	2.44101300
C	-3.32163100	1.83785700	1.41700900
H	-3.79530800	2.01677200	2.39079400
H	-2.79835700	2.75603900	1.12876800
H	-4.11349900	1.66302100	0.68427400
C	-0.75447600	0.91641300	2.75418000
H	-0.07620800	0.09615700	3.00259100
H	-0.15787300	1.73575600	2.34010700
H	-1.21175000	1.27658300	3.68430100
C	-4.14431200	-0.33595300	-1.29981700
H	-4.70207800	-0.89990200	-2.05824200
H	-4.68360900	-0.45663500	-0.35395400
H	-4.17140900	0.72035000	-1.57957500
C	-1.58475200	-0.90436700	-2.92909400
H	-1.57599400	0.12224200	-3.30856700
H	-0.55730200	-1.27887900	-2.92286900
H	-2.15817300	-1.51631000	-3.63658800
C	-2.58495100	-2.86136700	-0.79813400
H	-3.35492900	-3.26732400	-1.46820200
H	-1.67119200	-3.43772200	-0.95338200
H	-2.92222000	-3.02723700	0.22878200
N	0.40317800	1.58292800	-0.57817600
N	-0.66544500	2.08667600	-1.12178100
N	-1.77856600	1.75854600	-1.21366900
Si	1.64038800	2.93516200	-0.30314400
C	2.59825700	3.21490300	-1.89367100
H	1.91236800	3.38397700	-2.73194500
H	3.22644600	4.10909400	-1.79384900
H	3.24640900	2.37194100	-2.14890600
C	0.57164400	4.44423700	0.06951200
H	-0.07804200	4.26845500	0.93559300
H	1.21816000	5.29696000	0.31112800
H	-0.06130200	4.73733500	-0.77465600
C	2.70179700	2.57372300	1.19495200
H	3.60375900	2.01138600	0.94770300
H	3.00174300	3.53182000	1.63727400
H	2.15747700	2.00838700	1.95651400

Int1-TMS
SCF Done: -1802.94608

B	2.49652500	-0.51512300	-0.30319300
H	2.72155100	0.58491600	-0.63470400
B	1.43034300	-3.57489800	0.58476100
H	1.02581400	-4.64905400	0.89839000
B	2.41130200	-1.79632000	-1.48630300
H	2.64920900	-1.55666400	-2.62444900
B	1.44703200	-2.20226800	1.76174200
H	1.04395000	-2.23656500	2.87829300
B	3.60986000	-1.82072400	-0.13115500
H	4.76860800	-1.63735700	-0.32902600
B	1.07027200	-2.87709500	-1.04155500
H	0.39836400	-3.37896700	-1.88264900
B	0.32522100	-2.26055300	0.41898400
H	-0.83467700	-2.25713600	0.58138900
B	2.74071600	-3.30817400	-0.59416200
H	3.30202200	-4.21804300	-1.11716200
B	2.97617800	-2.88504100	1.14843400
H	3.70161900	-3.49634800	1.86711600
B	2.78970000	-1.11739000	1.31976500
H	3.29411700	-0.41982100	2.13545100
C	0.97681200	-1.16732700	-0.75590100
C	1.16807000	-0.80621700	0.78170000
B	0.15673900	0.12897500	-0.12286600
N	-1.44171200	0.15600900	-0.06877200
Si	-2.13844000	0.27909300	1.68611000
Si	-2.38184800	-0.73923200	-1.46537700
C	-2.15569300	-1.35394900	2.60951200
H	-2.70048600	-1.16674400	3.54523800
H	-2.68552400	-2.14876600	2.07663700
H	-1.15911000	-1.71661300	2.86645900
C	-3.90748400	0.90840000	1.59007300
H	-4.18624500	1.27546800	2.58626300
H	-4.02535300	1.72789900	0.87813900
H	-4.61157300	0.11130100	1.33050900
C	-1.02442000	1.50894400	2.56576300
H	-0.00100500	1.13095000	2.64550300
H	-1.00106300	2.47893200	2.06048300
H	-1.40648200	1.66703700	3.58168600
C	-4.06254900	0.07241800	-1.70989600
H	-4.49449500	-0.36790300	-2.61867300
H	-4.75913600	-0.12979300	-0.89174200
H	-3.99504800	1.15261300	-1.85003100
C	-1.32387300	-0.50000700	-2.99660900
H	-0.93540200	0.52173600	-3.05994000
H	-0.47678400	-1.18886200	-3.02354700
H	-1.94257100	-0.67719400	-3.88492000
C	-2.73033600	-2.54981500	-1.09946600
H	-3.47461400	-2.87236600	-1.84014700
H	-1.85812200	-3.19781400	-1.19115300
H	-3.17393100	-2.70220900	-0.11018000
N	0.42580500	1.61585500	-0.40909800
N	-0.71950600	2.24684000	-0.68161200
N	-1.78060300	1.61394000	-0.55206500
Si	1.77228700	2.85643100	-0.27964700
C	2.72473600	2.95118000	-1.90039300
H	2.03305500	3.15992300	-2.72527900
H	3.44470500	3.77829800	-1.85599100
H	3.27379900	2.03705100	-2.14110100
C	0.91494600	4.51064600	0.00483500
H	0.28670100	4.48758800	0.90277500
H	1.67790600	5.28590600	0.15160400
H	0.28259400	4.80449700	-0.83723100
C	2.83440800	2.51958700	1.23810800
H	3.64144000	1.80456800	1.06963800
H	3.27831100	3.46930000	1.56214000
H	2.22484100	2.14354000	2.06721400

2-TMS
SCF Done: -1803.051555

B	-1.79229300	1.13441100	-0.60895300
H	-1.46550900	2.11433500	-0.04086700
B	-2.49001700	-1.89192200	-1.91805000
H	-2.70150100	-2.98266100	-2.34185300
B	-3.35503600	0.34661000	-0.33180800
H	-4.10691400	0.85320900	0.43127700
B	-0.94715500	-1.07215800	-2.21992700
H	-0.00938300	-1.51868900	-2.78487000
B	-2.95440300	0.97193400	-1.94292600
H	-3.49800800	1.93314800	-2.38389300
B	-3.07305800	-1.39860900	-0.31729800
H	-3.62979800	-2.10456800	0.45483300
B	-1.34024600	-1.65772500	-0.58419300

H	-0.72226700	-2.47495700	-0.00092400
B	-3.74946500	-0.62505800	-1.76688200
H	-4.88194700	-0.81170500	-2.07807000
B	-2.43440000	-0.42240000	-2.94296700
H	-2.60075100	-0.45985100	-4.11982800
B	-1.23065000	0.67579600	-2.23583800
H	-0.47919900	1.38694800	-2.80876500
C	-1.89803300	-0.30809900	0.30380400
C	-0.63562300	-0.11267100	-0.84403300
B	0.87419400	0.13740100	-0.42254100
N	1.90141800	-0.83984300	-0.18915100
Si	2.26295600	-2.62976100	-0.16961700
Si	-1.60895200	-0.24211100	2.22644400
C	1.67607800	-3.32808600	1.47962500
H	1.91855400	-4.39656200	1.53755200
H	2.18678500	-2.82765600	2.31057200
H	0.59770800	-3.22386100	1.63150700
C	4.13003100	-2.79629400	-0.29605300
H	4.39408500	-3.85949300	-0.36108100
H	4.51056900	-2.29669700	-1.19348000
H	4.64065600	-2.35994200	0.56596300
C	1.51549900	-3.54327200	-1.63258300
H	0.42825600	-3.63019800	-1.61675600
H	1.80635800	-3.07713800	-2.57981900
H	1.93457200	-4.55830600	-1.62445000
C	0.19409500	0.04812300	2.67956000
H	0.25332200	0.07546500	3.77549600
H	0.85278100	-0.75112300	2.33298900
H	0.58424000	0.99591700	2.30315500
C	-2.66031200	1.16672500	2.90123300
H	-2.46645000	2.10668200	2.37352100
H	-3.72999700	0.95178400	2.81796200
H	-2.42687000	1.32234400	3.96156800
C	-2.16621100	-1.89455300	2.93631500
H	-1.91475500	-1.93750000	4.00310200
H	-3.24632000	-2.03831500	2.83736900
H	-1.66987400	-2.73582800	2.44072500
N	1.53276600	1.39494400	-0.19749600
N	2.83838700	1.10204100	0.19574700
N	3.04322700	-0.14026000	0.20048200
Si	1.29732300	3.20652400	-0.19185000
C	3.01196700	3.96816300	-0.28847400
H	3.54748300	3.62640900	-1.18068300
H	2.91778100	5.06009200	-0.34570400
H	3.62334100	3.71415400	0.58098800
C	0.48909700	3.69258400	1.44012100
H	-0.50125400	3.25030200	1.57995200
H	1.11952800	3.39033300	2.28436500
H	0.37620700	4.78298900	1.48600400
C	0.32202500	3.81762900	-1.67837800
H	0.76393100	3.45996400	-2.61438300
H	-0.73589400	3.55124300	-1.67936800
H	0.39235000	4.91354900	-1.68076900

TS3-TMS

SCF Done: -1802.96029

Si	1.70592500	-1.47086800	1.58210400
Si	1.47734100	-1.65589500	-1.51099700
N	0.92947700	-0.98889100	0.04974400
C	-1.68658100	-0.17990100	0.84069500
C	-1.57918600	-0.09021700	-0.74917000
C	0.52874700	-1.25203200	3.03660000
H	0.25526100	-0.21543700	3.24846800
H	1.01554200	-1.65183700	3.93528400
H	-0.39912700	-1.81197200	2.88191800
C	2.16518400	-3.30401900	1.55637000
H	1.26303700	-3.92296000	1.49102600
H	2.66894500	-3.55531800	2.49823600
H	2.83235900	-3.59497500	0.74011800
C	3.37070500	-1.71878700	-1.56296400
H	3.82587400	-0.73339200	-1.41851200
H	3.68023600	-2.08286500	-2.55080800
H	3.80496700	-2.39471600	-0.81974200
C	3.25302300	-0.41959600	1.85931200
H	3.97035300	-0.53252700	1.03955400
H	3.75767000	-0.70302700	2.79098900
H	2.99197200	0.64280800	1.93147600
B	-0.22178300	-0.14629700	0.11793900
C	0.81285900	-3.39789400	-1.79896500
H	1.00905700	-4.06608600	-0.95477800
H	1.28885700	-3.83149300	-2.68779200
H	-0.26817300	-3.38473100	-1.97156400
C	0.91253900	-0.59593000	-2.96756400
H	-0.17621200	-0.51126200	-3.02350000
H	1.25198100	-1.08568900	-3.88923800
H	1.33593300	0.41293100	-2.96395600
B	-2.00792200	-1.65422200	-0.04003500
H	-1.19038200	-2.50021900	-0.00167800
B	-4.00528500	1.07688000	-0.07363200
H	-4.69438900	2.04660900	-0.08195200
B	-2.74627400	-1.09359700	-1.53566900
H	-2.47483400	-1.63325900	-2.55829100
B	-3.13917200	0.55340700	1.42980000
H	-3.14385800	1.12287600	2.47035000
B	-3.72133600	-1.79312800	-0.18321300
H	-4.20007800	-2.87871800	-0.26931100
B	-2.92966200	0.68261100	-1.47526900
H	-2.78020700	1.33995700	-2.45278500
B	-2.29768000	1.25763100	0.05862200
H	-1.69345900	2.26525900	0.13468400
B	-4.31268300	-0.37052300	-1.07152000
H	-5.25936300	-0.43465500	-1.78963900
B	-4.44702400	-0.44816500	0.73118200
H	-5.48872900	-0.57121400	1.29309700
B	-2.96741100	-1.22099200	1.36018400
H	-2.85162500	-1.85314100	2.35799300
Si	1.32231500	2.83881400	-0.59905900
C	1.45407300	4.43746000	0.37934500
H	0.47362700	4.78793400	0.72154900
H	1.87919500	5.22179200	-0.25866100
H	2.10560600	4.33692800	1.25505000
C	3.01205000	2.17361900	-1.05571100
H	3.60642000	1.95940900	-0.16138600
H	3.55326700	2.91506700	-1.65655400
H	2.94208700	1.25444800	-1.64112900
C	0.14907300	2.98241800	-2.05035600
H	-0.22530800	2.00431100	-2.36330300
H	0.65320800	3.44551400	-2.90729400
H	-0.71894200	3.59613700	-1.79002600
N	0.56303500	1.62430200	0.55440700
N	0.26378600	2.01282700	1.69167900
N	-0.04953700	2.29462700	2.74644600

Int2-TMS

SCF Done: -1802.962109

Si	1.79724100	-1.38213800	1.55346300
Si	1.54238100	-1.53893600	-1.51393700
N	0.99688700	-0.88926600	0.04638900
C	-1.65812300	-0.22877700	0.85985000
C	-1.54760600	-0.10033800	-0.71276800
C	0.62797700	-1.25522500	3.02834800
H	0.33805700	-0.23672000	3.29899700
H	1.13040700	-1.69135200	3.90119400
H	-0.29124400	-1.82267500	2.85265700
C	2.33755900	-3.19330800	1.49726800
H	1.46141100	-3.84915500	1.43833700
H	2.86440200	-3.43174000	2.42984300
H	3.00422900	-3.44911200	0.66920700
C	3.43869900	-1.53496300	-1.59456700
H	3.86318400	-0.53479400	-1.45618100
H	3.74761000	-1.88898600	-2.58631300
H	3.90761500	-2.19375000	-0.85707900
C	3.30228700	-0.27500000	1.86607500
H	4.01870200	-0.32687100	1.03885800
H	3.82530300	-0.56775900	2.78472300
H	3.00010400	0.77383700	1.97909900
B	-0.16639100	-0.01693100	0.15131200
C	0.94898900	-3.30399300	-1.82613200
H	1.14932900	-3.97277300	-0.98361200
H	1.46122100	-3.71141800	-2.70732000
H	-0.12732700	-3.32804700	-2.02399700
C	0.93338000	-0.50561000	-2.97453500
H	-0.15754700	-0.44401100	-3.01404100
H	1.26592500	-1.00521800	-3.89348900
H	1.33855900	0.51058500	-2.99842800
B	-1.86375200	-1.70410000	-0.03545200
H	-0.99061300	-2.49066700	0.00328200
B	-4.06698300	0.86514100	-0.05158700
H	-4.82836600	1.77972100	-0.05217800
B	-2.62758600	-1.18018000	-1.52906600
H	-2.30920900	-1.68052500	-2.55837100
B	-3.17294200	0.38695900	1.45073400
H	-3.22968700	0.93861500	2.50025600
B	-3.56033900	-1.97173200	-0.19782100
H	-3.95784600	-3.08800300	-0.30499800
B	-2.95056900	0.57551900	-1.44668400
H	-2.84442700	1.25557400	-2.41478900
B	-2.38033900	1.17256700	0.09843000
H	-1.85954300	2.22700600	0.19841900
B	-4.25094200	-0.58643700	-1.07201700
H	-5.18253500	-0.71414200	-1.80178800
B	-4.39526100	-0.69974900	0.72723400
H	-5.42815600	-0.91303500	1.27882100
B	-2.86292500	-1.36552500	1.35750300
H	-2.70943200	-2.00000200	2.34915900
Si	1.13176400	2.85190800	-0.60304800

C	1.13507500	4.41808100	0.43271300
H	0.12731800	4.69559200	0.76176200
H	1.51997900	5.24568500	-0.17550800
H	1.77747400	4.33693000	1.31692400
C	2.86993400	2.30346000	-1.02062000
H	3.44726700	2.09876500	-0.11327500
H	3.38026500	3.09828000	-1.57905700
H	2.87715200	1.40151600	-1.63487000
C	-0.03269300	2.95423500	-2.06051400
H	-0.34855100	1.96451100	-2.39809900
H	0.45693700	3.46180300	-2.90052900
H	-0.93520600	3.51399000	-1.79705000
N	0.41079900	1.56303100	0.53092200
N	0.14261500	1.94088200	1.68893700
N	-0.12242700	2.23644400	2.74876600

TS4-TMS

SCF Done: -1802.954018

Si	2.08976200	-0.90894700	1.58110000
Si	1.42926900	-1.93199900	-1.28663400
N	1.12261900	-0.78238200	0.06416000
C	-1.98405100	0.14000500	1.18406700
C	-1.50144100	-0.21857800	-0.32841700
C	1.17901800	-0.09649200	3.01973000
H	1.43547000	0.96274600	3.13447400
H	1.47863800	-0.59770400	3.94787900
H	0.09040300	-0.18012500	2.92792100
C	2.29049400	-2.73702600	2.00286400
H	1.31277000	-3.22800500	2.05790200
H	2.76217500	-2.81904200	2.98999700
H	2.91125500	-3.29540600	1.29575700
C	3.30172000	-2.08721900	-1.52394100
H	3.77353100	-1.12747500	-1.76333800
H	3.47904600	-2.75800800	-2.37450100
H	3.82400000	-2.51437100	-0.66241000
C	3.78651300	-0.09492400	1.42033300
H	4.33374500	-0.40669600	0.52626200
H	4.39604700	-0.35670300	2.29463100
H	3.69780900	0.99664800	1.40212000
B	0.00158500	0.07723400	0.04597200
C	0.73792300	-3.65829700	-0.99102500
H	1.37930600	-4.23125400	-0.31364900
H	0.69238000	-4.19932200	-1.94451300
H	-0.26952300	-3.63787300	-0.57114800
C	0.77840800	-1.15818800	-2.87934200
H	-0.28397800	-0.90700700	-2.84807800
H	0.92231500	-1.86367300	-3.70692400
H	1.34586100	-0.25219400	-3.12535600
B	-1.89129200	-1.51817600	0.73655800
H	-1.00870600	-2.17358300	1.18172500
B	-4.10762700	0.64945300	-0.59255400
H	-4.85970500	1.44730800	-1.05793400
B	-2.32747600	-1.58270100	-0.98978300
H	-1.75443000	-2.32256900	-1.71751000
B	-3.62266400	0.70771100	1.13571900
H	-3.96664400	1.54347500	1.90923800
B	-3.50102500	-2.04270500	0.27298700
H	-3.82415900	-3.17945900	0.42013600
B	-2.69738700	0.07364700	-1.53029300
H	-2.35993700	0.44842300	-2.60765700
B	-2.49492000	1.14065200	-0.12446900
H	-2.04088000	2.23047900	-0.24855300
B	-4.01389700	-1.05413000	-1.12704700
H	-4.72190200	-1.49179700	-1.97950800
B	-4.59815400	-0.65569400	0.52320400
H	-5.73174700	-0.80853600	0.85586800
B	-3.24626300	-0.93232200	1.66388300
H	-3.33644400	-1.24183500	2.80835000
Si	1.13601000	2.72350100	-0.90468400
C	1.13132400	4.41103000	-0.08969100
H	0.11741100	4.76235100	0.13071500
H	1.58650100	5.13582700	-0.77576500
H	1.71398300	4.42703900	0.83824700
C	2.85368800	2.07115800	-1.24814500
H	3.55293300	2.30336400	-0.44016200
H	3.23318700	2.52824000	-2.17041200
H	2.84149500	0.98726600	-1.38398900
C	0.01730900	2.61447800	-2.39586200
H	-0.15453600	1.57902000	-2.70289300
H	0.47946300	3.14287600	-3.23925200
H	-0.96012100	3.06318200	-2.19855500
N	0.38013200	1.58763900	0.39111000
N	-0.17571200	2.15740500	1.37683600
N	-0.51381200	2.78706400	2.24784100

Int3-TMS

SCF Done: -1803.016852

Si	2.30017500	-0.65377900	1.50055600
Si	1.64813400	-1.94505600	-1.24648700
N	1.28470200	-0.76023900	0.03781600
C	-2.34918500	0.70694100	0.21175000
C	-1.42605600	-0.58603800	-0.00265300
C	1.38863000	0.24875900	2.88388600
H	0.95194800	1.20175900	2.57156800
H	2.10321600	0.45845100	3.68983900
H	0.58948000	-0.36830100	3.30681400
C	2.67064300	-2.39787400	2.12744900
H	1.74326700	-2.95685100	2.29612500
H	3.19874900	-2.32921900	3.08689100
H	3.29994900	-2.98509300	1.45074100
C	3.51526200	-2.01976800	-1.53070600
H	3.91297000	-1.04468900	-1.83290700
H	3.71667600	-2.72565500	-2.34639900
H	4.08004700	-2.36226400	-0.65792800
C	3.93998100	0.24473000	1.21657600
H	4.51413600	-0.16127200	0.37830400
H	4.55566700	0.14325900	2.11967400
H	3.79664900	1.31527000	1.03771800
B	0.07922200	0.01680900	-0.03688100
C	1.03620600	-3.67761400	-0.82133600
H	1.58973500	-4.09595900	0.02581800
H	1.17878700	-4.34957400	-1.67720000
H	-0.02593800	-3.68676900	-0.56214200
C	0.88377000	-1.33477900	-2.86084100
H	-0.19231900	-1.15621400	-2.80122500
H	1.04520400	-2.08329500	-3.64633800
H	1.36404600	-0.40531600	-3.18548700
B	-2.11623500	-0.31416600	1.57106400
H	-1.39292700	0.08423300	2.41266100
B	-3.86800900	-1.00623800	-1.25940500
H	-4.46143600	-1.24429400	-2.26071500
B	-2.08876700	-1.92136100	0.83763800
H	-1.34191700	-2.72736800	1.28257400
B	-3.87157000	0.63162500	-0.53971000
H	-4.33077000	1.62941200	-0.98169800
B	-3.57249900	-1.28429500	1.62001400
H	-3.96006000	-1.71155300	2.65855900
B	-2.27702200	-1.75404800	-0.92215300
H	-1.67677500	-2.46002300	-1.65677200
B	-2.41237600	-0.03236600	-1.32574800
H	-1.85232800	0.51684500	-2.21017600
B	-3.68006900	-2.18515500	0.07912600
H	-4.15812200	-3.27139400	0.02141500
B	-4.66503100	-0.70894000	0.31977200
H	-5.84685200	-0.73746300	0.43959700
B	-3.68658400	0.46066100	1.23220700
H	-4.02128600	1.34980400	1.93777200
Si	1.05523200	2.78416200	-0.56050600
C	1.68836300	3.68766100	0.96172000
H	0.84153900	4.05737500	1.54999500
H	2.30144600	4.55056400	0.67355100
H	2.29659700	3.04428400	1.60656500
C	2.43920400	2.01607800	-1.58111800
H	2.89142300	1.14205300	-1.11356100
H	3.22467000	2.76368700	-1.74883200
H	2.05421600	1.71599500	-2.56209800
C	0.09594000	3.93696700	-1.69970900
H	-0.49722400	3.36584100	-2.42398200
H	0.80765900	4.54905200	-2.26839400
H	-0.57799600	4.60378200	-1.15779900
N	-0.03520100	1.43624400	-0.05249800
N	-1.47891100	1.91973500	0.26508800
N	-1.80966700	3.02044700	0.53928900

TS5-TMS

SCF Done: -3032.367874

Si	-5.28798200	0.66274900	0.98755700
Si	-4.38607000	-2.04555900	-0.31641900
N	-4.02410000	-0.43343200	0.36698200
C	-0.76805900	1.15802800	-1.10877400
C	-2.37282800	1.12497400	-1.12339300
C	-4.43703800	2.17481100	1.73782900
H	-3.68547100	1.88498600	2.48236400
H	-5.19058800	2.77940700	2.25741500
H	-3.95246600	2.82273900	1.00498900
C	-6.47413900	1.15522000	-0.39270700
H	-5.94122200	1.59156500	-1.24368800
H	-7.20809400	1.88942900	-0.03823800
H	-7.03107800	0.28411700	-0.75921900
C	-5.80941400	-2.86892200	0.60767100
H	-5.65612900	-2.89525800	1.69116500
H	-5.88129400	-3.90540800	0.25458600
H	-6.77570900	-2.39288000	0.41079000
C	-6.27541700	-0.12851100	2.39111800
H	-7.04976700	-0.81549100	2.04076300
H	-6.77617100	0.67270900	2.94943100

H	-5.63412400	-0.67059900	3.09298600
B	-2.73572100	0.11949000	0.08129000
C	-4.86746300	-1.92437400	-2.13591000
H	-5.64838600	-1.17551600	-2.30657900
H	-5.24837600	-2.89389400	-2.48160500
H	-4.01206800	-1.66166500	-2.76629600
C	-2.87758500	-3.16695400	-0.17545600
H	-1.94953500	-2.70929500	-0.53187600
H	-3.05065000	-4.06767200	-0.77705900
H	-2.71958000	-3.48966100	0.85843400
B	-1.57794700	2.57373200	-0.57994300
H	-1.57968500	2.78012600	0.58365800
B	-1.47558200	1.30839800	-3.74319400
H	-1.43887600	0.84853200	-4.83687300
B	-2.98758300	2.62903200	-1.65638300
H	-4.01862800	3.01278300	-1.22255400
B	-0.06043300	1.22098100	-2.64775600
H	0.95871900	0.65627700	-2.81624700
B	-1.53804100	3.62555000	-1.97999300
H	-1.54496800	4.80319200	-1.82623500
B	-2.95306100	1.21780700	-2.73134000
H	-3.96470000	0.66573900	-2.99386800
B	-1.52340000	0.23957400	-2.35718000
H	-1.49376600	-0.93683100	-2.24382100
B	-2.38390100	2.79502800	-3.32268000
H	-3.00295000	3.39949000	-4.13629800
B	-0.59724200	2.79904500	-3.26747000
H	0.07400100	3.40375800	-4.03819600
B	-0.10265400	2.64899700	-1.57088900
H	0.88492100	3.01306300	-1.04913800
N	-1.50129100	-0.18965000	0.74536500
N	-0.31331000	0.28955800	0.02483100
N	0.80093400	-0.07264400	0.28569900
Si	3.94070000	0.32341500	2.43723400
Si	3.64789500	2.47907000	0.22302600
N	3.33280700	0.82920300	0.82840900
C	3.17235600	-1.72402000	-0.24109800
C	3.06655700	-0.77638000	-1.52577400
C	3.09013300	-1.23532300	3.06395800
H	2.01256500	-1.11117600	3.17722500
H	3.50548200	-1.47092200	4.05230700
H	3.26871000	-2.09652900	2.41498200
C	5.78951000	-0.05199700	2.38641900
H	5.98859200	-0.93375100	1.76839200
H	6.14348500	-0.27180900	3.40197700
H	6.38880200	0.77490100	1.99501400
C	2.42383900	3.67737300	1.03245300
H	1.42358800	3.24190700	1.12616400
H	2.33084100	4.60334000	0.45209900
H	2.76069700	3.94781600	2.03904600
C	3.60941500	1.69928400	3.69416700
H	4.18396100	2.61103900	3.50237000
H	3.89182300	1.34014700	4.69181000
H	2.54895000	1.97211500	3.72834100
B	2.82458200	-0.17189700	-0.03827700
C	5.39690000	3.04026900	0.66987400
H	5.59342900	3.07795900	1.74576800
H	5.55229700	4.05213200	0.27359200
H	6.14701700	2.38774500	0.20939200
C	3.57378100	2.49350100	-1.65867100
H	4.42741100	1.93859200	-2.06373200
H	3.64238700	3.52691800	-2.02046000
H	2.67082200	2.05010100	-2.07850600
B	4.67651100	-1.20875600	-1.00599600
H	5.31972000	-0.37514100	-0.47510000
B	2.43952600	-3.36864800	-2.34513300
H	1.64127000	-4.11648600	-2.81330100
B	4.27197400	-1.14408300	-2.71201800
H	4.72837100	-0.27917200	-3.38391500
B	2.78761200	-3.35895000	-0.57124700
H	2.24723800	-4.01863000	0.25501100
B	5.18321400	-2.53301200	-1.99180800
H	6.34469100	-2.68237300	-2.20082500
B	2.57894500	-1.66154900	-2.93095300
H	1.89601300	-1.16025900	-3.75928700
B	1.90281400	-2.05800100	-1.35845700
H	0.79530600	-1.78780700	-1.04979600
B	3.90635500	-2.82215100	-3.19948200
H	4.16230500	-3.21147800	-4.29450200
B	4.03976500	-3.87471900	-1.73267400
H	4.38958700	-5.01074200	-1.79020200
B	4.48420000	-2.83438600	-0.35230100
H	5.08504400	-3.14447800	0.62391200
Si	-1.09417200	-0.90222300	2.37887100
C	-2.72112600	-1.22401600	3.26202700
H	-3.29175000	-0.30442400	3.41758800
H	-3.35908500	-1.93199900	2.72889600
H	-2.49296800	-1.64660200	4.24926800
C	-0.13643200	-2.50731500	2.21658000
H	-0.76269400	-3.29288500	1.78435600
H	0.75370500	-2.40307800	1.59466200
H	0.18294900	-2.84642800	3.20987300
C	-0.19552200	0.48854100	3.27812600
H	0.68117600	0.84743100	2.73368400
H	-0.87951500	1.33365900	3.42262600
H	0.13945400	0.15850800	4.26852000

Int4-TMS

SCF Done: -3032.377219

Si	-5.17144800	0.65509400	0.99425200
Si	-4.27775900	-1.98462100	-0.46845000
N	-3.90908400	-0.42103000	0.32681900
C	-0.61475200	1.18565500	-1.03511100
C	-2.22774200	1.20602000	-1.03834900
C	-4.30327000	2.07790400	1.88724600
H	-3.62874400	1.70333200	2.66674200
H	-5.06187100	2.69344900	2.38609800
H	-3.72998300	2.73965400	1.23557500
C	-6.31259400	1.27853200	-0.37054700
H	-5.75488100	1.73894900	-1.19198600
H	-7.01605600	2.02308400	0.02201900
H	-6.90519600	0.45583800	-0.78913000
C	-5.69052800	-2.87119200	0.41070800
H	-5.53575300	-2.95007300	1.49138500
H	-5.74896900	-3.88948200	0.00604100
H	-6.66297100	-2.39848400	0.23821700
C	-6.21230400	-0.21529700	2.30778400
H	-6.97578200	-0.87481300	1.88833200
H	-6.72947600	0.55521700	2.89388000
H	-5.60029700	-0.80207200	2.99961800
B	-2.61963900	0.13470100	0.08318100
C	-4.77472100	-1.72581000	-2.26836900
H	-5.55411700	-0.96396600	-2.37576900
H	-5.16299000	-2.66584600	-2.68059700
H	-3.92428000	-1.42138700	-2.88640700
C	-2.76812000	-3.11073700	-0.41637800
H	-1.83604100	-2.63474300	-0.73592800
H	-2.94328900	-3.96272800	-1.08466200
H	-2.61299400	-3.51328500	0.58926600
B	-1.38995700	2.58603900	-0.39085400
H	-1.37312300	2.69732800	0.78531000
B	-1.36452900	1.57231500	-3.64535600
H	-1.35185600	1.19869100	-4.76966100
B	-2.81227500	2.76068200	-1.43927400
H	-3.82567100	3.12793800	-0.95396200
B	0.06496500	1.36634900	-2.58686000
H	1.06600600	0.81029600	-2.83946700
B	-1.34417500	3.74222200	-1.70170500
H	-1.31605300	4.90252800	-1.45174900
B	-2.82723800	1.43771200	-2.62096900
H	-3.85477700	0.92882800	-2.90778500
B	-1.41617600	0.39496700	-2.34692200
H	-1.42001700	-0.78606500	-2.32676000
B	-2.22871700	3.04265400	-3.09382700
H	-2.84299700	3.72396800	-3.84772800
B	-0.44470200	2.99991100	-3.06524800
H	0.23623700	3.64485700	-3.79285200
B	0.07047800	2.70182300	-1.39513400
H	1.06934500	3.01180500	-0.86910000
N	-1.38375100	-0.23780400	0.73271600
N	-0.21622000	0.23572300	0.05960700
N	0.87685400	-0.20395700	0.37046400
Si	3.79113600	0.14662300	2.40533800
Si	3.54597300	2.40973400	0.37631100
N	3.09739200	0.76441700	0.87781000
C	2.95772300	-1.70091300	-0.35025100
C	2.78512300	-0.71053500	-1.56966700
C	2.98765300	-1.45196800	3.00060500
H	1.93503900	-1.33914400	3.25975800
H	3.51946700	-1.75943300	3.91039900
H	3.08142900	-2.26284300	2.27438100
C	5.63410700	-0.25919300	2.29008200
H	5.80719000	-1.08850800	1.59776800
H	5.97744900	-0.57962400	3.28276200
H	6.26133700	0.57848800	1.97692400
C	2.39909900	3.66071300	1.23247000
H	1.37367500	3.29047500	1.33455100
H	2.35974200	4.61362000	0.69056500
H	2.77191500	3.86854200	2.24203700
C	3.53861000	1.43112500	3.77846500
H	4.12955700	2.34151200	3.63809100
H	3.84707100	0.98914000	4.73452800
H	2.48857900	1.72814000	3.87450100
B	2.40396300	-0.18291800	-0.02574500
C	5.30950200	2.87775000	0.88371100
H	5.50606500	2.86738100	1.95923300

H	5.48599000	3.90229700	0.52940800
H	6.04437200	2.23162800	0.39234500
C	3.58898700	2.55877100	-1.50558900
H	4.51869600	2.10657500	-1.86803800
H	3.59723400	3.61774400	-1.79280100
H	2.77227400	2.07195700	-2.03628300
B	4.41029800	-1.06144900	-1.08841300
H	5.01346600	-0.21952600	-0.52710900
B	2.32089900	-3.30436700	-2.52290700
H	1.56919500	-4.07975300	-3.02387800
B	3.99536900	-0.94565500	-2.78560500
H	4.38856300	-0.02427500	-3.42200600
B	2.67735000	-3.34802600	-0.75339600
H	2.18786600	-4.08046100	0.04394800
B	5.00316400	-2.30159100	-2.13740800
H	6.17024900	-2.36764700	-2.35981300
B	2.34428700	-1.57047400	-3.02550900
H	1.62235800	-1.08314300	-3.83008300
B	1.70934300	-2.07658800	-1.47479400
H	0.58464600	-1.89276000	-1.15011200
B	3.74236100	-2.62206000	-3.35412400
H	4.01966600	-2.93810600	-4.46792600
B	3.95341800	-3.72975000	-1.93960000
H	4.38002100	-4.83592800	-2.04807000
B	4.33325400	-2.72392700	-0.51440300
H	4.95763900	-3.04727000	0.44256900
Si	-1.04517000	-1.06316800	2.34638300
C	-2.70330000	-1.41679500	3.15380100
H	-3.27565700	-0.50645200	3.34729900
H	-3.32875900	-2.09763000	2.57282500
H	-2.49564800	-1.89087800	4.12251600
C	-0.12594100	-2.68061500	2.11887100
H	-0.78149500	-3.43394900	1.67326400
H	0.76150600	-2.59012400	1.49266700
H	0.19134600	-3.05731900	3.09892200
C	-0.18942200	0.28945900	3.33607500
H	0.68798300	0.69556900	2.82900400
H	-0.89825300	1.10869300	3.50806900
H	0.13286500	-0.08261700	4.31528200

TS6-TMS

SCF Done: -3032.36785

Si	-5.05086700	0.13942600	1.23831800
Si	-3.77709300	-2.24603500	-0.32526400
N	-3.60706200	-0.66362100	0.52565400
C	-0.85818400	1.71051100	-0.87567100
C	-2.42027200	1.42732500	-0.72169700
C	-4.51521700	1.68325100	2.20478200
H	-4.52705200	1.46311700	3.27844100
H	-5.23180400	2.49437000	2.03171500
H	-3.52652900	2.07733900	1.96328800
C	-6.30680500	0.57296500	-0.09874900
H	-5.90941900	1.28986300	-0.82294300
H	-7.20261800	1.01377600	0.35647500
H	-6.62411400	-0.31954300	-0.65095500
C	-5.27003500	-3.21396800	0.29817700
H	-5.21385100	-3.45373100	1.36407500
H	-5.27473300	-4.16268700	-0.25375200
H	-6.22773500	-2.72228500	0.10177000
C	-5.89937200	-0.94750800	2.53187100
H	-6.58754500	-1.68578900	2.11578800
H	-6.48560500	-0.27967900	3.17666400
H	-5.18093700	-1.46831500	3.17375800
B	-2.46061900	0.12345200	0.21951600
C	-4.04557700	-1.94995600	-2.16737700
H	-4.90428800	-1.29398100	-2.34734800
H	-4.24603200	-2.90801500	-2.66320900
H	-3.17660300	-1.50394200	-2.65893700
C	-2.27327300	-3.33956400	-0.06710900
H	-1.32498200	-2.84954100	-0.29413200
H	-2.35638000	-4.20902500	-0.73074500
H	-2.23626000	-3.71547600	0.96112200
B	-1.74690200	2.85296500	0.02501500
H	-1.60021000	2.82435800	1.19800800
B	-1.94722600	2.26214700	-3.32818000
H	-2.01052100	2.03391800	-4.49110500
B	-3.29381600	2.89646600	-0.84606400
H	-4.28979600	3.02892300	-0.22478700
B	-0.38873600	2.17451700	-2.45470700
H	0.64760200	1.79237200	-2.86899900
B	-2.04018900	4.14709900	-1.11334200
H	-2.17801500	5.25579500	-0.71098100
B	-3.23335600	1.74133000	-2.19659900
H	-4.18249800	1.08565900	-2.45590600
B	-1.65328300	0.95002800	-2.21242500
H	-1.43613600	-0.20060100	-2.35142500
B	-2.96480500	3.47427000	-2.49461000
H	-3.76723000	4.12565600	-3.07951900
B	-1.20014200	3.74360400	-2.64900100
H	-0.73239500	4.58481600	-3.34421200
B	-0.44132100	3.34289500	-1.09277900
H	0.56777800	3.73891900	-0.62652000
N	-1.07934700	-0.14143800	0.63040700
N	-0.14411000	0.71152300	-0.05775900
N	1.08217400	0.67959500	-0.14175100
Si	4.35757600	-0.14023300	2.02724400
Si	3.70696500	2.28807700	0.26405800
N	3.36389800	0.59729300	0.74343200
C	2.53466300	-1.66061100	-0.53933800
C	2.33780900	-0.71841100	-1.80282000
C	3.56303700	-1.68534400	2.75577900
H	2.63397700	-1.44722000	3.28072400
H	4.26120800	-2.09347300	3.49791000
H	3.36495700	-2.47381300	2.02701500
C	6.08767200	-0.62157300	1.44968600
H	6.03559500	-1.43358200	0.71753900
H	6.66786000	-0.98208100	2.30898400
H	6.63985200	0.20609300	0.99689500
C	2.70176500	3.46684100	1.35010400
H	1.64082700	3.19703900	1.35735800
H	2.78059500	4.50113100	0.99321100
H	3.05748300	3.44145400	2.38644000
C	4.46277100	1.06141800	3.49098800
H	4.96401300	2.00910900	3.27664200
H	5.01545200	0.57562100	4.30523500
H	3.45946400	1.29410400	3.86699400
B	2.35594900	-0.15015200	0.01248000
C	5.54323500	2.70754900	0.46044800
H	5.96248400	2.56381100	1.45937600
H	5.66840300	3.76765000	0.20365300
H	6.14733900	2.12969400	-0.24797200
C	3.36599900	2.54765200	-1.56625600
H	3.94819600	1.83973100	-2.16602800
H	3.67343800	3.56251900	-1.84827600
H	2.31822600	2.42210200	-1.83107200
B	3.94020600	-1.26158200	-1.52899600
H	4.73455100	-0.49676300	-1.11362400
B	1.37179000	-3.22897600	-2.51279700
H	0.44825500	-3.90384900	-2.84400300
B	3.30289400	-1.16978800	-3.15586900
H	3.72281000	-0.33245400	-3.88544100
B	1.98450900	-3.27620800	-0.82545300
H	1.52474500	-3.91707000	0.06084000
B	4.19643400	-2.62273600	-2.57623400
H	5.29946800	-2.86737100	-2.94959200
B	1.56457900	-1.53894700	-3.11322800
H	0.80002100	-0.96027600	-3.81443400
B	1.11430700	-1.88097600	-1.45578900
H	0.09794600	-1.51581800	-0.99021500
B	2.73263200	-2.80642600	-3.58536800
H	2.79270000	-3.20531700	-4.70548900
B	3.00049000	-3.87120300	-2.15228900
H	3.25023100	-5.03105000	-2.25294000
B	3.72502600	-2.88223000	-0.85377200
H	4.44163400	-3.25895700	0.01412500
Si	-0.58339700	-0.72447300	2.31190700
C	-2.13421000	-0.87850200	3.35496300
H	-2.68225600	0.06260000	3.44144400
H	-2.81414900	-1.64163200	2.97211000
H	-1.81644800	-1.17569500	4.36300400
C	0.26803800	-2.38147500	2.26359300
H	-0.41625000	-3.16996500	1.94560300
H	1.12720100	-2.39769600	1.59768000
H	0.62045000	-2.62851900	3.27200800
C	0.47950100	0.66223500	3.00485000
H	1.42063500	0.81429200	2.47290500
H	-0.07210200	1.60988600	3.00089600
H	0.72428000	0.42659800	4.04796300

3-TMS

SCF Done: -3032.432206

Si	4.99371900	-0.91043400	-0.54132000
Si	3.49393000	-1.33418500	2.09310800
N	3.46547000	-0.75943500	0.39444600
C	0.71820700	1.59947000	-1.16625400
C	2.31845900	1.37058800	-0.84438400
C	4.64667000	-1.12283400	-2.39283100
H	4.68649100	-2.18740500	-2.64938900
H	5.41945800	-0.61167900	-2.97832100
H	3.68261700	-0.73664500	-2.72714500
C	6.16706000	0.53276800	-0.22955700
H	5.72063600	1.49962600	-0.47195300
H	7.06385300	0.41258400	-0.85103200
H	6.48973200	0.56518500	0.81598200
C	3.43152300	-3.21630200	2.27814500
H	2.47155700	-3.62839400	1.95040900

H	3.53976500	-3.45492300	3.34428400
H	4.22492400	-3.74338700	1.74174900
C	5.90085000	-2.50316200	-0.06732600
H	6.21425800	-2.57676500	0.97697900
H	6.80919900	-2.54592800	-0.68284100
H	5.30444400	-3.38891900	-0.31201500
B	2.32363400	-0.06787500	-0.13692500
C	5.04491000	-0.67814100	2.95315700
H	5.98857500	-1.07754300	2.57185800
H	4.98557400	-0.93616800	4.01816900
H	5.08698000	0.41470100	2.88056200
C	2.04407700	-0.61801300	3.03374600
H	2.15509700	0.46459100	3.13766600
H	2.01252600	-1.04432100	4.04380400
H	1.08368700	-0.81040900	2.55471400
B	1.78046300	1.26688600	-2.48702000
H	1.70718000	0.18937000	-2.96785700
B	1.81400600	4.08374600	-0.60589800
H	1.80069600	5.05601200	0.07638300
B	3.29985500	2.07895000	-2.04845700
H	4.31941600	1.56111200	-2.33992300
B	0.29145500	3.23313100	-1.04858600
H	-0.80216700	3.50408300	-0.70257300
B	2.17714800	2.82925000	-3.19711000
H	2.42972800	2.89788500	-4.35647400
B	3.10226600	2.85201300	-0.46522900
H	3.97169400	2.82583600	0.34020700
B	1.46958800	2.52437800	0.09712000
H	1.22015000	2.20655400	1.19832800
B	2.97532300	3.82416500	-1.94233800
H	3.81029700	4.62246300	-2.22162100
B	1.24334900	4.06474900	-2.30159500
H	0.81604500	5.04287800	-2.82483300
B	0.52516900	2.49017700	-2.65186900
H	-0.38798000	2.27762300	-3.35965900
N	0.95941800	-0.54775500	-0.18725600
N	-0.02122500	0.42879300	-0.69954300
N	-1.14896600	0.63233200	0.09804600
Si	-4.16346100	-2.25695800	-0.18166600
Si	-3.89633500	-0.00652800	-2.24883300
N	-3.29579400	-0.78289100	-0.73812300
C	-2.81223300	0.67558600	1.56022700
C	-1.36268100	1.37134900	1.41954400
C	-3.95952000	-2.60282200	1.65857500
H	-4.15892700	-3.67096100	1.81335800
H	-4.69747800	-2.04247300	2.23798000
H	-2.97725700	-2.39129800	2.08538500
C	-6.02825600	-2.11633200	-0.44739100
H	-6.43755100	-1.27078900	0.11670900
H	-6.49581700	-3.02926000	-0.05665200
H	-6.33697400	-2.01103800	-1.49090000
C	-2.56573000	1.11496800	-2.91203900
H	-2.33912300	1.93892300	-2.23123800
H	-2.87454300	1.55056000	-3.86973300
H	-1.64382000	0.55354700	-3.07671800
C	-3.49570000	-3.74376100	-1.13347400
H	-3.57273500	-3.62915300	-2.21785500
H	-4.06384700	-4.63813000	-0.84787800
H	-2.44452300	-3.92625300	-0.89184200
B	-2.48468300	-0.01022100	0.12374600
C	-4.26382200	-1.29925300	-3.57462900
H	-3.35222700	-1.84030400	-3.85207400
H	-4.61904800	-0.77308100	-4.46983400
H	-5.02721000	-2.03364000	-3.30375700
C	-5.42807400	1.02093600	-1.85416200
H	-6.30330100	0.41781400	-1.59776500
H	-5.69010100	1.64010400	-2.72148000
H	-5.22870100	1.69642200	-1.01376900
B	-2.78240400	2.35599700	1.11201300
H	-2.99993300	2.61018900	-0.02154400
B	-1.89334700	1.08803600	4.12792900
H	-1.57167900	0.63962300	5.18066200
B	-1.39784000	3.00038300	1.99765400
H	-0.71256200	3.83364300	1.52460800
B	-3.25982500	0.41970800	3.19533100
H	-3.89473100	-0.52819100	3.49750400
B	-3.08932400	3.23381400	2.57737800
H	-3.60758900	4.30206200	2.52815700
B	-0.66250300	1.67380000	2.95182200
H	0.50152500	1.64683400	3.10941200
B	-1.58371100	0.18948100	2.67038500
H	-1.08810300	-0.86389100	2.48095600
B	-1.77768600	2.82793500	3.72341200
H	-1.36110100	3.62208500	4.50370000
B	-3.38259300	2.05154300	3.88063700
H	-4.12485800	2.28475700	4.77909400
B	-3.98784600	1.72154600	2.23167800
H	-5.10903300	1.62905000	1.85291700
Si	0.55665700	-2.25530000	-0.66181400
C	-0.49527400	-2.17665900	-2.21515200
H	-1.47944700	-1.74939200	-2.03137200
H	0.01029800	-1.57171500	-2.97576500
H	-0.62533400	-3.18879300	-2.61715800
C	-0.30386600	-3.09976900	0.78076800
H	-0.45418700	-4.16695500	0.57843000
H	0.28903800	-3.01246900	1.69731500
H	-1.27996200	-2.65799400	0.98148400
C	2.08079600	-3.25548800	-1.12668200
H	2.54655900	-2.86129500	-2.03392900
H	2.84097100	-3.31865500	-0.34990700
H	1.73655300	-4.27426700	-1.34967100

TS7

SCF Done: -3032.369864

Si	5.23152700	-0.17811500	-1.27747100
Si	3.54800600	-2.49576900	-0.02227100
N	3.67694700	-0.75440900	-0.52928000
C	1.26869300	1.98254700	0.74101000
C	2.83302500	1.55179300	0.61499400
C	4.85954800	1.25623700	-2.44037400
H	4.29516600	0.90051700	-3.30874800
H	5.81061500	1.66212600	-2.80755200
H	4.30975600	2.08520100	-1.99238800
C	6.49872300	0.28527200	0.03315200
H	6.14962800	1.08967500	0.68479300
H	7.41955400	0.62771400	-0.45565600
H	6.75535100	-0.57155900	0.66384800
C	3.31668300	-3.69074000	-1.45758100
H	2.36997600	-3.53784100	-1.98065500
H	3.30016000	-4.70790500	-1.04484200
H	4.12424000	-3.64843100	-2.19301000
C	5.92180800	-1.54147100	-2.38208800
H	6.21547100	-2.45483200	-1.85856700
H	6.81941800	-1.14198400	-2.87130300
H	5.21102200	-1.80736400	-3.17172200
B	2.70621400	0.18267600	-0.19197500
C	5.12292200	-2.93167100	0.92046000
H	6.03103800	-2.94845900	0.31125900
H	4.99411400	-3.93604900	1.34387000
H	5.28307400	-2.24178800	1.75643000
C	2.12988900	-2.62862100	1.18995000
H	2.39227300	-2.16702400	2.14738200
H	1.90150700	-3.68219700	1.38817100
H	1.20129800	-2.16834500	0.85026600
B	2.28166100	2.97647600	-0.22921700
H	2.14832800	2.87864800	-1.40025800
B	2.41777800	2.59630400	3.15185800
H	2.45541900	2.44534300	4.32823800
B	3.82948500	2.94039500	0.65119100
H	4.82856600	2.94384600	0.01975300
B	0.85234400	2.61199000	2.27182900
H	-0.21648000	2.37499100	2.71003400
B	2.69236400	4.31569500	0.81720400
H	2.93539100	5.37602300	0.34146800
B	3.66393400	1.88782900	2.07161300
H	4.54424100	1.16220800	2.39074600
B	2.01298400	1.24926300	2.12208400
H	1.69963000	0.13250900	2.33563300
B	3.54822500	3.65779500	2.25265100
H	4.40577300	4.26933300	2.80071900
B	1.82104300	4.10203000	2.36936600
H	1.44119400	5.02639500	3.00997800
B	1.02346500	3.66544000	0.83963100
H	0.05825600	4.11761300	0.32555400
N	1.16434200	0.11735500	-0.50362600
N	0.48016200	1.09804800	-0.07388400
N	-1.52260600	-0.71235400	-0.04112600
Si	-4.72369900	-0.22245100	-2.05242200
Si	-3.87962000	2.33533800	-0.54115400
N	-3.66001800	0.61369700	-0.90346900
C	-3.26084800	-0.96890600	1.41657500
C	-1.72336200	-1.46785800	1.19614300
C	-3.85804500	-1.71339600	-2.82385900
H	-4.50113700	-2.12666600	-3.61138300
H	-3.66985400	-2.51171000	-2.10204200
H	-2.90473300	-1.43687700	-3.28432500
C	-6.29254300	-0.80976600	-1.18472600
H	-6.05002000	-1.50022300	-0.36884400
H	-6.96167300	-1.33161600	-1.88045000
H	-6.84525900	0.03090400	-0.75094700
C	-3.16520700	3.39859800	-1.93265900
H	-2.09389300	3.19768700	-2.04970800
H	-3.28148800	4.46272700	-1.69050000
H	-3.64528500	3.22104500	-2.89956200
C	-5.16992000	0.91936200	-3.49387900
H	-5.68087700	1.84048000	-3.19807600

H	-5.84072000	0.37681300	-4.17198800
H	-4.27597200	1.19563600	-4.06480600
B	-2.85963100	-0.19872400	-0.01683300
C	-5.70573500	2.75237100	-0.27448000
H	-6.33701700	2.54055400	-1.14360300
H	-5.81351800	3.81906200	-0.04033000
H	-6.10449700	2.18339500	0.57376400
C	-2.95379400	2.80107100	1.02971200
H	-3.35041100	2.29933100	1.91751600
H	-3.03985200	3.88367600	1.18656400
H	-1.89034800	2.55978200	0.95027400
B	-2.05762900	-0.35992600	2.49528000
H	-1.67417700	0.74024200	2.33529200
B	-3.12835900	-3.56181700	2.39674500
H	-3.50089000	-4.69094000	2.34638500
B	-0.93042700	-1.69156900	2.70209800
H	0.22248800	-1.45705900	2.81738600
B	-4.24595900	-2.17787900	2.12584600
H	-5.38715300	-2.26237700	1.80920700
B	-2.17969200	-1.30606200	3.94498500
H	-1.87156500	-0.83436400	4.99339600
B	-1.51003900	-3.08079900	1.74154200
H	-0.74854000	-3.80141200	1.18544100
B	-3.01332200	-2.61502900	0.94742400
H	-3.24038600	-2.90962300	-0.17296800
B	-1.83465800	-3.00308100	3.49499200
H	-1.29080300	-3.74831100	4.24794800
B	-3.52764800	-2.45309400	3.73780000
H	-4.18694200	-2.81118200	4.66111200
B	-3.66464600	-0.79752900	3.06969500
H	-4.40510900	0.07026200	3.40130200
Si	0.18523500	-0.79876800	-1.91220900
C	-0.89424600	0.47525900	-2.76216800
H	-1.84089700	0.67773700	-2.26938900
H	-0.32851600	1.41196000	-2.83988900
H	-1.08213700	0.13266100	-3.78801600
C	-0.23363400	-2.62653200	-1.79456000
H	0.14011500	-3.15904400	-2.67741600
H	0.19229400	-3.09634200	-0.90492600
H	-1.31643800	-2.74048900	-1.72422500
C	1.68752800	-0.75596900	-3.11566500
H	2.08165300	0.25852800	-3.24863300
H	2.51968700	-1.40761200	-2.84364800
H	1.31147400	-1.08205100	-4.09505700

Int5

SCF Done: -1338.891533

Si	-2.73695100	-1.29079100	0.21394400
Si	-1.84014300	1.77318000	-0.11704900
N	-1.47751000	0.00471500	-0.02364700
C	2.16909600	-1.12449300	-0.55452800
C	1.27512900	0.12139200	0.03415200
C	-1.94952000	-2.75478400	1.10323900
H	-1.23875800	-3.29792800	0.47601900
H	-2.73875800	-3.45403300	1.40716600
H	-1.43183400	-2.43085800	2.01454300
C	-4.08621600	-0.65447500	1.37615200
H	-3.68741800	-0.00714500	2.16575300
H	-4.53898900	-1.52378100	1.86884300
H	-4.88461800	-0.11486700	0.86161900
C	-3.69230400	2.09014600	-0.29226500
H	-4.17997200	1.41696500	-1.00567000
H	-3.80278900	3.10942400	-0.68438700
H	-4.23445200	2.04284600	0.65536200
C	-3.44477000	-1.77567800	-1.45809300
H	-3.89512500	-0.91742100	-1.97077300
H	-4.21884800	-2.54543500	-1.35103300
H	-2.64798500	-2.17589500	-2.09363500
B	-0.18700600	-0.45343000	-0.27328600
C	-1.22462700	2.67044800	1.41594500
H	-1.60302100	2.18799500	2.32494000
H	-1.59279500	3.70406200	1.40889100
H	-0.13485100	2.70319700	1.48144500
C	-1.07142600	2.41051100	-1.71601000
H	-0.01284000	2.17063400	-1.83258800
H	-1.17034100	3.50183500	-1.77016300
H	-1.60798500	1.98680800	-2.57376900
B	1.98886000	-1.00600700	1.15159700
H	1.25663600	-1.78456700	1.66087800
B	3.70562200	1.11675100	-0.85970700
H	4.29119500	1.86185700	-1.57625000
B	2.00818200	0.73837200	1.45673100
H	1.30926800	1.16064100	2.31487100
B	3.67192300	-0.64722900	-1.17492600
H	4.09828400	-1.21901500	-2.12059700
B	3.47545700	-0.26143600	1.69307000
H	3.89701000	-0.49526200	2.77898600
B	2.15351000	1.58071400	-0.09973800
H	1.56278400	2.58825800	-0.28985900
B	2.21573700	0.36490100	-1.39971800
H	1.62393700	0.42092800	-2.42289100
B	3.59413400	1.34713400	0.91512600
H	4.10661500	2.27116900	1.45859000
B	4.52963400	-0.02368600	0.25661900
H	5.71391400	-0.08000300	0.33408700
B	3.53211100	-1.49110500	0.38862700
H	3.86054500	-2.62539500	0.48095400
N	0.04709300	-1.80958900	-0.97638900
N	1.24291900	-2.11394800	-1.15444700

4

SCF Done: -1693.542194

Si	-2.40162100	-1.09327600	-1.37037800
Si	-2.34384800	-0.03064000	1.56372900
N	-1.57904900	-0.24528000	-0.03465400
C	1.10597100	-1.16834300	-0.02621800
C	1.92864900	0.22211100	0.05705300
C	-1.17008100	-1.38404700	-2.76667200
H	-0.71944900	-0.44904300	-3.11844600
H	-1.70075600	-1.83097200	-3.61658600
H	-0.36043700	-2.06467600	-2.48834400
C	-3.08595800	-2.75529700	-0.79325600
H	-2.28204800	-3.38137100	-0.38869300
H	-3.54075200	-3.29430600	-1.63359400
H	-3.85128700	-2.65031500	-0.01617000
C	-4.19671400	0.27147200	1.37665900
H	-4.40321400	1.15907400	0.76892500
H	-4.62394600	0.44300900	2.37264800
H	-4.73038500	-0.57641400	0.93516700
C	-3.78353200	-0.00454300	-2.05620900
H	-4.64537000	0.07274000	-1.38743000
H	-4.13799900	-0.41725200	-3.00916800
H	-3.41474300	1.00997100	-2.24788600
B	-0.16891300	-0.10885700	-0.09806300
C	-2.05542500	-1.58668900	2.59131400
H	-2.51795800	-2.46398600	2.12549300
H	-2.47569600	-1.47925600	3.59908600
H	-0.98459700	-1.79565400	2.69612000
C	-1.60581200	1.44909600	2.47001800
H	-0.51590100	1.39875700	2.53697100
H	-2.00204400	1.47353700	3.49282300
H	-1.87283000	2.39612700	1.98964500
B	1.74406400	-0.70554400	1.52400400
H	0.97520800	-0.27758900	2.31338900
B	3.59466100	-1.60361900	-1.16652100
H	4.24261600	-1.91665900	-2.11265900
B	3.24157400	0.14752500	1.13651700
H	3.54240100	1.10969400	1.76238000
B	2.02307400	-2.39261500	-0.78906700
H	1.47210400	-3.20460600	-1.45607400
B	3.23064700	-1.57247800	1.71496500
H	3.61900300	-1.86047100	2.80093900
B	3.47650200	0.12247800	-0.64397800
H	3.94474900	1.05953400	-1.19598200
B	2.11111200	-0.72946700	-1.37817500
H	1.56310600	-0.31680300	-2.34009000
B	4.31593000	-1.06498400	0.38562200
H	5.49431200	-1.01018500	0.53531100
B	3.43045300	-2.62724500	0.28842200
H	3.98063700	-3.67786500	0.36925300
B	1.79889700	-2.37495400	0.97297200
H	1.08570300	-3.16504200	1.49794900
Si	0.62039900	2.77776800	-0.42650300
C	1.83795400	3.18678600	-1.80350100
H	2.87713700	3.12133500	-1.46638300
H	1.66803500	4.20989900	-2.16165000
H	1.72013200	2.50726900	-2.65500800
C	-1.14172100	3.08713600	-1.01119200
H	-1.32899000	2.55375100	-1.95029900
H	-1.30200300	4.15642300	-1.19431700
H	-1.88660800	2.74747200	-0.28706900
C	1.06527800	3.75054300	1.12176700
H	0.33158100	3.60490200	1.92065900
H	1.12544900	4.82431700	0.90550200
H	2.04359300	3.43370300	1.50337800
N	0.71913100	1.03620600	-0.08359400

TS8

SCF Done: -1338.850684

Si	-2.44062900	-1.38015500	0.51883900
Si	-2.10812300	1.73536200	-0.25397200
N	-1.42525400	0.04109100	-0.05999600
C	2.52045700	-0.75470400	-1.05911500
C	1.35631700	-0.06339000	-0.08706100
C	-1.26852300	-2.72116000	1.10965600
H	-0.69726800	-3.17465400	0.29462400

H	-1.85681200	-3.51619800	1.58517100
H	-0.55754700	-2.34572100	1.85350700
C	-3.45702300	-0.77429100	1.98062400
H	-2.80474300	-0.40703000	2.78053500
H	-4.02923100	-1.61965500	2.38296500
H	-4.17139600	0.01568900	1.73101500
C	-3.86520000	1.57595500	-0.90867100
H	-3.88280900	1.07482100	-1.88249200
H	-4.26741300	2.58690000	-1.05187800
H	-4.54698800	1.04663200	-0.23690000
C	-3.49277400	-1.97770800	-0.91512300
H	-4.26750000	-1.25945400	-1.19885800
H	-3.98896100	-2.91727800	-0.64205800
H	-2.86438300	-2.17317600	-1.79042200
B	-0.13824200	-0.21070400	-0.30861200
C	-2.05832100	2.56260700	1.42837600
H	-2.71903100	2.06529200	2.14566900
H	-2.37817400	3.60867400	1.34536900
H	-1.04251300	2.55251400	1.83652300
C	-1.07932900	2.63014500	-1.54103300
H	-0.05480900	2.82207100	-1.21485400
H	-1.54756200	3.59862300	-1.75741800
H	-1.03792100	2.06717700	-2.48053400
B	2.20682800	-1.41833400	0.48179400
H	1.64197100	-2.45710800	0.57944900
B	3.47342500	1.68249500	-0.05809900
H	3.89767800	2.77145100	-0.28323500
B	1.80311400	-0.02327300	1.57601400
H	0.93347100	-0.15655300	2.37843800
B	3.88034000	0.26803400	-1.07802100
H	4.52853200	0.31804400	-2.07285600
B	3.42492200	-0.72383100	1.59396600
H	3.81334900	-1.34429900	2.53236200
B	1.83736000	1.45299300	0.56598500
H	1.00646600	2.28512700	0.72904400
B	2.25140600	0.92948400	-1.13212200
H	1.70722300	1.40920400	-2.07256500
B	3.20685200	1.05978200	1.61290300
H	3.42073100	1.72459200	2.57727700
B	4.48188100	0.34689800	0.60692600
H	5.63793100	0.49575700	0.84808100
B	3.85462600	-1.17401300	-0.08258400
H	4.47528100	-2.14222700	-0.38248300
N	-0.06404500	-1.75368400	-1.94317300
N	0.91626900	-1.96090400	-2.45087000

N2

SCF Done: -109.550112

N	0.00000000	0.00000000	0.55275100
N	0.00000000	0.00000000	-0.55275100

TS9

SCF Done: -1802.943165

Si	2.01798400	-1.03018400	1.60353400
Si	1.60802500	-1.72722200	-1.38550200
N	1.12092800	-0.78129600	0.06478200
C	-1.54353600	-0.26667100	0.42236000
C	-1.97314000	0.00112900	-1.13701900
C	0.93631800	-0.51895400	3.06971800
H	0.91837800	0.56008600	3.25422800
H	1.36162300	-0.97772700	3.97123800
H	-0.09603700	-0.87519200	2.98680600
C	2.40903200	-2.85922400	1.83366900
H	1.48302800	-3.43394200	1.94415600
H	2.98896500	-2.97980400	2.75731600
H	2.98477000	-3.30539600	1.01837000
C	3.49902600	-1.65073900	-1.52987400
H	3.89412700	-0.63259600	-1.60295900
H	3.78172100	-2.17814800	-2.45031300
H	4.01785600	-2.15064400	-0.70470700
C	3.59512100	0.00584000	1.66307100
H	4.24689500	-0.16528900	0.80127400
H	4.16486400	-0.23181700	2.57019000
H	3.35466300	1.07476000	1.69901100
B	-0.01787600	0.04035700	0.25439100
C	1.16079500	-3.56529800	-1.27489500
H	0.70658300	-3.82745200	-0.31610700
H	2.05727300	-4.18370700	-1.40552900
H	0.44504600	-3.83692100	-2.05791900
C	0.87819500	-0.93098800	-2.92247800
H	-0.19854000	-0.76940200	-2.82016400
H	1.05607600	-1.58677400	-3.78379400
H	1.34241600	0.03704300	-3.14503300
B	-1.89396300	-1.61556800	-0.54763700
H	-1.01564400	-2.30945500	-0.91521200
B	-4.15361700	0.61879100	0.54519000
H	-4.92118200	1.44452000	0.92975300
B	-3.22555400	-1.12611700	-1.55603900
H	-3.28224400	-1.53287300	-2.67225500
B	-2.77008100	0.12571500	1.55955700
H	-2.45660600	0.58424200	2.61194900
B	-3.51036700	-2.12694600	-0.09071700
H	-3.81828900	-3.27497200	-0.16405000
B	-3.62748800	0.54354400	-1.16879800
H	-3.95795000	1.31471500	-2.01199900
B	-2.52899700	1.08089700	0.06469900
H	-2.08378600	2.18277300	0.08717000
B	-4.60960800	-0.77574100	-0.47393700
H	-5.73341900	-0.95996800	-0.82400500
B	-4.06746300	-1.03960200	1.21812100
H	-4.80064200	-1.40967900	2.08137300
B	-2.37283300	-1.56954000	1.16698500
H	-1.78629700	-2.24793200	1.94976400
Si	1.06221100	2.76798800	-0.84814200
C	1.04581200	4.42653900	0.02947900
H	0.03301900	4.74971200	0.29436800
H	1.45690500	5.18006600	-0.65366900
H	1.66482200	4.43415900	0.93386300
C	2.79788800	2.18684000	-1.21425800
H	3.43669900	2.20365700	-0.32715400
H	3.23805800	2.85459400	-1.96601500
H	2.80259500	1.17451100	-1.62044800
C	-0.12126200	2.64063800	-2.27887800
H	-0.64409900	1.67332900	-2.25231000
H	0.41399800	2.73116300	-3.23111000
H	-0.88258600	3.42611500	-2.22822400
N	0.38695600	1.59111600	0.49418900
N	-0.12216000	2.16793500	1.49093100
N	-0.53206700	2.66545600	2.41570600

Int7

SCF Done: -1803.034578

Si	-3.21645600	-0.61568200	-0.72534000
Si	-1.94794000	0.58783500	1.81183100
N	-1.76690900	0.07071400	0.10393400
C	0.79771600	-0.72923400	-0.27012700
C	2.37267900	-0.10929200	-0.03799400
C	-2.75458000	-1.34865500	-2.40094500
H	-2.51360600	-0.59888300	-3.16032700
H	-3.63438600	-1.89828900	-2.75983700
H	-1.92437500	-2.05709000	-2.34613500
C	-3.86915900	-2.02735000	0.34382800
H	-3.08609300	-2.77707000	0.50447900
H	-4.70732000	-2.51827900	-0.16606500
H	-4.22717800	-1.69888900	1.32472500
C	-3.69097100	1.25878000	2.10570000
H	-3.94400300	2.07178200	1.41787400
H	-3.70745400	1.66914300	3.12375900
H	-4.48043000	0.50337200	2.05033900
C	-4.57411700	0.66163400	-1.04393200
H	-5.22051800	0.82832200	-0.17850700
H	-5.20635300	0.29975300	-1.86468100
H	-4.16074700	1.62747100	-1.35257200
B	-0.53927700	0.10810500	-0.63253600
C	-1.68432200	-0.78436100	3.07887600
H	-2.30216400	-1.65807500	2.84573100
H	-1.99199500	-0.41384900	4.06548100
H	-0.64908900	-1.12229300	3.15368500
C	-0.81150800	2.06107600	2.12801000
H	0.23551800	1.87336300	1.88792600
H	-0.85853100	2.33495900	3.18941900
H	-1.14393500	2.93109800	1.54924800
B	1.49642300	-0.79366800	1.28347000
H	1.10824700	-0.07005800	2.12858200
B	2.61642700	-2.54774500	-1.37285800
H	2.99095500	-3.12605300	-2.34166800
B	3.22419700	-1.12993800	1.05184200
H	3.99582500	-0.61931000	1.79371500
B	0.89327800	-2.21545500	-1.11392700
H	0.02878300	-2.46914600	-1.88221100
B	2.11382800	-2.44886400	1.47997000
H	2.13935600	-2.94908800	2.55859200
B	3.53838100	-1.20408800	-0.68657000
H	4.52920500	-0.74894600	-1.14719300
B	2.00786700	-0.88386200	-1.51719500
H	1.90569500	-0.22429200	-2.49162900
B	3.37741200	-2.70763200	0.24235000
H	4.32521300	-3.39961200	0.43596000
B	1.74089400	-3.34349100	-0.02644200
H	1.48240400	-4.50449200	-0.03318100
B	0.58530200	-2.17088400	0.63117400
H	-0.50173100	-2.38527700	1.04545400
Si	2.83263900	1.78226100	-0.00926400
C	4.38664000	2.00137800	-1.04834700
H	5.24881400	1.48465400	-0.61644400
H	4.62625100	3.07052100	-1.10767300

H	4.24638100	1.62955700	-2.06883600
C	1.50050200	2.90023000	-0.73508800
H	1.35584100	2.70641500	-1.80101400
H	1.85343300	3.93375100	-0.62343600
H	0.53089800	2.83173800	-0.23662500
C	3.18636900	2.27434500	1.77582400
H	2.34287200	2.08759000	2.44704700
H	3.41076000	3.34761700	1.81297900
H	4.05288200	1.73600000	2.17158800
N	-0.42284100	0.77624100	-1.92377700
N	-1.26657800	1.57939900	-2.32231700
N	-1.96281100	2.35170200	-2.78368900

TS10

SCF Done: -1802.94464

Si	-2.61840000	-1.04168000	-1.23143600
Si	-2.40040100	0.40980400	1.57068900
N	-1.72752100	-0.06458200	-0.02209000
C	0.80064400	-1.07561600	-0.02045000
C	2.18955800	-0.16948300	-0.01402600
C	-1.60603200	-1.05554500	-2.82323900
H	-1.46818400	-0.03629900	-3.20559800
H	-2.16212000	-1.61311700	-3.58719300
H	-0.62288800	-1.51931600	-2.73289900
C	-2.94459300	-2.77200500	-0.56614600
H	-2.01401800	-3.25643700	-0.25320700
H	-3.41998700	-3.40479900	-1.32555400
H	-3.61332200	-2.73795200	0.30266000
C	-4.24524100	0.77772100	1.45523800
H	-4.48739000	1.50711300	0.67611400
H	-4.56209000	1.19791000	2.41816700
H	-4.84497800	-0.12153800	1.28160200
C	-4.24634700	-0.20012800	-1.69263900
H	-5.04392700	-0.33795000	-0.95884600
H	-4.59186200	-0.63041700	-2.64120000
H	-4.11157100	0.87612700	-1.85117000
B	-0.30265800	0.02573400	-0.15898100
C	-2.14107100	-0.96925000	2.82671700
H	-2.53531200	-1.92501100	2.46430500
H	-2.65381900	-0.72625800	3.76609300
H	-1.07974400	-1.11565200	3.05000800
C	-1.52374900	1.96307600	2.18793200
H	-0.44690800	1.81163900	2.30296400
H	-1.92586200	2.22631900	3.17394900
H	-1.67787600	2.82511700	1.52928400
B	1.60528900	-0.82766600	1.45781600
H	1.11553100	-0.09525800	2.25212300
B	2.75836300	-2.65212000	-1.13774000
H	3.14654100	-3.26508100	-2.08042400
B	3.32721700	-0.87262100	1.05805900
H	4.08178600	-0.18001000	1.65587700
B	1.03149100	-2.61379600	-0.71740600
H	0.15086400	-3.10134300	-1.34314000
B	2.51525100	-2.31800200	1.73554800
H	2.73395500	-2.68519700	2.84568700
B	3.47765800	-1.07842000	-0.69002600
H	4.32820600	-0.52613700	-1.30281200
B	1.85261200	-1.15187500	-1.36772800
H	1.51753800	-0.63049600	-2.37769900
B	3.68718200	-2.48409400	0.38751800
H	4.75667800	-2.98342800	0.53854600
B	2.17132500	-3.42872900	0.37060300
H	2.15566600	-4.61085100	0.50666800
B	0.88070900	-2.41157000	1.03766000
H	-0.11025300	-2.74944300	1.59364000
Si	1.91977200	2.33282600	-0.20994000
C	3.22093200	2.20231800	-1.56453400
H	4.13678400	1.73243300	-1.19791000
H	3.46589300	3.19965700	-1.94706900
H	2.85912600	1.59375100	-2.40007300
C	1.18564800	4.08424900	-0.49199800
H	0.86607200	4.29064900	-1.52007200
H	2.00426500	4.78328000	-0.27617300
H	0.36211000	4.34685200	0.18363900
C	2.51123200	2.48506700	1.57298300
H	1.66439100	2.46389500	2.26717500
H	2.99065600	3.46584300	1.68112500
H	3.21972800	1.71711900	1.88093900
N	0.37645800	1.29889700	-0.45237100
N	-0.87325100	2.17969100	-0.97703000
N	-1.35073900	2.84947400	-1.73072900

Int8

SCF Done: -1802.952416

Si	0.34540200	1.48220000	-1.67598800
Si	0.31844300	2.03413000	1.40125000
N	-0.02237900	1.00567900	-0.00482100
C	-2.06678600	-0.77249100	-0.66157500
C	-2.17409800	-0.61447500	0.90704300
C	-0.92798200	2.68380700	-2.37494800
H	-1.91773100	2.21864400	-2.42251600
H	-0.64610500	2.97942000	-3.39377100
H	-1.01468700	3.59411100	-1.77272000
C	2.04764900	2.31260400	-1.74431300
H	2.07176800	3.26101900	-1.19599100
H	2.30418200	2.53407400	-2.78770800
H	2.83807600	1.67302100	-1.33469400
C	2.17276000	2.08694700	1.78725800
H	2.56567500	1.08538500	1.99531700
H	2.34403700	2.70043100	2.68111200
H	2.76261500	2.52028400	0.97370200
C	0.36984100	-0.04755500	-2.78970100
H	0.87186600	-0.90871600	-2.33724200
H	0.88848400	0.18620400	-3.72793800
H	-0.64843700	-0.36103400	-3.03898900
B	-0.77191900	-0.22549900	0.15077800
C	-0.27728500	3.79487400	1.06732700
H	0.26447000	4.27356000	0.24337900
H	-0.13531100	4.41739200	1.95931300
H	-1.34410100	3.80193600	0.81776400
C	-0.54476300	1.35484500	2.93082600
H	-1.63379700	1.40455700	2.84719400
H	-0.24306400	1.94883900	3.80266900
H	-0.27857700	0.31105200	3.13180000
B	-2.99444100	0.59332400	-0.05045300
H	-2.51405400	1.66744900	-0.10855800
B	-3.92661500	-2.65352600	0.17682800
H	-4.24406800	-3.79717900	0.26101300
B	-3.72495900	-0.03419700	1.41185400
H	-3.82560400	0.67002100	2.36213200
B	-3.07013800	-2.02086600	-1.28705600
H	-2.71711200	-2.65544200	-2.22636300
B	-4.65218500	0.12582300	-0.13331200
H	-5.48192900	0.96743000	-0.27087100
B	-3.27375700	-1.75085600	1.60693500
H	-3.06763100	-2.20138100	2.68552700
B	-2.25826300	-2.23119100	0.25719500
H	-1.32254700	-2.93840300	0.38920200
B	-4.85483200	-1.31440200	0.89583500
H	-5.86733700	-1.51190400	1.48978000
B	-4.72674400	-1.47895500	-0.90116300
H	-5.64860700	-1.79545400	-1.58442100
B	-3.51339400	-0.29887300	-1.47391100
H	-3.45913300	0.21892600	-2.54172600
N	0.39973600	-1.47167300	0.41473400
N	1.52467600	-1.43035000	0.66772500
N	2.66694600	-1.41715800	1.01829500
Si	4.20584700	-1.46532800	0.03956700
C	5.13619200	0.08215900	0.52977600
H	4.59124800	0.98583700	0.24017200
H	5.29802700	0.12019900	1.61233800
H	6.11729700	0.10265900	0.03956200
C	5.05764200	-3.03528800	0.60250300
H	6.03730800	-3.13155200	0.11859500
H	5.21621400	-3.03061400	1.68634400
H	4.46816400	-3.92316200	0.34881400
C	3.72803800	-1.50041700	-1.77304500
H	3.06480300	-2.34150800	-2.00521700
H	3.22743600	-0.57742200	-2.08100800
H	4.62858000	-1.61216900	-2.38969500

TS11

SCF Done: -1802.934687

Si	-0.82799600	2.56204500	-1.16474000
Si	0.79957400	1.85624200	1.41096500
N	-0.15582600	1.33718900	-0.02478100
C	-1.91088600	-0.73243000	-0.36011500
C	-1.74495100	-1.67804900	0.94222900
C	-2.31721900	3.45404800	-0.43164600
H	-2.93427100	2.77633400	0.16394100
H	-2.94727900	3.85970100	-1.23256300
H	-2.01119100	4.28855000	0.20808600
C	0.53348200	3.82166200	-1.53796700
H	0.84022300	4.41262000	-0.66921200
H	0.15705100	4.52482700	-2.29187800
H	1.42513300	3.33911700	-1.95457900
C	2.58508900	2.22137000	0.90604800
H	3.03572400	1.35072200	0.41758400
H	3.18881300	2.44845400	1.79390200
H	2.66385100	3.06845600	0.21826100
C	-1.22083300	1.70668600	-2.79356700
H	-0.34210100	1.17306100	-3.17659200
H	-1.49423200	2.46478200	-3.53827500
H	-2.04610300	0.99618400	-2.72595600
B	-0.51329100	-0.01589800	-0.17672400
C	0.00362700	3.39439300	2.15198900

H	0.02819000	4.26562600	1.49108400
H	0.53780400	3.66205100	3.07213700
H	-1.04120500	3.19887700	2.41672500
C	0.79372600	0.49648500	2.72024300
H	-0.12751200	-0.09521300	2.70553800
H	0.87856300	0.96475500	3.70837300
H	1.63675800	-0.19559300	2.61965000
B	-2.75671200	-0.29987000	1.07426600
H	-2.37495500	0.64450000	1.68542400
B	-3.40307700	-2.99261800	-0.86361000
H	-3.59361900	-3.94458600	-1.55300000
B	-3.22840900	-1.83178200	1.77952000
H	-3.22619700	-1.94591000	2.96267500
B	-2.89684100	-1.43702900	-1.57797200
H	-2.64829000	-1.23125600	-2.72172500
B	-4.40554500	-0.85960600	0.82843000
H	-5.31739600	-0.29132000	1.34145500
B	-2.62521200	-3.13692800	0.74420000
H	-2.20638800	-4.14394700	1.21688900
B	-1.76288200	-2.40966800	-0.60271200
H	-0.75691700	-2.83344800	-1.07079000
B	-4.33462200	-2.63700300	0.62298600
H	-5.21501600	-3.34261300	1.00498400
B	-4.49524900	-1.57473300	-0.81129900
H	-5.49204200	-1.51669300	-1.46098200
B	-3.50927000	-0.12922100	-0.53323500
H	-3.68303400	0.95308200	-0.98403600
N	0.54463400	-1.07631100	0.07061200
N	1.69829100	-1.23115400	0.19003100
N	2.77363600	-1.61187300	0.58058600
Si	4.30628500	-1.52388100	-0.42133700
C	5.53474500	-0.63864100	0.68053700
H	5.22347200	0.39152700	0.88303100
H	5.64011900	-1.15684800	1.64002300
H	6.52237800	-0.60710000	0.20476400
C	4.76319100	-3.31283200	-0.73481100
H	5.71335000	-3.37865200	-1.27880100
H	4.87577700	-3.85878000	0.20816700
H	3.99731500	-3.82120800	-1.33077600
C	3.88458000	-0.58099000	-1.99032200
H	3.09249700	-1.08433800	-2.55713800
H	3.55066000	0.43979800	-1.77205100
H	4.76308000	-0.51059300	-2.64287900

Int9

SCF Done: -1803.005957

Si	-3.47188000	0.67092900	-0.63503500
Si	-1.27581000	2.47559800	0.68266300
N	-1.79962200	0.87941100	-0.00407500
C	-0.86238200	-1.80414200	-0.03759700
C	0.74242300	-1.62373200	0.18782400
C	-4.71842800	0.63352500	0.77823200
H	-4.85698500	1.60019400	1.26905600
H	-4.40703700	-0.09256600	1.53876300
H	-5.69453300	0.31049700	0.39499700
C	-3.81382400	2.07299200	-1.85356200
H	-3.75319600	3.07429500	-1.41784700
H	-4.82084000	1.95537100	-2.27290700
H	-3.10214700	2.02794700	-2.68666900
C	-0.46173000	3.48298900	-0.67976000
H	0.40602700	2.94546200	-1.07276200
H	-0.11629500	4.45189400	-0.29858600
H	-1.15919100	3.67301300	-1.50356000
C	-3.63457200	-0.93935900	-1.58577100
H	-2.83329000	-1.08996000	-2.31598300
H	-4.58408500	-0.91571100	-2.13549300
H	-3.65816100	-1.81026200	-0.92594500
B	-0.87007800	-0.15721400	-0.04584300
C	-2.75880900	3.41298100	1.38667400
H	-3.56520800	3.62579600	0.68032900
H	-2.38184500	4.37821500	1.74923700
H	-3.18594400	2.88880300	2.24839200
C	-0.14290100	2.19612300	2.16422400
H	-0.55612900	1.42948600	2.83032900
H	-0.07869600	3.12964100	2.73753400
H	0.87002800	1.89802900	1.88885700
B	-0.22561700	-2.07533200	1.56564700
H	-0.42955200	-1.22120200	2.35967000
B	0.43886800	-3.99493600	-1.15130900
H	0.66640300	-4.66600300	-2.10570800
B	1.40036900	-2.73220900	1.28954000
H	2.28103600	-2.40180300	2.00831700
B	-1.17744900	-3.26849200	-0.88884800
H	-2.08360800	-3.33258600	-1.64519800
B	-0.01022500	-3.78955400	1.71196100
H	-0.09206400	-4.31076800	2.77699900
B	1.67800000	-2.86556100	-0.48767000
H	2.74371700	-2.63851400	-0.94500600
B	0.22000800	-2.27840700	-1.32452200
H	0.27716000	-1.54585800	-2.25322200
B	1.16895900	-4.29119100	0.45907800
H	1.91906900	-5.19529300	0.64245500
B	-0.59132100	-4.54452300	0.20312400
H	-1.08921000	-5.62406500	0.20388100
B	-1.45689800	-3.12612900	0.86423400
H	-2.56149900	-3.07172500	1.29592300
N	0.60259600	-0.17090200	0.08682800
N	1.59275000	0.72570400	-0.11624500
N	2.74398700	0.23411800	-0.07075300
Si	4.10577600	1.37765900	-0.33858100
C	5.16644300	1.25104300	1.21139200
H	4.62293500	1.60868400	2.09359600
H	5.46021700	0.21199700	1.39710600
H	6.07987700	1.85016400	1.11319100
C	5.01702300	0.71639300	-1.84682700
H	5.93399900	1.28739500	-2.03668700
H	5.29463700	-0.33361000	-1.70201600
H	4.39087000	0.77679400	-2.74450100
C	3.48735500	3.13661400	-0.61298300
H	2.86692700	3.20382700	-1.51338400
H	2.88046900	3.47973800	0.23231100
H	4.32829800	3.83095200	-0.73044400

TS12

SCF Done: -1802.973712

Si	0.35812100	-2.45092900	1.24399300
Si	1.63363900	-1.49007300	-1.47605400
N	0.56970600	-1.21222500	-0.05136700
C	-2.02959300	-0.18268600	0.15586000
C	-1.90709300	1.30422900	-0.46748400
C	-1.04144500	-3.63573800	0.82169300
H	-2.01552900	-3.13978500	0.85437200
H	-1.06324900	-4.46140800	1.54395400
H	-0.91725300	-4.06679000	-0.17787500
C	1.96010800	-3.42083300	1.46414200
H	2.20799600	-4.05815700	0.60912500
H	1.83830000	-4.07760800	2.33466700
H	2.81309200	-2.76375200	1.66287000
C	3.45752100	-1.66793400	-1.01492400
H	3.79178300	-0.93957000	-0.27199000
H	4.06104000	-1.51547100	-1.91879100
H	3.68821700	-2.66664100	-0.63381200
C	0.01045100	-1.53832900	2.85357500
H	0.84526900	-0.87824700	3.11394200
H	-0.11531100	-2.25884200	3.67116900
H	-0.89985700	-0.93341700	2.80933900
B	-0.40185600	-0.21167100	-0.09690400
C	1.06108400	-3.10009400	-2.28018400
H	1.17012100	-3.96483900	-1.61622800
H	1.65433200	-3.30009600	-3.18120200
H	0.00841100	-3.03733100	-2.57937100
C	1.42730100	-0.10103800	-2.72728800
H	0.39682800	-0.02388700	-3.08702300
H	2.06726800	-0.32095400	-3.59136500
H	1.71806800	0.87704900	-2.33652300
B	-2.57891200	0.06739100	-1.48425300
H	-1.87373800	-0.34291100	-2.34215900
B	-4.01088700	1.38403200	1.29889500
H	-4.51002000	1.84627600	2.27332200
B	-3.19761800	1.72849300	-1.48299000
H	-2.99989300	2.41682300	-2.42673500
B	-3.31836000	-0.27382300	1.28775400
H	-3.21442200	-1.00203800	2.21812200
B	-4.29695600	0.29465900	-1.38082300
H	-4.99404800	-0.01539700	-2.29202300
B	-3.01704000	2.40542500	0.17902100
H	-2.71184800	3.53908000	0.33009200
B	-2.29330700	1.16359600	1.22053200
H	-1.40987200	1.41365600	1.96870000
B	-4.58436400	1.74301200	-0.36426800
H	-5.51474200	2.45761700	-0.55620300
B	-4.77321800	0.09209300	0.32727300
H	-5.83224700	-0.36115400	0.62044900
B	-3.48119800	-0.93817800	-0.35488800
H	-3.49944000	-2.10928300	-0.53565000
N	-0.46308100	1.19927500	-0.58513500
N	0.24298500	2.42401400	-0.56450000
N	1.42758300	2.38177200	-0.26106900
Si	2.96705300	2.25160900	0.50614500
C	4.21399100	1.96083800	-0.87507600
H	4.23842600	2.82505400	-1.54802600
H	5.21772400	1.84002800	-0.44936100
H	3.99274900	1.07329400	-1.47274900
C	2.94700000	0.88422100	1.81144400
H	2.60960600	-0.06803200	1.39619300
H	3.93921800	0.74473100	2.25939700

H	2.25628900	1.15610700	2.61917900
C	3.34762400	3.87903200	1.37589300
H	3.40025400	4.70446700	0.65772400
H	2.57295400	4.12401500	2.11179900
H	4.30667700	3.82391700	1.90732900

Int10

SCF Done: -1802.981466

Si	-1.30214100	-2.30418700	-1.28848400
Si	-2.20124100	-1.06182900	1.43809300
N	-1.03804400	-1.15199900	0.06841500
C	1.74066300	-0.77260000	-0.19622700
C	1.94584700	0.68819900	0.45524800
C	-0.76494700	-4.03825600	-0.78989400
H	0.27693000	-4.02455100	-0.44739000
H	-0.82490300	-4.72117000	-1.64632300
H	-1.37829700	-4.45423700	0.01518000
C	-3.11961700	-2.22676500	-1.80185200
H	-3.83516700	-2.48006500	-1.01539500
H	-3.28316300	-2.92356700	-2.63348900
H	-3.36245900	-1.22150200	-2.16656500
C	-3.58998000	0.15174300	1.04214300
H	-3.22917400	1.18518300	1.06291500
H	-4.38763000	0.07032400	1.79074900
H	-4.03341400	-0.03785200	0.05867400
C	-0.32235100	-1.75504700	-2.79902200
H	-0.55917500	-0.72422600	-3.08226800
H	-0.59945400	-2.39907600	-3.64334500
H	0.75852400	-1.83472500	-2.66880500
B	0.14802600	-0.40717200	0.04933900
C	-2.91763000	-2.77856000	1.77499900
H	-3.47271100	-3.23210300	0.94994600
H	-3.60659300	-2.69156500	2.62498700
H	-2.12295100	-3.47225200	2.07155500
C	-1.33605200	-0.57706900	3.03584200
H	-0.63636800	-1.36008100	3.34869500
H	-2.09884000	-0.48685600	3.82036800
H	-0.79200700	0.36921400	2.99174400
B	2.23987800	-0.69434500	1.47228300
H	1.40174900	-0.91744900	2.27491600
B	4.11627700	0.27591600	-1.17936600
H	4.76953300	0.61170300	-2.11391000
B	3.23079200	0.77386400	1.55472700
H	3.15202600	1.47537500	2.50512200
B	3.03030300	-1.15522900	-1.25575200
H	2.83525200	-1.82067300	-2.21667700
B	3.95760700	-0.88871900	1.47759700
H	4.50377600	-1.36608600	2.41892200
B	3.33590200	1.49105000	-0.09499900
H	3.32073200	2.66995600	-0.22602100
B	2.39232700	0.48891500	-1.20947800
H	1.65968000	0.95514600	-2.01179800
B	4.65293900	0.46012600	0.51945700
H	5.71492900	0.92160800	0.78880600
B	4.47639700	-1.17694300	-0.20553500
H	5.40854300	-1.87019500	-0.45736400
B	2.93589500	-1.87380000	0.36815900
H	2.64361400	-3.01633400	0.50186900
N	0.51734200	0.97413300	0.43016600
N	-0.00880500	1.88020000	1.47251200
N	-0.88098600	2.65601200	1.10635500
Si	-1.39619000	3.12523100	-0.58953300
C	-2.99351100	4.06509500	-0.28171900
H	-2.85299100	4.83593200	0.48320400
H	-3.34201300	4.55198600	-1.20054500
H	-3.79020100	3.39388700	0.05998200
C	-1.67982400	1.75312800	-1.84687800
H	-2.20517600	0.89880700	-1.41313400
H	-2.28383800	2.13743300	-2.67884800
H	-0.72566300	1.39934300	-2.24554600
C	0.02289100	4.24724600	-1.10966800
H	0.19488700	5.04038000	-0.37451000
H	0.94906600	3.67082300	-1.21321500
H	-0.19423300	4.71904100	-2.07567800

TS13

SCF Done: -1802.970254

Si	-1.93565500	-1.93685300	-1.25698800
Si	-2.49820600	-0.39991900	1.39006300
N	-1.39922800	-0.85688800	0.06261700
C	1.39292700	-1.27758700	-0.05034200
C	1.92729100	0.22619000	0.31561600
C	-2.04735800	-3.72710500	-0.67828800
H	-1.09296900	-4.03481200	-0.23395500
H	-2.24905000	-4.39221100	-1.52724000
H	-2.83290400	-3.88652500	0.06635400
C	-3.59090400	-1.29626600	-1.91829200
H	-4.38356400	-1.21316800	-1.16957000
H	-3.95279100	-1.96518300	-2.70917700
H	-3.45149400	-0.30493900	-2.36724400
C	-3.39198500	1.22699600	1.01482400
H	-2.70622200	2.08032200	0.97667400
H	-4.13229700	1.43982500	1.79596700
H	-3.92404000	1.17921400	0.05753700
C	-0.74790200	-1.85227300	-2.71690900
H	-0.58437100	-0.82510200	-3.05899900
H	-1.19343100	-2.40760500	-3.55228700
H	0.22641500	-2.29773700	-2.50780400
B	-0.03787300	-0.43516800	0.07633100
C	-3.80200700	-1.74110300	1.67403300
H	-4.42646400	-1.98230200	0.81027800
H	-4.46904700	-1.39732200	2.47509200
H	-3.32974500	-2.66706800	2.02033100
C	-1.58343200	-0.27977700	3.03115500
H	-1.17793000	-1.25947500	3.30844200
H	-2.29428500	0.01772000	3.81295900
H	-0.75760500	0.43207200	3.03504100
B	1.93280500	-0.99348200	1.56878700
H	1.10582600	-0.88347600	2.40501400
B	3.91780600	-1.01785400	-1.16387800
H	4.60778700	-1.02030800	-2.13291300
B	3.24773100	0.17669100	1.40152200
H	3.36824900	1.03972600	2.20796000
B	2.53206000	-2.14669300	-0.98788700
H	2.16363300	-2.91328100	-1.81484000
B	3.55979800	-1.58991300	1.65761800
H	3.99276400	-1.99457500	2.68892500
B	3.48231400	0.52150900	-0.33467800
H	3.79389900	1.60670200	-0.69119000
B	2.28723000	-0.41364800	-1.25080800
H	1.67590400	0.04756900	-2.15508700
B	4.53092800	-0.65745000	0.48300400
H	5.67947100	-0.41412600	0.67737900
B	3.95809700	-2.30894600	0.07261400
H	4.69762500	-3.23543800	-0.02409000
B	2.30962500	-2.49773600	0.73356000
H	1.76383900	-3.49214300	1.08557700
N	0.61241000	0.85113400	0.30820100
N	0.16313100	2.33212100	1.98375900
N	-0.15258200	3.16492900	1.29613500
Si	-0.12213200	3.19833800	-0.79993200
C	-1.03785400	4.84603900	-0.76750800
H	-0.51603600	5.59590300	-0.16317200
H	-1.12554600	5.23197100	-1.79162000
H	-2.05425900	4.73664300	-0.37067000
C	-1.13339600	2.01941200	-1.83860900
H	-1.80899700	1.40908600	-1.23764000
H	-1.72497300	2.58633900	-2.56801800
H	-0.45305700	1.33504400	-2.35205800
C	1.68405000	3.45891100	-1.18279300
H	2.28995700	3.53186200	-0.27557900
H	2.06922100	2.62033800	-1.76533800
H	1.80508900	4.38827400	-1.75228700

References

[1] Zhang, H.; Wang, J.; Yang, W.; Xiang, L.; Sun, W.; Ming, W.; Li, Y.; Lin, Z.; Ye, Q. *J. Am. Chem. Soc.* **2020**, *142*, 17243–17249.

[2] Wang, J.; Jia, P.; Sun, W.; Wei, Y.; Lin, Z.; Ye, Q. *Inorg. Chem.* **2022**, *61*, 8879–8886.

[3] Sheldrick, G. *Acta Cryst.* **2015**, *A71*, 3–8.

[4] Sheldrick, G. *Acta Cryst.* **2008**, *A64*, 112–122.

[5] Frisch, M. J.; Trucks, G. W.; Schlegel, H. B.; Scuseria, G. E.; Robb, M. A.; Cheeseman, J. R.;Scalmani, G.; Barone, V.; Mennucci, B.; Petersson, G. A.; Nakatsuji, H.; Caricato, M.; Li, X.;Hratchian, H. P.; Izmaylov, A. F.; Bloino, J.; Zheng, G.; Sonnenberg, J. L.; Hada, M.; Ehara, M.;Toyota, K.; Fukuda, R.; Hasegawa, J.; Ishida, M.; Nakajima, T.; Honda, Y.; Kitao, O.; Nakai, H.;Vreven, T.; Montgomery, Jr., J. A.; Peralta, J. E.; Ogliaro, F.; Bearpark, M.; Heyd, J. J.; Brothers, E.;Kudin, K. N.; Staroverov, V. N.; Kobayashi, R.; Normand, J.; Raghavachari, K.; Rendell, A.; Burant,J. C.; Iyengar, S. S.; Tomasi, J.; Cossi, M.; Rega, N.; Millam, J. M.; Klene, M.; Knox, J. E.; Cross, J.B.; Bakken, V.; Adamo, C.; Jaramillo, J.; Gomperts, R.; Stratmann, R. E.; Yazyev, O.; Austin, A. J.;Cammi, R.; Pomelli, C.; Ochterski, J. W.; Martin, R. L.; Morokuma, .; Zakrzewski, V. G.; Voth, G.A.; Salvador, P.; Dannenberg, J. J.; Dapprich, S.; Daniels, A. D.; Farkas, Ö.; Foresman, J. B.; Ortiz,J. V.; Cioslowski, J.; Fox, D. J. Gaussian 09, Revision D.01; Gaussian, Inc.: Wallingford, CT, **2009**.

[6] a) Lee, C.; Yang, W.; Parr, R. G. *Phys. Rev. B* **1988**, *37*, 785–789; b) Becke, A. D. *J. Chem. Phys.* **1993**, *98*, 5648–5652.

[7] a) Ditchfield, R.; Hehre, W. J.; Pople, J. A. *J. Chem. Phys.* **1971**, *54*, 724-728; b) Hehre, W. J.; Ditchfield, R.; Pople, J. A. *J. Chem. Phys.* **1972**, *56*, 2257-2261; c) Hariharan, P. C.; Pople, J. A. *Theor. Chim. Acta* **1973**, *28*, 213-222; d) Dill, J. D.; Pople, J. A. *J. Chem. Phys.* **1975**, *62*, 2921-2923; e) Rassolov, V. A.; Ratner, M. A.; Pople, J. A.; Redfern, P. C.; Curtiss, L. A. 6-31G* basis set for third-row atoms. *J. Comput. Chem.* **2001**, *22*, 976-984.

[8] Grimme, S. *Chem. Eur. J.* **2004**, *10*, 3423–3429.

[9] a) Fukui, K. *J. Phys. Chem.* **1970**, *74*, 4161–4163; b) Fukui, K. *Acc. Chem. Res.* **1981**, *14*, 363–368.

[10] Marenich, A. V.; Cramer, C. J.; Truhlar, D. G. *J. Phys. Chem. B.* **2009**, *113*, 6378–6396.

[11] a) Krishnan, R.; Binkley, J. S.; Seeger, R.; Pople, J. A. *J. Chem. Phys.* **1980**, *72*, 650-654; b) McLean, A. D.; Chandler, G. S. *J. Chem. Phys.* **1980**, *72*, 5639-5648; c) Francl, M. M.; Pietro, W. J.; Hehre, W. J.; Binkley, J. S.; Gordon, M. S.; DeFrees, D. J.; Pople, J. A. *J. Chem. Phys.* **1982**, *77*, 3654-3665.
